# Blood‐based biomarkers for Alzheimer's disease in Down syndrome: A systematic review and meta‐analysis

**DOI:** 10.1002/alz.70135

**Published:** 2025-04-12

**Authors:** Yajing Zhou, Rory Sheehan, Lizhi Guo, Andre Strydom

**Affiliations:** ^1^ Department of Forensic and Neurodevelopmental Sciences Institute of Psychiatry Psychology & Neuroscience King's College London London UK; ^2^ Department of Psychology The Chinese University of Hong Kong Hong Kong China

**Keywords:** Alzheimer's disease, amyloid, biomarkers, blood, Down syndrome, neurodegeneration, neuroinflammation, plasma, tau

## Abstract

Individuals with Down syndrome (DS) are at high risk of Alzheimer's disease (AD), displaying AD pathology similar to the general population. This study evaluated AD‐related blood biomarkers in DS within the AT(N) framework through a systematic review and meta‐analysis of studies published between 2017 and October 2024. The meta‐analysis focused on plasma amyloid beta (Aβ)42, Aβ40, total tau (t‐tau), phosphorylated tau (p‐tau)181, neurofilament light chain (NfL), and glial fibrillary acidic protein (GFAP) levels, comparing 2109 DS individuals and 1006 euploid controls. Plasma Aβ42, Aβ40, NfL, and GFAP levels were significantly elevated in DS compared to euploid controls, while the Aβ42/40 ratio was reduced. In DS‐AD individuals, Aβ42 and t‐tau levels were elevated, with p‐tau181, NfL, and GFAP consistently high across clinical subgroups. Notably, Aβ40 and the Aβ42/40 ratio changed significantly in preclinical AD, while t‐tau increased in clinical AD. Incorporating inflammation (I) markers highlights neuroinflammation's role in DS‐AD progression, supporting the blood‐based AT(N)I framework for early AD detection and monitoring in DS.

**Highlights:**

- We reviewed 58 studies on Down syndrome (DS) blood biomarkers and a meta‐analysis of 18 using single molecule array.
- Plasma amyloid beta (Aβ)42, Aβ40, neurofilament light chain (NfL), and glial fibrillary acidic protein (GFAP) levels were elevated in DS compared to controls.
- DS‐Alzheimer's disease (AD) individuals showed higher Aβ42, total tau (t‐tau), phosphorylated tau (p‐tau)181, NfL, and GFAP levels.
- Plasma p‐tau181, NfL, and GFAP were elevated across all clinical subgroups.
- Aβ40 and Aβ42/40 ratio changed in preclinical AD; t‐tau rose in clinical AD.

## 1 INTRODUCTION

Down syndrome (DS), also referred to as trisomy 21, is the most common genetic cause of intellectual disability, and there are estimated to be 5.8 million people with DS globally.^1^ Advances in medical care, screening and early intervention for comorbidities, and improvements in living conditions have substantially extended the average lifespan of those with DS to > 60 years, compared to just 12 years in 1929.^2^ However, this extended lifespan has led to a lifetime risk of Alzheimer's disease (AD) > 90% among individuals with DS.^3^ A population‐based cohort study of 10,204 DS individuals between 1990 and 2020 reported that those with DS have an incidence rate ratio of 94.7 for dementia compared to controls from the general population.^4^ Nearly 80% of those aged ≥ 65 are clinically diagnosed with dementia.^5^ Consequently, AD has become the leading cause of death among individuals with DS.^6^, ^7^

In individuals with DS, changes in cerebrospinal fluid (CSF) amyloid beta (Aβ)42/40 ratio and plasma neurofilament light chain (NfL) levels typically begin around the age of 30, followed by alterations in amyloid positron emission tomography (PET) uptake and CSF phosphorylated tau (p‐tau) levels in subsequent years.^8^, ^9^, ^10^ By ≈ age 50, hippocampal atrophy and cognitive decline become evident, along with the clinical diagnosis of prodromal AD.^8^, ^9^ This indicates that the progression of AD pathology in individuals with DS, from the initial amyloid deposition to the clinical onset of prodromal AD, spans ≈ 20 years.^10^ During this prolonged process, there are currently no proven pharmacological or lifestyle interventions that can delay the onset of dementia. However, given the impact of AD on people with DS and their families, it is likely that even a relatively short delay in the onset of AD could yield major benefits, both in quality of life and in overall costs of care.^11^ It has been estimated that delaying the onset of dementia by just 5 years could lead to a substantial reduction—up to 40%—in both its prevalence and associated economic burden.^12^ Therefore, it is of paramount significance to have prompt and reliable diagnosis in the DS population, as well as the means to track the progression of AD in anticipation of anti‐amyloid therapies. Tracking the progression of AD in individuals with DS can be challenging. While PET scans can detect amyloid and tau deposition, their high cost presents a significant financial impediment to routine use.^13^, ^14^ Furthermore, CSF analysis, though effective, is often not feasible for many individuals with DS, as lumbar punctures can be difficult to tolerate.^13^, ^14^ Thus, standardized and effective diagnostic methods for AD are urgently required, not only for the specific population of people with DS but for all other communities at risk for AD.

In 2018, the National Institute on Aging and the Alzheimer's Association (NIA‐AA) proposed the AT(N) framework,^14^ aimed at moving beyond conventional clinical indicators, such as neuropsychological test scores, and instead focused on pathological and physiological markers of AD. The biomarkers are classified into three categories: Aβ deposition (A), pathological tau (T), and neurodegeneration (N). Recent protocols^15^ have explored the development of blood‐based biomarkers (BBM) in the AT(N) framework, which has demonstrated promising diagnostic performance, and in addition have attempted to expand the initial framework to incorporate additional components, such as inflammatory markers (e.g., glial fibrillary acidic protein [GFAP]), to reflect the heterogeneity of AD better. Extensive research^16^, ^17^, ^18^ has been undertaken on the AT(N) framework in populations at a high risk of AD, especially those with autosomal‐dominant AD (ADAD),^19^ which demonstrated the potential for detecting AD at early stages. However, less attention has been focused on individuals with DS, despite their elevated risk for developing AD, and the potential for BBM in this population.

**RESEARCH IN CONTEXT**

1. **Systematic review**: While previous systematic reviews and meta‐analyses have examined blood‐based biomarkers of AD in DS, over 40 new relevant studies have been published in recent years, necessitating an updated review. The authors reviewed the current state of evidence regarding blood‐based biomarkers of Alzheimer's disease in Down syndrome using multiple databases (PubMed, Embase, Web of Science, and Scopus) and a meta‐analysis, including all original studies that measured plasma biomarkers using the SIMOA platform and compared plasma biomarker levels between adult (≥18 years old) DS individuals with and without dementia and between DS individuals and the control group.
2. **Interpretation**: The blood‐based AT(N) biomarkers showed great potential for the clinical diagnosis of AD, and we also found that the inflammation biomarker, GFAP, performed well in distinguishing AD stages. This suggests that future models could consider incorporating an AT(N)I framework, where inflammation (I) biomarkers like GFAP are integrated alongside amyloid, tau, and neurodegeneration markers to provide a more comprehensive understanding of disease progression and diagnosis in AD, particularly in high‐risk groups like DS.
3. **Future directions**: Additional data and research on plasma biomarkers and DS are necessary, in more diverse DS cohorts to enable more accurate estimates of biomarker differences. Future work should include amyloid, tau, neurodegeneration, and inflammation blood biomarkers in DS individuals. Future studies should consistently report mean and standard deviation across different AD clinical classifications and within subgroups to allow for meta-analysis. In addition, longitudinal studies are required to better understand the clinical utility and predictive value of plasma biomarkers.

Research^20^, ^21^, ^22^ comparing people with DS and those with ADAD suggested that, although there are some minor variations, the overall progression of amyloid and tau pathology is similar between these two groups. AD in DS is linked to the presence of three copies of chromosome 21 in persons with DS. This extra copy includes the amyloid precursor protein (*APP*) gene, which results in an excessive amount of APP.^23^, ^24^ In ADAD, there are mutations in the *APP*, presenilin 1 (*PSEN1*), and presenilin 2 (*PSEN2*) genes.^25^ All these contribute to amyloid plaque deposition through several mechanisms or pathways, a hallmark of AD pathology.

There has been significant progress recently in identifying BBM related to the AT(N) framework, such as plasma Aβ ratios, p‐tau181, p‐tau 217, GFAP, and NfL. These have shown promise for early disease prediction in high‐risk populations such as those with ADAD.^15^, ^26^, ^27^, ^28^, ^29^, ^30^ High‐risk populations are ideal for exploring the clinical and predictive value of such biomarkers, including establishing the potential for applying the blood‐based AT(N) framework in the DS population.^15^

We aimed to undertake a systematic review and meta‐analysis to update previous reviews^31^, ^32^ and evaluate the scientific literature for prospective blood biomarkers that have been investigated as part of the AT(N) model in DS, with the addition of other BBM, such as those related to neuroinflammation. Our aim was to explore the feasibility of applying the blood‐based AT(N) framework for diagnosing AD in adults with DS. To minimize variability in assay techniques, our meta‐analysis focused exclusively on studies using single molecule array (SIMOA) technology for plasma biomarker measurements, ensuring greater sensitivity and comparability across studies.

## 2 METHODS

### 2.1 Search strategy

We followed the Preferred Reporting Items for Systematic Reviews and Meta‐Analyses (PRISMA) guidelines,^33^, ^34^, ^35^ and restricted our systematic review and meta‐analysis to studies published from 2017 onward to update systematic reviews published in 2019.^31^, ^32^ Our review was prospectively registered with PROSPERO (registration number: CRD42024507097). We searched PubMed, Embase, Web of Science, and Scopus for peer‐reviewed articles published in English between 2017 and October 2024. The systematic search started by using essential phrases and Boolean operators, specifically “(Down syndrome OR Down's syndrome OR trisomy 21) AND (Alzheimer's OR Alzheimer OR dementia) AND (plasma OR serum OR blood) AND (amyloid OR tau OR neurofilament light OR glial fibrillary acidic protein OR inflammation OR biomarker OR complement OR cytokines)” (Table S3 in supporting information). The reference lists of included papers were examined to uncover additional relevant articles not identified in the database search.

### 2.2 Inclusion and exclusion criteria

The review was conducted in two stages. First, we undertook a systematic review to identify all original research that examined blood biomarkers associated with AD in individuals with DS. The review included research that spanned across all age groups and examined biomarkers in both blood plasma and serum. Crucially, the systematic review was not limited by the type of assay technology used; it considered studies that used any method for detecting blood biomarkers.

Then, to reduce the impact of variability caused by using different platforms and assays when measuring the concentration of blood biomarkers,^31^, ^36^, ^37^ and considering that SIMOA, an innovative ultrasensitive detection technology, provides greater sensitivity and specificity for plasma biomarkers compared to traditional enzyme‐linked immunosorbent assay (ELISA),^38^ we conducted a meta‐analysis which exclusively included studies using SIMOA technology to measure plasma biomarkers levels in patients with DS. This meta‐analysis aimed to investigate the potential of using the blood‐based AT(N) framework to diagnose AD in DS, specifically, (1) compare the differences in Aβ (Aβ42, Aβ40, Aβ42/40 ratio), tau protein (t‐tau, p‐tau 181), NfL, and GFAP plasma levels between individuals with DS and euploid controls; (2) explore the relationship between these biomarkers and dementia status in DS individuals; (3) compare the differences in these biomarkers among DS clinical subgroups (cognitively stable [CS], prodromal AD, and AD).

The included studies had to compare plasma biomarker levels between various groups: adult (≥18 years old) DS individuals with and without AD; or adult individuals with DS and euploid controls; or adult individuals with DS among clinical subgroups (CS, prodromal AD, and AD). The categorization of clinical subgroups was used in several studies,^16^, ^39^, ^40^, ^41^ with individuals with DS being classified as “CS” if there are no clinical or neuropsychological indications of AD or of significant cognitive decline. In this report, we classified DS and CS as DS without AD. Individuals who display initial symptoms or have a deterioration in cognitive abilities that is more significant than that which is typically observed for their age, indicating possible AD but not reaching the requirements for dementia, were typically categorized as having “prodromal AD (pAD).” Individuals who have developed symptoms of dementia sufficient to meet any recognized diagnostic criteria in included studies were classified as having “DS‐AD dementia.” Importantly, to be included, studies must have diagnosed AD by expert clinicians using one of the following set of criteria: The Diagnostic and Statistical Manual of Mental Disorders, Fourth Edition (DSM‐IV) criteria; DSM, Fifth Edition (DSM‐V) criteria; or the NIA‐AA 2018 criteria. We classified people who were identified as DS without AD or DS with CS as the DS group, meaning that individuals with clinical indications of AD were excluded from this comparison. However, in cases in which individuals with DS did not undergo clinical classification for AD they were included in the DS group for this meta‐analysis.

### 2.3 Study selection and data extraction

**FIGURE 1:**
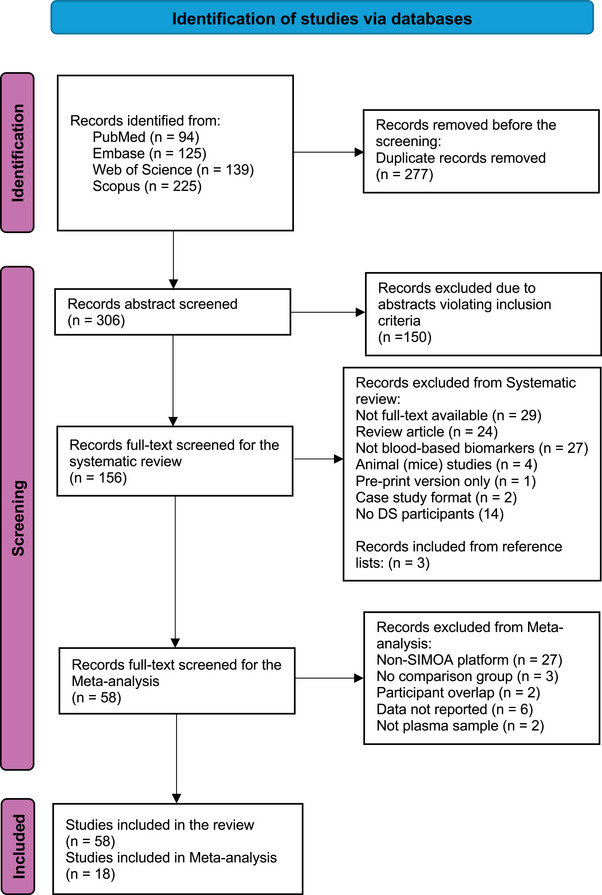
Flowchart illustrating the study selection and inclusion process. DS, Down syndrome; SIMOA, single molecule array.

A total of 583 studies were identified by searching electronic databases PubMed, Embase, Web of Science, and Scopus (Figure 1). After removing duplicates, the abstracts of all the potentially relevant studies (*n* = 306) were reviewed and studies were excluded if they did not report original data on blood biomarkers in people with DS. Full‐text articles were independently reviewed by two reviewers against predefined inclusion and exclusion criteria (Table S4 in supporting information). When discrepancies between reviewers arose, consensus was reached through discussion with a third senior member of the research team. A final 58 fulfilled all inclusion criteria and were included in our systematic review and 18 in meta‐analyses. As some studies included in the systematic review did not provide sufficient or appropriate data (e.g., reporting median and range instead of mean and standard deviation [SD] for plasma concentrations, or lacking subgroup data), we contacted the authors to request the required information for inclusion in the meta‐analysis. The authors of the four studies^40^, ^42^, ^43^, ^44^ indicated overlapping participants between two pairs of studies. To avoid duplication of data, we excluded the study with fewer participants^42^, ^44^ and retained the one with the highest number of participants,^40^, ^43^ ensuring a valid analysis. Furthermore, we were unable to get the necessary data from six studies^45^, ^46^, ^47^, ^48^, ^49^, ^50^ that otherwise fulfilled the criteria for inclusion, resulting in their removal from the meta‐analysis. We extracted data from each included study using a standardized form (Tables S1, S2 and S5 in supporting information).

### 2.4 Statistical analysis

All meta‐analyses were conducted using Review Manager (RevMan) version 5.4.^51^ The Aβ42/40 ratio was calculated using the Delta statistical method implemented in R.^52^, ^53^ Given that our data are sourced from diverse cohorts across different countries, we used a random effects model to calculate standardized mean differences (SMDs) to obtain effect sizes and 95% confidence intervals (CIs). The comparisons included DS individuals versus euploid controls, DS individuals with and without AD, and DS clinical subgroups (CS, pAD, and AD) regarding plasma levels of the following peptides: Aβ42, Aβ40, Aβ42/40 ratio, t‐tau, p‐tau 181, NfL, GFAP.

Quality assessment for eligible studies in the meta‐analysis was conducted using the Newcastle–Ottawa Scale (NOS).^53^, ^54^ Publication bias was assessed by visual inspection of the funnel plot. The statistical heterogeneity between studies was measured using *I* ^2^ tests.

In this analysis, we followed the AT(N) framework^14^, ^15^ to systematically present findings for each biomarker. Recent protocols^15^ have designated p‐tau181 and p‐tau217 as core tau (T) biomarkers. Although t‐tau has traditionally been categorized in the neurodegeneration (N) category due to its non‐specificity regarding AD pathology,^14^ recent protocols^15^ have introduced uncertainty about its classification. N‐terminal tau fragment (NT1‐tau) and phosphorylated tau at serine 396 (p‐S396) also required further validation as belonging in the T category. Given that this review explored the potential application of blood‐based AT(N) biomarkers for AD diagnosis in DS, we have provisionally included these three tau biomarkers (t‐tau, NT1‐tau, p‐S396) in the T category as supplementary, exploratory markers, with the understanding that further research is necessary.

## 3 RESULTS

We systematically reviewed 58 studies (Tables S1, S5) on BBM in DS, which together used seven different blood assay techniques including SIMOA, immunomagnetic reduction (IMR), electrochemiluminescence (ECL), ELISA, western blotting, solid phase immobilized epitope immunoassay, immunoprecipitation mass spectrometry (IP‐MS), Luminex system, reversed‐phase high‐performance liquid chromatography (RP‐HPLC), and latex‐enhanced nephelometry (LEN). The studies were conducted in multiple countries, including the United States, the United Kingdom, Spain, Japan, Germany, and France. Most research primarily examined plasma biomarkers, whereas only seven studies included serum biomarkers, and one study focused exclusively on neuronal exosomes.

Using the AT(N) framework, 20 studies examined amyloid biomarkers, including Aβ42, Aβ40, and Aβ42/40; 28 studies concentrated on tau (T) biomarkers, including t‐tau, p‐tau181, p‐tau217, NT1‐tau, and p‐S396; 28 studies investigated neurodegeneration (N) biomarkers, including NfL; and 30 studies explored non‐AT(N) biomarkers, which included inflammation markers such as astrocytes (GFAP), microglial (triggering receptor expressed on myeloid cells 2 [TREM2], soluble TREM2 [sTREM2]), mediator (high mobility group box 1 [HMGB1], matrix metalloproteinase‐3 [MMP‐3], and tissue plasminogen activator [tPA]), and cytokines including interleukin (IL)‐6, IL‐10, tumor necrosis factor‐alpha (TNFα), transforming growth factor‐beta 1 (TGF‐β1), IL‐2, IL‐1 alpha (IL‐1α), IL‐12, monocyte chemoattractant protein‐1 (MCP‐1), IL‐1 receptor antagonist (IL‐1ra), granulocyte‐macrophage colony‐stimulating factor (GM‐CSF), IL‐1 beta (IL‐1β), and erythropoietin (Epo). Additionally, the complement system biomarkers explored in the studies include activation products such as terminal complement complex (TCC) and inactivated complement component 3b (iC3b), along with complement proteins like complement component 1q (C1q), complement component 9 (C9), and complement component 3 (C3). The complement regulators involved in these studies were C1 inhibitor, factor H, factor H‐related protein 4 (FHR4), clusterin, factor I, and soluble complement receptor 1 (sCR1). Other biomarkers explored include dual‐specificity tyrosine‐phosphorylation‐regulated kinase 1A (DYRK1A) in both its full‐length and truncated forms, and activity‐dependent neuroprotective protein (ADNP). Neurotrophic factors like nerve growth factor (NGF) and its precursor proNGF were also studied. Beta‐synuclein was investigated as part of the synuclein family. In addition, neurotrophic factors such as brain‐derived neurotrophic factor (BDNF), acute phase proteins including C‐reactive protein (CRP) and serum amyloid A (SAA), growth factors like vascular endothelial growth factor (VEGF), APP processing markers including amyloid precursor‐like protein 1 (APL1) beta 25 (APL1β25), APL1β27, and APL1β28, as well as neurotransmitter metabolism markers like 3‐methoxy‐4‐hydroxyphenylglycol (MHPG), were examined.

In addition, 36 studies directly evaluated the BBMs’ significance in detecting AD and comparing DS persons to euploid control groups. Twenty‐two studies focused on BBM within individuals with DS without controls, or without comparing those with and without AD; instead, these investigated relationships with various parameters, including age,^48^, ^55^, ^56^ sex,^40^ genetics,^42^, ^55^ magnetic resonance imaging (MRI),^41^, ^47^, ^49^, ^50^, ^57^, ^58^ PET,^57^, ^59^ semantic verbal fluency,^43^ cognitive functions,^48^, ^57^, ^60^ metabolomics,^61^ markers of cerebrovascular diseases,^62^ proteomic profiles,^63^, ^64^, ^65^ cortical microinfarcts,^45^ functionalized liposomes,^66^ cognitive and developmental delays,^67^ and acute regression.^68^ Of these, we were able to obtain data from nine studies^8^, ^39^, ^40^, ^41^, ^43^, ^59^, ^69^, ^70^, ^71^ for inclusion in the meta‐analysis because the authors provided us with the means and SDs of plasma biomarker levels, as well as the number of participants within each clinical subgroup.

We were therefore able to conduct a meta‐analysis on data from 18 studies^8^, ^16^, ^39^, ^40^, ^41^, ^43^, ^59^, ^60^, ^69^, ^70^, ^71^, ^72^, ^73^, ^74^, ^75^, ^76^, ^77^, ^78^ (Table S2), all of which were considered high quality according to a thorough quality assessment. This meta‐analysis comprises 6 reports on amyloid, 12 studies on tau, 15 studies on neurodegeneration, and three studies on non‐AT(N) biomarkers. The meta‐analysis included a total of 3844 adult participants who were above the age of 18. Comparing the DS group to euploid controls, 1006 individuals were classified as the euploid control group, whereas 2109 individuals were in the DS group.  Comparing DS people with and without AD, 642 individuals were classified as having AD, while 2313 were categorized as without AD. According to DS clinical subgroups, 2320 were deemed to be CS, 443 were assessed to have pAD, and 590 were diagnosed with AD. Additional information on the age and sex of included participants per study is listed in Table S2.

### 3.1 Amyloid (A) biomarkers

#### 3.1.1 A ${\;\beta }_{42}$

##### Results of systematic review

Compared to the euploid controls, the majority of studies^8^, ^39^, ^71^, ^75^, ^79^, ^80^ consistently demonstrated that plasma Aβ42 concentrations were significantly increased in individuals with DS, which was supported by the study involving neuronal exosomes^81^ (Table S1). A cross‐sectional study revealed that, when categorizing the DS population into AD clinical groups (CS, pAD, and AD), each DS subgroup showed significantly higher Aβ42 levels than the euploid control group.^39^ However, a study using IMR technology to measure plasma Aβ42 concentrations, while supporting significantly elevated plasma Aβ42 levels in the DS without AD group, reported significantly lower levels in the overall DS group compared to euploid controls.^82^ In contrast, another study using the same IMR technology found the opposite result, with plasma Aβ42 levels significantly decreased in the DS without AD group, compared to euploid controls.^83^

Conflicting results were observed when comparing groups by AD status. Some studies indicated a significant elevation in plasma Aβ42 levels in DS individuals with AD compared to those without AD.^82^, ^83^ Nonetheless, other studies revealed no significant differences in plasma Aβ42 concentrations between the two groups.^8^, ^39^ Moreover, when categorizing the DS population into clinical subgroups (DS‐pAD vs. DS‐CS, DS‐AD vs. DS‐pAD), no significant differences were detected.^8^, ^39^

##### Results of meta‐analysis

Our meta‐analysis (Table 1) for DS versus euploid controls across five studies involving 676 DS individuals and 291 euploid controls found an SMD of 1.82 (95% CI [1.49, 2.15], *z* = 10.71, *p* < 0.0001), indicating significantly higher plasma Aβ42 levels in individuals with DS with substantial heterogeneity (*I* ^2^ = 72%, *p* = 0.006).

**TABLE 1:** Meta‐analysis of studies measuring plasma amyloid (A) biomarkers in DS individuals.

Plasma biomarkers						Heterogeneity			
	Groups	Studies	*N*	SMD (95% CI)	*z* value/*p* value	$Tau^{2}$	$Chi^{2}$	df	$I^{2}$
Aβ42	DS/NC	5	676/291	1.82 (1.49, 2.15)	10.71 (*p* < 0.0001)	0.10	14.39	4 (*p* = 0.006)	72%
	AD/NAD	4	155/619	0.18 (0.01, 0.36)	2.03 (*p* = 0.04)	0.00	0.02	3 (*p* = 1.00)	0%
	pAD/CS	3	109/595	−0.00 (−0.22, 0.22)	0.02 (*p* = 0.98)	0.00	2.31	2 (*p* = 0.32)	13%
	AD/pAD	3	148/109	0.18 (−0.07, 0.43)	1.41 (*p* = 0.16)	0.00	1.14	2 (*p* = 0.56)	0%
Aβ40	DS/NC	3	405/122	2.33 (1.84, 2.83)	9.30 (*p* < 0.00001)	0.13	6.35	2 (*p* = 0.04)	69%
	AD/NAD	3	83/405	0.34 (−0.23, 0.90)	1.17 (*p* = 0.24)	0.18	8.64	2 (*p* = 0.01)	77%
	pAD/CS	2	72/381	0.30 (0.04, 0.55)	2.30 (*p* = 0.02)	0.00	0.35	1 (*p* = 0.56)	0%
	AD/pAD	2	76/72	0.02 (−0.75, 0.78)	0.04 (*p* = 0.97)	0.25	5.31	1 (*p* = 0.02)	81%
A $\;\beta \frac{42}{40}$	DS/NC	3	405/122	−0.56 (−1.01, −0.12)	2.47 (*p* = 0.01)	0.11	7.19	2 (*p* = 0.03)	72%
	AD/NAD	3	83/405	−0.13 (−0.50, 0.25)	0.67 (*p* = 0.50)	0.05	3.93	2 (*p* = 0.14)	49%
	pAD/CS	3	113/600	−0.22 (−0.42, −0.01)	2.11 (*p* = 0.04)	0.00	0.35	2 (*p* = 0.84)	0%
	AD/pAD	2	76/72	0.10 (−0.22, 0.43)	0.63 (*p* = 0.53)	0.00	0.55	1 (*p* = 0.46)	0%

Abbreviations: Aβ, amyloid beta; AD, Alzheimer's disease; CI, confidence Interval; DS, Down syndrome; DS_AD, Down syndrome with Alzheimer's disease; DS_CS, Down syndrome with cognitively stable; DS_NAD, Down syndrome without Alzheimer's disease; DS_pAD, Down syndrome with prodromal Alzheimer's disease; NC, normal controls; pAD, prodromal Alzheimer's disease; SMD, standardized mean difference.

For the comparison of plasma Aβ42 levels between DS with and without AD groups, including 155 with AD and 619 without AD individuals from four studies, the SMD was 0.18 (95% CI [0.01, 0.36], *z* = 2.03, *p* = 0.04), showing a smaller but significant difference with no heterogeneity (*I* ^2^ = 0%, *p* = 1.00). In contrast, comparisons between DS with pAD and DS with CS groups, based on 109 pAD and 595 CS individuals from three studies, found no significant difference in plasma Aβ42 levels (SMD = −0.00, 95% CI [−0.22, 0.22], *z* = 0.02, *p* = 0.98), with low heterogeneity (*I* ^2^ = 13%, *p* = 0.32). Finally, for DS with AD versus DS with pAD, across three studies with 148 AD and 109 pAD individuals, the SMD was 0.18 (95% CI [−0.07, 0.43], *z* = 1.41, *p* = 0.16), showing no significant difference in plasma Aβ42 levels and no heterogeneity (*I* ^2^ = 0%, *p* = 0.56).

##### Associations with other biomarkers and clinical factors

Studies have found that high plasma Aβ42 concentrations existed in newborns with DS, and individuals with and without DS experience a decrease in Aβ42 levels with age.^48^, ^75^ The reduction was greater in the DS group, being especially apparent by ≈ 30 years of age.^75^ However, several studies have indicated that Aβ42 levels are not age dependent.^71^, ^79^ The plasma Aβ40 and Aβ42 levels were negatively associated with the Gesell Developmental Schedules scores in children with DS.^67^ In addition, no association was identified between plasma and CSF Aβ42 concentrations in the DS group;^39^ however, a moderate positive correlation was observed between plasma Aβ42 and t‐tau levels.^71^ Moreover, composite biomarker scores, including both plasma tau and Aβ42 levels, demonstrated a more robust correlation with dementia in AD than scores derived from a single biomarker.^83^

#### 3.1.2 A $\beta_{40}$

##### Results of systematic review

Previous studies consistently found that plasma Aβ40 concentrations were significantly elevated in individuals with DS compared to euploid controls.^39^, ^71^, ^80^, ^82^ Furthermore, when the DS population was clinically classified for AD, comparisons between each clinical subgroup and the euploid control group also yielded similar conclusions.^39^, ^83^ However, one study that used IMR technology reported contradictory findings. Although the DS group had higher plasma Aβ40 concentrations overall, the study found that the DS without AD group had significantly lower plasma Aβ40 levels compared to the euploid control group.^82^

When the DS population was divided into DS with AD and DS without AD groups based on clinical AD diagnoses, inconsistent findings emerged. Some studies reported that plasma Aβ40 levels were significantly higher in the DS with AD group compared to the DS without AD group,^39^ while others observed a significant decrease.^82^, ^83^ However, when further stratifying the DS population into CS, pAD, and AD subgroups, no significant differences in plasma Aβ40 levels were found between these subgroups.^39^

##### Results of meta‐analysis

In our meta‐analysis (Table 1), the comparison of plasma Aβ40 levels between DS individuals and euploid controls, based on three studies involving 405 DS individuals and 122 euploid controls, revealed a significantly higher plasma Aβ40 concentration in the DS group, with an SMD of 2.33 (95% CI [1.84, 2.83], *z* = 9.30, *p* < 0.00001). However, there was substantial heterogeneity (*I* ^2^ = 69%, *p* = 0.04).

For the comparison between DS with and without AD groups, across three studies involving 83 DS with AD individuals and 405 DS without AD, the analysis showed no significant difference in plasma Aβ40 levels, with an SMD of 0.34 (95% CI [−0.23, 0.90], *z* = 1.17, *p* = 0.24), and high heterogeneity (*I* ^2^ = 77%, *p* = 0.01). However, the comparison between DS‐pAD and DS‐CS groups, from two studies involving 72 DS‐pAD individuals and 381 DS‐CS, showed a small but significant increase in plasma Aβ40 levels in the pAD group (SMD = 0.30, 95% CI [0.04, 0.55], *z* = 2.30, *p* = 0.02), with no heterogeneity (*I* ^2^ = 0%, *p* = 0.56). Finally, in the comparison between DS with AD and DS with pAD groups, across two studies involving 76 DS‐AD individuals and 72 DS‐pAD individuals, the analysis found no significant difference in plasma Aβ40 levels (SMD = 0.02, 95% CI [−0.75, 0.78], *z* = 0.04, *p* = 0.97), with substantial heterogeneity (*I* ^2^ = 81%, *p* = 0.02).

##### Associations with other biomarkers and clinical factors

When exploring the relationship between plasma Aβ40 and other markers, studies found no significant correlation between plasma Aβ40 levels and age,^71^ nor with CSF Aβ40 concentrations.^39^

#### 3.1.3 A $\beta_{42/40}$ ratio

##### Results of systematic review

Previous studies consistently demonstrated that the plasma Aβ42/40 ratio decreased significantly in persons with DS compared to euploid controls.^71^, ^82^ When the DS population was clinically classified for AD and non‐AD, the study indicated that the plasma Aβ42/40 ratio was markedly elevated in the DS without AD group relative to euploid controls, but the DS with AD group displayed a higher ratio compared to the DS without AD group.^82^

##### Results of meta‐analysis

In our meta‐analysis (Table 1), for DS versus euploid controls, based on three studies with 405 DS individuals and 122 euploid controls, the meta‐analysis showed an SMD of −0.56 (95% CI [−1.01, −0.12], *z* = 2.47, *p* = 0.01), indicating that the Aβ42/40 ratio was significantly lower in the DS group. There was substantial heterogeneity (*I* ^2^ = 72%, *p* = 0.03). Also, for DS with AD versus DS without AD, across three studies involving 83 with AD and 405 without AD individuals, the SMD was −0.13 (95% CI [−0.50, 0.25], *z* = 0.67, *p* = 0.50), showing no significant difference in the Aβ42/40 ratio, with moderate heterogeneity (*I* ^2^ = 49%, *p* = 0.14). However, in the comparison between DS‐pAD and DS‐CS groups, across three studies with 113 DS‐pAD and 600 DS‐CS individuals, the SMD was −0.22 (95% CI [−0.42, −0.01], *z* = 2.11, *p* = 0.04), indicating a small but significant decrease in the Aβ42/40 ratio for the DS‐pAD group. There was no heterogeneity (*I* ^2^ = 0%, *p* = 0.84). Finally, for the comparison between DS with AD and DS with pAD, across two studies involving 76 DS‐AD and 72 DS‐pAD individuals, the SMD was 0.10 (95% CI [−0.22, 0.43], *z* = 0.63, *p* = 0.53), showing no significant difference in the Aβ42/40 ratio, with no heterogeneity (*I* ^2^ = 0%, *p* = 0.46).

##### Associations with other biomarkers and clinical factors

In individuals with DS, changes in the plasma Aβ42/40 ratio occurred early, initially increasing and then declining around the age of 45.^56^ Besides, the plasma Aβ42/40 ratio was positively correlated with visuospatial ability,^60^ while showing a negative correlation with both NfL and p‐tau181 levels.^56^

### 3.2 Tau (T) biomarkers

#### 3.2.1 Non‐phosphorylated tau

##### Results of systematic review

The published studies on tau biomarkers in DS predominantly focused on t‐tau, p‐tau181, p‐tau217, NT1‐tau, and P‐S396‐tau (Table S1). The systematic review found mixed results when comparing plasma t‐tau levels between DS individuals and general population euploid controls. Some studies reported a significant increase in plasma t‐tau levels in the DS population,^46^, ^72^, ^82^ while others found no significant differences.^8^, ^71^ When the DS population was clinically classified for AD diagnosis, conflicting findings also emerged: some studies reported significantly higher plasma t‐tau levels in the DS with AD group compared to the DS without AD group,^39^, ^78^ while others found either reduced levels^83^ or no significant difference.^82^ Furthermore, when additional clinical subgroup categorizing was conducted, studies observed significant differences in plasma t‐tau levels between the DS with AD group and both the euploid controls^39^ and the pAD group.^78^ Also, no significant differences have been found between euploid controls versus DS‐pAD and CS, DS‐pAD versus DS‐CS and AD.^39^ Nevertheless, one study indicated that plasma t‐tau levels were increased more in DS without AD than in euploid controls.^83^

##### Results of meta‐analysis

In our meta‐analysis (Table 2), for DS versus euploid controls, based on five studies involving 637 DS individuals and 249 euploid controls, the SMD was 0.04 (95% CI [−0.25, 0.32], *z* = 0.25, *p* = 0.80), indicating no significant difference in plasma t‐tau levels between the DS group and euploid controls. There was moderate heterogeneity (*I* ^2^ = 64%, *p* = 0.03). In contrast, for the comparison between DS with and without AD, across five studies involving 191 DS with AD individuals and 842 without AD individuals, the SMD was 0.35 (95% CI [0.12, 0.57], *z* = 3.02, *p* = 0.003), showing a significant increase in plasma t‐tau levels in the DS with AD group, with moderate heterogeneity (*I* ^2^ = 44%, *p* = 0.13). In addition, in the comparison between DS‐pAD and DS‐CS groups, across five studies with 194 DS‐pAD and 1037 DS‐CS individuals, the SMD was 0.22 (95% CI [−0.05, 0.48], *z* = 1.61, *p* = 0.11), indicating no significant difference in plasma t‐tau levels, with substantial heterogeneity (*I* ^2^ = 65%, *p* = 0.02). Finally, for the comparison between DS‐AD and DS‐pAD, across four studies involving 184 DS‐AD and 153 DS‐pAD individuals, the SMD was 0.52 (95% CI [0.30, 0.74], *z* = 4.59, *p* < 0.00001), indicating a significantly higher plasma t‐tau level in the DS with AD group. There was no heterogeneity (*I* ^2^ = 0%, *p* = 0.84).

**TABLE 2:** Meta‐analysis of studies measuring plasma tau (T) biomarkers in DS individuals.

Plasma biomarkers						Heterogeneity			
	Groups	Studies	*N*	SMD (95% CI)	*z* value/ *p* value	$Tau^{2}$	$Chi^{2}$	Df	$I^{2}$
t‐tau	DS/NC	5	637/249	0.04 (−0.25, 0.32)	0.25 (*p* = 0.80)	0.06	11.13	4 (*p* = 0.03)	64%
	AD/NAD	5	191/842	0.35 (0.12, 0.57)	3.02 (*p* = 0.003)	0.03	7.12	4 (*p* = 0.13)	44%
	pAD/CS	5	194/1037	0.22 (−0.05, 0.48)	1.61 (*p* = 0.11)	0.06	11.55	4 (*p* = 0.02)	65%
	AD/pAD	4	184/153	0.52 (0.30, 0.74)	4.59 (*p* < 0.00001)	0.00	0.86	3 (*p* = 0.84)	0%
p‐tau181	DS/NC	5	1110/503	0.07 (−0.08, 0.22)	0.89 (*p* = 0.38)	0.01	6.48	4 (*p* = 0.17)	38%
	AD/NAD	4	371/1090	1.33 (1.10, 1.57)	11.16 (*p* < 0.00001)	0.04	9.56	3 (*p* = 0.02)	69%
	pAD/CS	4	208/1090	0.90 (0.63, 1.18)	6.41 (*p* < 0.00001)	0.05	9.52	3 (*p* = 0.02)	68%
	AD/pAD	4	371/208	0.41 (0.24, 0.59)	4.70 (*p* < 0.00001)	0.00	2.19	3 (*p* = 0.53)	0%

Abbreviations: AD, Alzheimer's disease; CI, confidence Interval; DS, Down syndrome; DS_AD, Down syndrome with Alzheimer's disease; DS_CS, Down syndrome with cognitively stable; DS_NAD, Down syndrome without Alzheimer's disease; DS_pAD, Down syndrome with prodromal Alzheimer's disease; NC, normal controls; pAD, prodromal Alzheimer's disease; p‐tau181, phosphorylated tau 181; SMD, standardized mean difference; t‐tau, total tau protein.

##### Associations with other biomarkers and clinical factors

When considering the relationship between plasma t‐tau and other factors, many studies have focused on age, but the findings have been inconsistent. Some studies reported that plasma t‐tau levels in DS individuals increased in an age‐dependent manner,^72^ while others found no moderate or strong correlation with age.^71^ Also, for DS individuals with mild cognitive impairment (DS‐MCI), plasma t‐tau alone was found to have low diagnostic accuracy.^78^ However, when combined with NfL, age, and sex, the diagnostic accuracy increased to 87%, and it was found that both t‐tau and age were associated with an increased risk of MCI in DS individuals.^78^ In one study^59^ using amyloid and tau PET imaging, it was observed that plasma t‐tau levels in DS individuals with amyloid PET‐positive scans were significantly elevated compared to amyloid PET‐negative euploid controls, while no increase was seen in the amyloid PET‐negative DS group. Furthermore, compared to DS individuals who were both amyloid and tau PET negative, those with amyloid PET–positive but tau‐negative scans, as well as those with both amyloid and tau PET–positive scans, exhibited significantly higher plasma t‐tau levels. In addition, plasma t‐tau levels might be elevated in DS individuals with acute regression compared to unaffected DS individuals; however, no statistical analysis was conducted to confirm this.^68^ Plasma t‐tau in DS individuals was found to be negatively correlated with episodic memory performance,^60^ positively associated with connectivity in certain default mode network regions,^58^ and showed a weak correlation with CSF t‐tau levels.^39^

Previous research has found that plasma NT1‐tau concentrations are significantly higher in DS individuals compared to euploid controls.^75^ Plasma NT1‐tau levels were high in DS individuals during infancy, gradually decreased during adolescence, and increased again in older age.^75^ By contrast, one study has reported that plasma NT1‐tau levels in DS increase with age and are associated with cognitive decline.^48^

#### 3.2.2 p‐tau181

##### Results of systematic review

Previous studies on plasma p‐tau181 have largely reported consistent findings. One study compared plasma p‐tau181 levels between DS individuals and euploid controls and found a significant difference.^73^ This finding was further supported by a study on neuronal exosomes.^81^ When clinically categorizing the DS population for AD, studies consistently reported significant differences in plasma p‐tau181 levels between DS with and without AD groups.^70^, ^84^ Additionally, when further subdividing the DS population into clinical subgroups (CS, pAD, AD), significant differences were observed between all subgroups.^70^ Furthermore, one study comparing DS with AD to euploid controls found a significant increase in plasma p‐tau181 in DS‐AD,^84^ while no significant differences were observed between the DS with CS group and euploid controls.^70^

##### Results of meta‐analysis

In our meta‐analysis (Table 2), for DS versus euploid controls, based on five studies involving 1110 DS individuals and 503 euploid controls, the SMD was 0.07 (95% CI [−0.08, 0.22], *z* = 0.89, *p* = 0.38), indicating no significant difference in plasma p‐tau181 levels between the DS group and euploid controls. There was moderate heterogeneity (*I* ^2^ = 38%, *p* = 0.17). However, for the comparison between DS with and without AD, across four studies involving 371 DS with AD individuals and 1090 without AD individuals, the SMD was 1.33 (95% CI [1.10, 1.57], *z* = 11.16, *p* < 0.00001), showing a significant increase in plasma p‐tau181 levels in the DS with AD group, with substantial heterogeneity (*I* ^2^ = 69%, *p* = 0.02). Also, in the comparison between DS‐pAD and DS‐CS groups, across four studies with 208 DS‐pAD and 1090 DS‐CS individuals, the SMD was 0.90 (95% CI [0.63, 1.18], *z* = 6.41, *p* < 0.00001), indicating a significant increase in plasma p‐tau181 levels in the DS‐pAD group. There was substantial heterogeneity (*I* ^2^ = 68%, *p* = 0.02). Finally, for the comparison between DS‐AD and DS‐pAD, across four studies involving 371 DS‐AD and 208 DS‐pAD individuals, the SMD was 0.41 (95% CI [0.24, 0.59], *z* = 4.70, *p* < 0.00001), showing significantly higher plasma p‐tau181 levels in the DS with AD group, with no heterogeneity (*I* ^2^ = 0%, *p* = 0.53).

##### Associations with other biomarkers and clinical factors

When considering the relationship between plasma p‐tau181 and other markers, studies have found a significant positive correlation between plasma p‐tau181 levels and age in DS individuals,^55^, ^73^ with a marked increase around the age of 40.^56^ However, no significant correlation was observed between p‐tau181 and sex, although both CSF and plasma p‐tau181 levels in female DS individuals tended to increase between the ages of 40 and 50.^40^ Further analysis of the apolipoprotein E (*APOE*) ε4 genotype in the DS population revealed that carriers of the *APOE* ε4 allele had lower CSF Aβ1‐42 to Aβ1‐40 ratios before the age of 40, and showed earlier increases in amyloid PET uptake and plasma p‐tau181 levels, along with earlier cortical metabolic decline and hippocampal volume loss.^42^ Moreover, one amyloid PET study also found that DS participants with amyloid PET–positive scans had significantly higher plasma p‐tau181 levels compared to amyloid PET–negative individuals.^70^ In addition, plasma p‐tau181 levels were significantly associated with the volume of the basal forebrain, plasma NfL levels, the CSF Aβ1‐42 to Aβ1‐40 ratio, CSF t‐tau, CSF p‐tau181, CSF NfL concentrations, gray matter volume loss, and the atrophy of characteristic AD regions measured by MRI, including the temporal angular gyrus, supramarginal gyrus, and precuneus in both hemispheres.^41^, ^49^, ^70^

#### 3.2.3 p‐tau217

##### Results of systematic review

There is limited research on plasma p‐tau217 in the DS population, and no studies have directly analyzed differences in plasma p‐tau217 levels among clinical AD diagnosis groups. As a result, we did not have enough studies to conduct a meta‐analysis. Previous research has found a strong correlation between p‐tau217 and p‐tau181 in DS individuals, with plasma p‐tau217 levels gradually increasing and showing a marked rise around the age of 40.^56^ One study^59^ using amyloid and tau PET imaging found that, compared to DS individuals who were both amyloid and tau PET negative, plasma p‐tau217 levels were significantly elevated in those who were amyloid PET positive but tau PET negative, and in those who were both amyloid PET positive and tau PET positive. Furthermore, among amyloid PET–positive DS individuals, plasma p‐tau217 levels were significantly higher in those who were tau PET positive compared to those who were tau PET negative. When comparing DS individuals with amyloid PET–negative non‐DS siblings, plasma p‐tau217 levels increased in amyloid PET–positive DS individuals but not in amyloid PET–negative DS individuals. These findings indicated that plasma p‐tau217 can accurately identify individuals with abnormal tau PET and Aβ PET scans, especially when considered in conjunction with age.^59^ Additionally, higher plasma p‐tau217 levels were consistently associated with NfL^50^ and poorer cognitive test performance.^59^

Moreover, a study on neuronal exosomes found that p‐s396‐tau concentrations were significantly higher in DS individuals compared to euploid controls.^81^

### 3.3 Neurodegeneration (N) biomarkers

#### 3.3.1 Results of systematic review

The systematic review found that research on neurodegeneration biomarkers has primarily focused on plasma NfL, with all studies using the SIMOA technique for blood detection. Most studies consistently reported that plasma NfL levels were significantly elevated in DS individuals compared to normal euploid controls.^39^, ^46^, ^70^, ^75^, ^77^ One study, which clinically stratified the DS population into CS, pAD, and AD subgroups, found significant differences between each DS subgroup and euploid controls.^39^ However, another study reported that plasma NfL was only significantly elevated in the DS with AD group compared to euploid controls and did not observe significant increases across the entire DS group.^16^

Previous studies consistently reported significant differences in plasma NfL levels between DS with and without AD, as well as between DS‐pAD and CS‐DS.^16^, ^39^, ^44^, ^69^, ^74^, ^78^ However, when comparing DS‐AD to pAD, findings have been more mixed, with some studies reporting no significant difference,^39^, ^74^ while others observed a significant increase in plasma NfL levels.^78^

#### 3.3.2 Results of meta‐analysis

In our meta‐analysis (Table 3), for DS versus euploid controls, based on 10 studies involving 1741 DS individuals and 896 euploid controls, the SMD was 0.49 (95% CI [0.23, 0.75], *z* = 3.64, *p* = 0.0003), indicating significantly higher plasma NfL levels in the DS group. There was substantial heterogeneity (*I* ^2^ = 88%, *p* < 0.00001).

**TABLE 3:** Meta‐analysis of studies measuring plasma neurodegeneration (N) biomarkers in DS individuals.

Plasma biomarkers						Heterogeneity			
	Groups	Studies	N	SMD (95% CI)	*z* value/ *p* value	$Tau^{2}$	$Chi^{2}$	df	$I^{2}$
NfL	DS/NC	10	1741/896	0.49 (0.23, 0.75)	3.64 (*p* = 0.0003)	0.15	72.24	9 (*p* < 0.00001)	88%
	AD/NAD	12	594/2210	1.88 (1.73, 2.03)	25.09 (*p* < 0.00001)	0.03	19.55	11 (*p* = 0.05)	44%
	pAD/CS	10	420/2241	1.15 (0.96, 1.35)	11.56 (*p* < 0.00001)	0.07	28.27	9 (*p* = 0.0009)	68%
	AD/pAD	9	549/379	0.62 (0.49, 0.76)	8.96 (*p* < 0.00001)	0.00	4.30	8 (*p* = 0.83)	0%

Abbreviations: AD, Alzheimer's disease; CI, confidence Interval; DS, Down syndrome; DS_AD, Down syndrome with Alzheimer's disease; DS_CS, Down syndrome with cognitively stable; DS_NAD, Down syndrome without Alzheimer's disease; DS_pAD, Down syndrome with prodromal Alzheimer's disease; NC, normal controls; NfL, neurofilament light chain; pAD, prodromal Alzheimer's disease; SMD, standardized mean difference.

In the comparison between DS with and without AD, across 12 studies involving 594 DS with AD and 2210 without AD individuals, the SMD was 1.88 (95% CI [1.73, 2.03], *z* = 25.09, *p* < 0.00001), showing a significantly higher plasma NfL concentration in the DS with AD group, with moderate heterogeneity (*I* ^2^ = 44%, *p* = 0.05). Also, for DS‐pAD versus DS‐CS, across 10 studies involving 420 DS‐pAD and 2241 DS‐CS individuals, the SMD was 1.15 (95% CI [0.96, 1.35], *z* = 11.56, *p* < 0.00001), with substantial heterogeneity (*I* ^2^ = 68%, *p* = 0.0009). Finally, in the comparison between DS‐AD and DS‐pAD, across 9 studies involving 549 DS‐AD and 379 DS‐pAD individuals, the SMD was 0.62 (95% CI [0.49, 0.76], *z* = 8.96, *p* < 0.00001), indicating significantly higher plasma NfL levels in the DS with AD group, with no heterogeneity (*I* ^2^ = 0%, *p* = 0.83).

#### 3.3.3 Associations with other biomarkers and clinical factors

When considering the relationship between plasma NfL and other markers, studies have shown that plasma NfL levels were relatively low in infants^75^ but increased with age,^40^, ^44^, ^69^, ^71^, ^74^, ^77^ with a significant rise occurring around the ages of 30^8^ or 40.^69^ In DS individuals, plasma NfL levels were significantly correlated with CSF NfL,^39^ basal forebrain volumes,^41^ longitudinal dementia status,^69^ gray matter volume loss,^49^ amyloid pathology and hippocampal atrophy,^57^ white matter hyperintensity volume,^50^ plasma p‐tau217, p‐tau181, and GFAP.^50^, ^56^ However, no significant relationship has been found with the *APOE* ε4 allele,^42^ long‐term epilepsy, or premorbid abilities.^69^ Plasma NfL levels were 14.8% lower in male DS participants compared to females.^74^ Due to the small sample size, statistical analysis was not conducted, but elevated plasma NfL levels suggested that DS individuals with acute regression may be at higher risk for early‐onset AD.^68^ Plasma NfL levels were significantly higher in DS participants with cortical microinfarcts compared to those without.^45^

An imaging study revealed that plasma NfL levels were significantly elevated in both amyloid PET–positive and PET‐negative DS individuals compared to amyloid PET–negative euploid controls.^59^ Additionally, plasma NfL levels were associated with hippocampal volume and the thickness of the left anterior‐lateral entorhinal cortex (alEC), and mediated the relationship between left alEC thickness and memory, as well as between hippocampal volume and memory.^47^ Plasma NfL levels were negatively correlated with the number of correct words in verbal fluency tasks, with age (primarily driven by older individuals),^43^ and positively correlated with errors in the Cambridge Neuropsychological Test Automated Battery paired associate learning (PAL) tests.^57^ They were also negatively associated with visuospatial abilities and episodic memory.^60^

Plasma NfL levels alone achieved high accuracy in distinguishing DS with AD participants from DS with CS individuals, and when combined with total tau, age, and sex, the accuracy increased to 93%.^78^ For DS with MCI participants, the accuracy of using plasma NfL alone was lower, but combining NfL, t‐tau, age, and sex improved the accuracy to 87%.^78^ Furthermore, neurodegenerative protein concentrations were associated with markers of cerebrovascular disease, particularly in individuals exhibiting AD symptoms.^62^

### 3.4 Non‐AT(N) biomarkers

There has been an increasing interest in investigating non‐AT(N) blood biomarkers in recent years,^71^, ^85^, ^86^, ^87^, ^88^, ^89^ using both plasma and serum and various techniques (SIMOA, ELISA, CL, solid phase immobilized epitope immunoassay, and LEN; see Table S1). Due to the limited number of studies for each biomarker, we conducted the meta‐analysis only for plasma GFAP.

#### 3.4.1 Inflammation biomarkers

##### Results of systematic review—GFAP

The study found that plasma GFAP levels were significantly elevated in the DS with AD group compared to the DS without AD group.^76^ Also, significant differences in plasma GFAP levels were observed between DS‐pAD and CS individuals.^76^

##### Results of meta‐analysis—GFAP

In our meta‐analysis of plasma GFAP (Table 4), for DS versus euploid controls, based on two studies involving 599 DS individuals and 235 euploid controls (without DS), the SMD was 0.54 (95% CI [0.27, 0.80], *z* = 3.98, *p* < 0.0001), indicating a significant increase in plasma GFAP levels in the DS group compared to euploid controls, with moderate heterogeneity (*I* ^2^ = 50%, *p* = 0.16). Also, in the comparison between DS with and without AD, across three studies involving 172 DS with AD individuals and 658 without AD individuals, the SMD was 1.98 (95% CI [1.78, 2.18], *z* = 19.86, *p* < 0.00001), showing a significant increase in plasma GFAP levels in the DS with AD group, with no heterogeneity (*I* ^2^ = 0%, *p* = 0.89). In addition, for DS‐pAD versus DS‐CS individuals, based on two studies involving 109 DS‐pAD and 599 DS‐CS individuals, the SMD was 1.57 (95% CI [0.91, 2.23], *z* = 4.66, *p* < 0.00001), showing significant differences, though with substantial heterogeneity (*I* ^2^ = 88%, *p* = 0.004). Finally, in the comparison between DS‐AD and DS‐pAD, based on two studies involving 162 DS‐AD and 109 DS‐pAD individuals, the SMD was 0.42 (95% CI [0.17, 0.67], *z* = 3.29, P*p* = 0.001), indicating a significant difference in plasma GFAP levels between the two groups, with no heterogeneity (*I* ^2^ = 0%, *p* = 0.77).

**TABLE 4:** Meta‐analysis of studies measuring plasma inflammation (non‐AT[N]) biomarkers in DS individuals.

Plasma biomarkers						Heterogeneity			
	Groups	Studies	N	SMD (95% CI)	*z* value/ *p* value	$Tau^{2}$	$Chi^{2}$	df	$I^{2}$
GFAP	DS/NC	2	599/235	0.54 (0.27, 0.80)	3.98 (*p* < 0.0001)	0.02	1.99	1 (*p* = 0.16)	50%
	AD/NAD	3	172/658	1.98 (1.78, 2.18)	19.86 (*p* < 0.00001)	0.00	0.23	2 (*p* = 0.89)	0%
	pAD/CS	2	109/599	1.57 (0.91, 2.23)	4.66 (*p* < 0.00001)	0.20	8.42	2 (*p* = 0.004)	88%
	AD/pAD	2	162/109	0.42 (0.17, 0.67)	3.29 (*p* = 0.001)	0.00	0.08	1 (*p* = 0.77)	0%

Abbreviations: AD, Alzheimer's disease; CI, confidence Interval; DS, Down syndrome; DS_AD, Down syndrome with Alzheimer's disease; DS_CS, Down syndrome with cognitively stable; DS_NAD, Down syndrome without Alzheimer's disease; DS_pAD, Down syndrome with prodromal Alzheimer's disease; GFAP, glial fibrillary acidic protein; NC, normal controls; pAD, prodromal Alzheimer's disease; SMD, standardized mean difference.

##### Associations with other biomarkers and clinical factors

Plasma GFAP levels have been found to increase in the late 20s, while reported CSF Aβ levels decrease.^76^ GFAP levels were also highly correlated with NfL, cortical thinning, and amyloid pathology in the brain.^50^, ^76^ Additionally, increased GFAP levels were associated with a lower number of words generated in verbal fluency tasks, with this relationship being primarily driven by older individuals.^43^ One study^59^ using amyloid and tau PET imaging found that compared to DS individuals who were both amyloid and tau PET negative, plasma GFAP levels were significantly elevated in those who were both amyloid PET positive and tau PET positive. Comparing DS individuals with amyloid PET–negative non‐DS siblings, plasma GFAP levels increased in both amyloid PET positive and negative DS individuals.

##### Results of systematic review—other inflammatory markers

In addition to plasma GFAP, several inflammatory proteins have yielded promising results. For example, mediators (HMFBI, MMP‐3, and tPA), microglial (sTREM2), as well as cytokines (IL‐6, IL‐10, TGF‐β1, IL‐2, IL‐1ra, GM‐CSF, Epo, IL‐1 β), showed significant differences in plasma between DS individuals and euploid controls, though some biomarkers (IL‐6, IL‐10, TNFα, TGF‐β1, IL‐2, IL‐1α, IL‐12, MCP‐1) in serum did not produce significant results or some like IL‐6 exhibited varying outcomes across studies.^71^, ^85^, ^86^, ^87^, ^88^, ^90^, ^91^, ^92^, ^93^, ^94^, ^95^ Inflammatory protein concentrations were also linked to cerebrovascular disease markers, particularly in individuals with AD symptoms.^62^ Proteomic profiles demonstrated excellent detection accuracy for identifying MCI‐DS and DS‐AD.^63^, ^64^, ^65^ Plasma sTREM2 levels were observed to decrease with age in DS,^55^, ^93^ and positively correlated with connectivity in some anterior default mode network regions.^58^ Both DS and sporadic AD (sAD) groups showed a moderate positive association between IL‐10 and TNFα, while euploid controls exhibited a moderate negative association between the Aβ42/Aβ40 ratio and IL‐10.^71^ Within the DS group, there were strong positive associations between IL‐1β and IL‐10, and moderate positive associations between IL‐6 and TNFα.^71^ IL‐1β levels in DS individuals were also moderately positively associated with t‐tau concentration and moderately negatively associated with the Aβ42/t‐tau ratio.^71^ Last, a negative correlation was found between low TGF‐β1 concentrations and high TNFα plasma levels.^86^

#### 3.4.2 Complement biomarkers

##### Results of systematic review

Activation products (TCC and iC3b), proteins (C1q, C9, and C3), and regulators (C1 inhibitor, factor H, FHR4, clusterin) were found to be elevated and other regulators like Factor I and sCR1 reduced in DS individuals compared to euploid controls.^85^, ^87^, ^90^ However, C3 and Factor I levels were decreased in DS with AD group compared to DS without AD group.^87^ In addition, neither the *APOE* genotype nor clusterin single nucleotide polymorphisms affected complement levels, but the rs6656401 variant in CR1 significantly impacted plasma sCR1 levels.^87^

#### 3.4.3 Other non‐AT(N) biomarkers

##### Results of systematic review

Nerve growth factor (proNGF), beta‐synuclein, neurotrophic factor (BDNF), acute phase proteins (CRP and SAA), and VEGF were elevated in DS individuals compared to euploid controls.^84^, ^85^, ^87^, ^88^, ^90^, ^91^, ^96^, ^97^ In contrast, the serum levels of APL1β25, APL1β27, APL1β28, and MHPG were significantly reduced in DS individuals than in euploid controls.^98^ Also, plasma DYRK1A (full‐length) levels and NGF levels decreased in DS with AD compared to DS without AD, while serum synuclein levels increased.^84^, ^90^, ^96^ Metabolomic analysis showed that DS individuals produce elevated levels of kynurenine and quinolinic acid, two tryptophan metabolites with immunosuppressive and neurotoxic effects.^61^

### 3.5 Quality assessment and sensitivity analysis

Quality assessment results indicated that all included studies achieved high methodological quality, as shown in Table S6 in supporting information. Sensitivity analyses, conducted by excluding studies with potential bias, confirmed that the main results remained stable. However, due to the limited number of studies in certain analyses, sensitivity analysis could not be performed in those cases. Detailed forest plots, funnel plots, and sensitivity analysis results are available in Figures S1–S33 in supporting information.

## 4 DISCUSSION

We conducted a systematic review and meta‐analysis to evaluate the potential utility of the AT(N) blood biomarker framework for diagnosing AD in individuals with DS, which might enhance and optimize future pharmacological clinical trials for AD and improve the early detection of AD in DS.

The meta‐analysis demonstrated that people with DS had significantly higher levels of plasma Aβ42, Aβ40, NfL, and GFAP compared to euploid controls, alongside a notable decrease in the plasma Aβ42/40 ratio. Comparing DS with AD to DS without AD, the concentrations of plasma Aβ42, t‐tau, p‐tau181, NfL, and GFAP were significantly elevated in the DS with AD group. Further clinical classification of the DS population revealed that compared to the DS‐CS group, plasma Aβ40, p‐tau181, NfL, and GFAP were significantly increased in the DS‐pAD group, while the plasma Aβ42/40 ratio was significantly decreased. However, there were no significant differences in amyloid biomarkers between DS individuals with AD and those with pAD, although the levels of plasma t‐tau, p‐tau181, NfL, and GFAP were markedly elevated in the DS‐AD group.

### 4.1 Amyloid

Significant differences in plasma Aβ40, Aβ42, and the Aβ42/40 ratio between people with DS and euploid control groups were consistently identified by the systematic review and meta‐analysis, which were also supported by previous meta‐analysis studies.^32^, ^99^ DS is due to triplication of chromosome 21, which causes the *APP* gene to overexpress and results in the anticipated 1.5‐fold rise in amyloid levels.^100^, ^101^ A study^75^ on DS individuals across all age groups suggested that DS infants exhibit higher plasma Aβ42 levels compared to euploid controls, but these levels decline with age. Moreover, the reduction in plasma Aβ42 levels is more pronounced in the DS group than in euploid controls, with the decline plateauing around the age of 30. Although the majority of studies, as well as our meta‐analysis, consistently found that plasma Aβ40 and Aβ42 levels were significantly elevated and the Aβ42/40 ratio significantly decreased in DS individuals compared to euploid controls, some studies reported inconsistent findings.^82^, ^83^ These discrepancies may be attributed to differences in plasma detection methods. In our meta‐analysis, we only included studies that used the SIMOA technology to measure plasma concentrations.

In addition, after clinically categorizing the DS population based on AD diagnoses, our meta‐analysis found that plasma Aβ42 levels were significantly elevated in the DS with AD group compared to the DS without AD group. Comparing the DS to the CS group, plasma Aβ40 levels were significantly higher in the DS‐pAD group, while the plasma Aβ42/40 ratio was significantly lower. However, the systematic review presented conflicting findings. For instance, comparing the DS without AD group, some studies reported that plasma Aβ42 levels were higher in the DS with AD group,^82^, ^83^ while others found no difference.^8^, ^39^ Similarly, plasma Aβ40 levels were reported to either increase^39^ or decrease,^82^, ^83^ and some studies indicated an increase in the plasma Aβ42/40 ratio.^82^ When considering AD clinical subgroups (CS, pAD, AD), no significant differences were found in the plasma Aβ42 and Aβ40 levels between these subgroups.^8^, ^39^ Moreover, previous meta‐analyses have also produced varying results. One found that plasma Aβ40 levels were significantly higher, and the Aβ42/40 ratio lower, in the DS with AD group compared to the DS without AD group,^32^ while another reported no significant differences.^99^ Several factors may contribute to these contradictory conclusions. First, discrepancies may arise from differences in plasma measurement techniques. Second, variations in the methods used for AD clinical diagnoses might also play an important role. Third, plasma Aβ40 and the Aβ42/40 ratio may be more closely associated with the early clinical stage of AD pathology in DS people. A previous meta‐analysis identified significant differences in plasma Aβ40 and the Aβ42/40 ratio between DS with and without AD groups.^32^ However, our meta‐analysis did not find the same results between these two groups, but rather observed them between the DS‐CS and DS‐pAD groups. This finding suggests that plasma Aβ40 and the Aβ42/40 ratio could have utility as early biomarkers for detecting AD pathology in DS individuals, potentially allowing for earlier clinical diagnosis and intervention. Finally, the potential overlap of participants in the studies included in our meta‐analysis may have contributed to the marginally significant findings of plasma Aβ42 between the DS with and without AD groups (*z* = 2.03, *p* = 0.04).

### 4.2 Tau

In this meta‐analysis, we examined two forms of tau and found no significant differences in plasma t‐tau (non‐phosphorylated tau) and p‐tau181 (phosphorylated tau) concentrations between the DS population and the euploid controls. The systematic review revealed that some studies supported our meta‐analysis findings,^8^, ^71^ but some studies reported different results.^46^, ^72^, ^73^, ^82^ For instance, a study that used the IMR technique to measure plasma concentrations found that plasma t‐tau levels were significantly elevated in DS individuals compared to euploid controls.^82^ This discrepancy may be due to differences in plasma detection techniques, as our meta‐analysis only included studies using the SIMOA technology. Nevertheless, studies using the SIMOA technique also reported a significant difference in plasma t‐tau and p‐tau181 levels between DS and euploid controls.^46^, ^72^, ^73^ There are two potential reasons for these differing findings. First, the difference in sample size could play a role. The studies in question included ≈ 20 DS individuals and 22 euploid controls,^72^, ^73^ whereas our meta‐analysis reviewed data from 637 DS individuals and 249 euploid controls for plasma t‐tau, as well as 1110 DS people and 503 euploid controls for plasma p‐tau181. Second, the way DS groups were classified may have contributed to the differences. These studies^46^, ^72^, ^73^ did not clinically classify DS individuals based on AD diagnosis, so their DS samples may have included individuals with DS and AD. In contrast, most of the studies we included in our meta‐analysis clinically categorized DS individuals by AD diagnosis, and we categorized those with CS or without AD as the DS group for comparisons to euploid controls. Some studies may provide a possible explanation, as they found no significant differences between euploid controls and DS individuals with CS or pAD but did report significant differences comparing the euploid controls to DS individuals with AD.^39^, ^70^, ^84^ This finding was also supported by a study using amyloid and tau PET imaging,^59^ which observed that plasma t‐tau levels were elevated in amyloid PET–positive DS individuals compared to amyloid PET–negative euploid controls, while no significant difference in plasma t‐tau levels was found in amyloid PET–negative DS individuals.

In addition, in our meta‐analysis, comparing DS with AD to DS without AD, both plasma t‐tau and p‐tau181 levels were significantly elevated in the DS with AD group. Furthermore, plasma p‐tau181 showed significant differences between DS‐pAD and CS, as well as between DS with AD and pAD. In contrast, plasma t‐tau showed significant differences only between DS‐AD and DS‐pAD. Misfolded tau isoforms can spread as seeds between different species, resulting in tau pathology in the recipient's brain.^102^ This process can lead to changes in the conformation of recipient tau, which may be a key mechanism in the transmission of AD pathology.^102^ Our systematic review found that, despite conflicting results from two studies using IMR technology,^82^, ^83^ the studies using SIMOA to measure plasma concentrations consistently supported the findings of our meta‐analysis.^39^, ^70^, ^78^, ^84^ Imaging studies support that plasma t‐tau concentrations in amyloid PET–positive DS individuals, regardless of whether they are tau PET negative or tau PET positive, showed significant differences compared to amyloid and tau PET–negative DS individuals.^59^ Furthermore, plasma p‐tau181 levels are significantly elevated in amyloid PET–positive DS individuals compared to amyloid PET–negative DS individuals.^70^ It is important to note that one study, comparing plasma t‐tau levels between DS with AD and pAD, found a marginally significant difference that did not reach statistical significance.^39^ This may be attributed to insufficient sample size in that particular study. However, when we combined multiple studies of plasma t‐tau in our meta‐analysis, we observed a highly significant effect (*z* = 4.69, *p* < 0.00001). In other words, plasma t‐tau might play a critical role in the later clinical stages of AD pathology as its significant increase could serve as a marker of advanced neurodegeneration so that it might enhance diagnostic accuracy for clinical late‐stage AD, guide more targeted therapeutic interventions, and provide a reliable biomarker for monitoring disease progression and treatment efficacy in clinical trials.

In addition to performing a meta‐analysis on plasma t‐tau and p‐tau181, we conducted a systematic review of other tau biomarkers, as the current number of studies is insufficient for meta‐analysis. Nevertheless, several tau biomarkers show promising potential for future research. For example, plasma NT1‐tau and neuronal exosome p‐s396 tau have demonstrated significant differences between DS individuals and euploid controls,^75^, ^81^ with plasma NT1‐tau levels increasing with age and being associated with poorer cognitive outcomes in DS.^48^ Furthermore, while plasma p‐tau217 has been identified as a highly promising biomarker in AD studies,^18^, ^28^, ^103^, ^104^, ^105^ research on its role in DS remains limited. However, our systematic review found that plasma p‐tau217 can accurately identify abnormal amyloid PET and tau PET results, particularly when considered in conjunction with age.^59^

### 4.3 NfL

Plasma NfL has the most extensive research data available for meta‐analysis compared to plasma amyloid, tau, and GFAP biomarkers. Our meta‐analysis found that plasma NfL levels were significantly elevated not only in DS individuals compared to euploid controls but also showed significant differences between DS with and without AD, DS with pAD and CS, as well as DS with AD and pAD. These findings were supported by the majority of studies included in the systematic review.^16^, ^39^, ^44^, ^46^, ^69^, ^70^, ^74^, ^75^, ^77^, ^78^ However, some studies presented differing views, suggesting that plasma NfL levels were only significantly elevated in DS with AD compared to euploid controls, with no difference observed in the overall DS group.^16^ Also, some studies did not find significant differences between DS with AD and pAD.^39^, ^74^ The discrepancies in findings may be due to the relatively small number of DS individuals clinically diagnosed with pAD or AD in previous studies, or age differences between studies. For example, one study included only 29 individuals diagnosed with DS‐AD and 32 with DS‐pAD, yielding a marginally significant result that did not reach statistical significance (*p* = 0.065).^39^ In contrast, our meta‐analysis included data from nine studies, comprising 549 DS individuals with AD and 379 with pAD, and revealed highly significant results (*z* = 8.96, *p* < 0.00001).

Plasma NfL emerges as a crucial biomarker in DS due to its association with various clinical and pathological factors. Its correlation with age,^40^, ^69^, ^71^, ^74^, ^77^ basal forebrain volume,^41^ longitudinal dementia status,^69^ amyloid pathology,^57^, ^59^ hippocampal atrophy,^47^, ^57^ plasma p‐tau217 and p‐tau181,^56^ CSF NfL,^39^ and cognitive domains such as verbal fluency,^43^ visuospatial ability,^60^ and episodic memory^60^ highlights its relevance in tracking neurodegeneration in DS individuals. While NfL is not specific to a single central nervous system disease,^106^ its elevated levels consistently indicate axonal injury,^107^ making it a valuable marker for monitoring disease progression, even in the early stages of neurodegeneration. Furthermore, its ability to predict dementia onset and track amyloid‐related pathology offers the potential for earlier diagnosis and to track response to therapeutic interventions in DS people.

### 4.4 Non‐AT(N) biomarkers

Recent research indicates a growing interest in non‐AT(N) blood biomarkers, and plasma GFAP has been identified as particularly influential. GFAP, an essential protein filament found in astrocytes, plays a critical role in synaptic function and has been widely researched in many neurological disorders affecting the brain and spinal cord.^108^ While the number of studies on plasma GFAP in DS is currently limited, both our systematic review and meta‐analysis indicated plasma GFAP levels were significantly elevated not only in DS individuals compared to euploid controls but also showed significant differences between DS with and without AD,^76^ DS with pAD and CS,^76^ as well as DS with AD and pAD. In addition, the correlation of plasma GFAP with amyloid pathology,^59^ cortical thinning,^76^ and cognitive decline^43^ highlights its potential role in tracking disease progression and distinguishing between different stages of neurodegeneration. To fully understand the development and dynamics of this biomarker, further longitudinal studies are needed.

Furthermore, there have been reports of significant differences in the levels of plasma biomarkers (IL‐6, IL‐10, TGF‐β1, etc.)^71^, ^85^, ^86^, ^87^, ^88^, ^89^, ^92^ and serum CRP,^97^ beta‐synuclein,^84^ BDNF,^91^ APL1β25,^98^ APL1β27,^98^ APL1β28,^98^ MHPG,^98^ sTREM2,^93^, ^95^ IL‐6^94^, ^95^ of individuals with DS compared to euploid controls. In addition, while evaluating the condition of AD, there were notable variations in the levels of plasma C3,^87^ DYRK1A,^89^, ^90^ Factor I,^87^ ADNP,^90^ and serum beta‐synuclein^84^ between DS individuals with AD and those without AD. However, further research is required because of the wide range of non‐AT(N) biomarkers and the scarcity of relevant studies.

### 4.5 Strengths and limitations of this review

A key strength of this review lies in its comprehensive search strategy and inclusive approach to selecting biomarkers, allowing for a broad assessment of recent literature in this rapidly evolving field and providing a valuable reference for researchers and clinicians. Another is the decision to include only studies using the SIMOA technology for plasma biomarker measurement in the meta‐analysis to reduce variability from differing measurement techniques and improve the reliability of our meta‐analysis findings.

However, limitations include the small number of studies contributing to each metric in the meta‐analysis, despite a reasonable overall number of studies, which underscores the need for more research in different cohorts and populations. Additionally, the heterogeneity observed in some biomarker analyses indicates variability in the results, necessitating more consistent reporting and subgroup analyses. In addition, the International Working Group (IWG)^109^ has highlighted concerns regarding the over‐reliance on biomarker‐only definitions of AD. While our findings support the AT(N) framework's utility in DS, they also echo IWG's caution against solely relying on biomarkers without clinical context. This underscores the need for a combined clinical–biological approach, especially given the biomarker sensitivity and specificity limitations across different populations.

### 4.6 Future directions

It is crucial that future studies report the mean and SD of plasma biomarker concentrations to enable findings from different cohorts to be pooled in future meta‐analyses. Although we identified a further six studies meeting the inclusion criteria, we were unable to include them due to the lack of subgroup‐specific mean and SD data. To improve the comparability of findings across studies, future research should focus on cross‐normalization of biomarker data obtained from different assay platforms (e.g., SIMOA vs. ELISA). This will help ensure that biomarker data, measured with different technologies, can be harmonized and pooled effectively. Besides, it is necessary to undertake more research on Aβ, tau, and GFAP biomarkers as our findings suggested their potential as indicators for AD in DS as they are presently inadequately represented compared to NfL. Future research should give priority to comparing diverse clinical states, such as CS, pAD, and AD. It should also focus on longitudinal designs, as most current studies are cross‐sectional. To address this gap, future research should prioritize longitudinal studies, and practical strategies could include encouraging collaboration through international consortia or combining existing cohorts to obtain sufficient sample sizes for robust longitudinal analyses. These kinds of investigations are essential to comprehend the temporal dynamics of plasma biomarkers throughout the progression of the disease. Furthermore, it is imperative to conduct biomarker studies in different cohorts of individuals with DS to guarantee the reliability and applicability of the data. The presence of variability and outliers in forest plots emphasized the need to attain more reliable and conclusive results. Moreover, the *APOE* genotype was not taken into consideration in this meta‐analysis, which is noteworthy considering that earlier research has demonstrated that early alterations in plasma p‐tau181 are present in DS people who possess the *APOE* ε4 allele.^42^ Subsequent investigations should examine blood biomarkers such as plasma p‐tau217, which has exhibited encouraging outcomes in AD and has recently advanced in DS research.^56^ For example, a cross‐sectional study with 300 DS participants discovered that plasma p‐tau217 could reliably differentiate between people with aberrant tau PET scans and those with normal scans, especially when paired with age.^59^ Finally, future studies should aim to balance biomarker data with clinical assessments to ensure accurate diagnosis and avoid potential over‐diagnosis in preclinical stages.

## 5 CONCLUSIONS

In conclusion, our work demonstrates the promising potential of the blood‐based AT(N) model for diagnosing AD in individuals with DS. Also, we identified the potential of inflammatory biomarkers, such as GFAP, suggesting the possibility of extending the model to an AT(N)I framework (Table 5). This conclusion is consistent with recent perspective research.^110^ While these findings are encouraging, longitudinal studies are needed to further validate these biomarkers and potentially develop new ones to support early clinical AD diagnosis and facilitate clinical trials and research in the DS population.

**TABLE 5:** Significant biomarkers across clinical diagnostic groups categorized by the AT(N) framework: Summary results from meta‐analysis.

Biomarker category	DS vs. Controls	DS‐AD vs. DS‐NAD	DS‐pAD vs. DS‐CS	DS‐AD vs. DS‐pAD
A	A β42, A β40, A β42/40	A β42	A β40, A β42/40	–
T	–	t‐tau, p‐tau181	p‐tau181	t‐tau, p‐tau181
N	NfL	NfL	NfL	NfL
I	GFAP	GFAP	GFAP	GFAP

Abbreviations: AD, Alzheimer's disease; Aβ, amyloid beta; DS, Down syndrome; DS_AD, Down syndrome with Alzheimer's disease; DS_CS, Down syndrome with cognitively stable; DS_NAD, Down syndrome without Alzheimer's disease; DS_pAD, Down syndrome with prodromal Alzheimer's disease; GFAP, glial fibrillary acidic protein; NC, normal controls; NfL, neurofilament light chain; p‐tau181, phosphorylated tau 181; SD, standard deviation; t‐tau, total tau protein.

## CONFLICT OF INTEREST STATEMENT

Dr. Andre Strydom has received funding from AC Immune and has served as a consultant or advisory board member for ProMIS Neurosciences, Aelis Pharma, Alnylam, and Acta Pharmaceuticals. All other authors declare no conflicts of interest. Author disclosures are available in the supporting information.

## CONSENT STATEMENT

All human subjects involved in this study provided informed consent.

## Supporting information



Supporting Information

Supporting Information

Supporting Information

Supporting Information
