# Supplementary material for: Blood‐based biomarkers for Alzheimer's disease in Down syndrome: A systematic review and meta‐analysis
Source: Alzheimers Dement. 2025 Apr 12;21(4):e70135. doi: 10.1002/alz.70135 (PMC11992652; doi:10.1002/alz.70135)
Supplement: Supplementary file 1 — Supporting Information [file ALZ-21-e70135-s004.docx]

**Table S1. Overview of main findings of blood-based AT(N) biomarkers in Systematic Review**

| Biomarkers | | Platform | Blood Fraction | Results | | | | Other Findings |
| --- | --- | --- | --- | --- | --- | --- | --- | --- |
|  |  |  |  | DS vs. Controls | DS-AD vs. DS-NAD | DS-pAD vs. DS-CS | DS-AD vs DS-pAD |  |
| Amyloid (A) | A$\beta_{42}$ | SIMOA | Plasma | ↑^1-4^ | --^1,3^ | --^1,3^ | --^1,3^ | - ↑ (DS-AD vs. Controls)^1^ - ↑ (DS-pAD vs.Controls)^1^ - ↑ (DS-CS vs. Controls)^1^ - ↑ (DS vs. sAD)^2^ - No correlation between plasma and CSF concentrations of Aβ42.^1^ - Within the DS group, there was a moderate positive association between Aβ42 and t-tau concentrations.^2^ - There were no other moderate or strong associations for molecular concentrations or with age across DS group.^2^ - High Aβ42 was observed in infants. Levels of Aβ42 decreased with age in both DS and controls, but this decrease was greater in DS than controls and became prominent in the third decade of life.^4^ |
|  |  | ELISA |  | ↑^5^ |  |  |  | - The plasma Aβ40 and Aβ42 levels were negatively associated with the Gesell Developmental Schedules scores in children with DS.^6^ - Functionalized liposomes effectively reduced free Aβ42 levels in plasma samples from both AD and DS people.^7^ |
|  |  | IMR |  | ↓^8^ | ↑^8,9^ |  |  | - ↑ (DS-NADvs.Controls)^8^ - ↓ (DS-NADvs.Controls)^9^ - Composite biomarker scores including both plasma tau and β-amyloid levels correlate with dementia in DS better than using individual biomarker scores.^9^ |
|  |  | ECL |  | ↑^10^ |  |  |  | - There was no association of age with Aβ42 levels in DS group.^10^ |
|  |  | ELISA |  |  |  |  |  | - Aβ42 decreased with age.^11^ |
|  |  |  | Neuronal exomes | ↑^12^ |  |  |  |  |
|  | A$\beta_{40}$ | SIMOA | Plasma | ↑^1,2^ | ↑^1^ | --^1^ | --^1^ | - ↑ (DS-AD vs. Controls) ^1^ - ↑ (DS-pAD vs.Controls)^1^ - ↑ (DS-CS vs. Controls) ^1^ - ↑ (DS vs. sAD)^2^ - No correlation between plasma and CSF concentrations of Aβ40.^1^ - There were no other moderate or strong associations for molecular concentrations or with age across DS group.^2^ |
|  |  | ELISA |  | ↑^5^ |  |  |  |  |
|  |  | IMR |  | ↑^8^ | ↓^8,9^ |  |  | - ↓ (DS-NADvs.Controls)^8^ - ↑ (DS-NADvs.Controls)^9^ |
|  | A$\beta_{42/40}$ | SIMOA | Plasma | ↓^2^ |  |  |  | - The Aβ42/Aβ40 ratio was higher for controls compared to both individuals with DS and those with sAD.^2^ - Lower plasma Aβ42/40 was related to lower visuospatial ability.^13^ - Decline in the Aβ42/Aβ40 ratio was observed starting with participants at about 45 years of age. Aβ42/40 ratio was negatively correlated with NfL and p-tau181. The amyloid (A) biomarker measure of Aβ1-42/Aβ1-40 appears to change earliest with an initial increase in ratio followed inflection point in the mid-40s and then progressive lowering thereafter.^14^ |
|  |  | IMR |  | ↓^8^ | ↑^8^ |  |  | - ↑ (DS-NADvs.Controls)^8^ |

(Continues)

| Biomarkers | | Platform | Blood Fraction | Results | | | | Other Findings |
| --- | --- | --- | --- | --- | --- | --- | --- | --- |
|  |  |  |  | DS vs. Controls | DS-AD vs. DS-NAD | DS-pAD vs. DS-CS | DS-AD vs DS-pAD |  |
| Tau (T) | t-tau | SIMOA | Plasma | ↑^15,16^ | ↑^1,17^ | --^1^ | --^1^ | - ↑ (DS-AD vs. Controls)^1^ - --(DS-pAD vs. Controls) ^1^ - --(DS-CS vs. Controls)^1^ - T-tau showed a mild plasma–CSF correlation in DS.^1^ - Levels of plasma t-tau in the DS group increased age-dependently.^15^ - There were no moderate or strong associations for molecular concentrations or with age across DS group.^2^ - Plasma t-tau was significantly increased in Aβ-PET-positive tau-PET-positive (A+T+) DS and A+T– DS compared with A–T– DS. Plasma t-tau was increased in A+ DS but not in A– DS, compared with A– non-DS siblings.^18^ - For MCI-DS participants, total tau alone had a lower accuracy. However, a combined model including Nf-L, total-tau, age, and gender improved accuracy to 87%. Age and total-tau were linked to increased risk for MCI-DS.^17^ - Higher total tau was related to lower episodic memory.^13^ - Due to the small sample size, statistical analysis was not conducted, but elevated NfL and total tau levels suggest that DS individuals with acute regression may be at higher risk for early-onset AD.^19^ |
|  |  |  |  | --^2,3^ |  |  | ↑^17^ |  |
|  |  | IMR |  | ↑^8^ | --^8^ |  |  | ↑ (DS-NAD vs. Controls) ^9^ |
|  |  |  |  |  | ↓^9^ |  |  |  |
|  |  | Luminex |  |  |  |  |  | - A significant positive relationship was observed between plasma t-tau levels and connectivity in certain default mode network regions.^20^ |
|  | P-tau 217 | ECL | Plasma |  |  |  |  | - Strong correlations were seen between p-tau217 and p-tau181. The P-tau 217 has a progressively upwards trajectory that becomes apparent in the early to mid-40s, while the neurodegenerative biomarkers of GFAP and NfL are also both informative. The increase in p-tau217 by age 40 is evidence for hyperphosphorylated tau protein 10–15 years before the average age of symptom onset.^14^ - Plasma p-tau217 was significantly increased in Aβ-PET-positive tau-PET-positive (A+T+) DS and A+T– DS compared with A–T– DS. Plasma p-tau217 levels were also significantly higher in A+T+ DS than A+T– DS. A composite measure of p-tau217 and age showed the highest accuracy for distinguishing participants with DS and abnormal tau-PET scans from those with normal tau-PET scans. Plasma p-tau217 was increased in A+ DS but not in A– DS, compared with A– non-DS siblings. Higher levels of p-tau 217 were consistently associated with worse performance on cognitive tests. In DS, plasma p-tau217 accurately identifies individuals with abnormal tau-PET and Aβ-PET scans (especially when combined with age).^18^ - P-tau 217 correlated with NfL.^21^ |
|  | NT1-tau | SIMOA | Plasma | ↑^4^ |  |  |  | - High NT1 tau was observed in infants. NT1 tau fell in adolescents and young adults but increased in older individuals with DS.^4^ - NT1tau increased with age and worse cognition in DS.^11^ |
|  | P-S396-tau | ELISA | Neuronal exomes | ↑^12^ |  |  |  |  |

(Continuous)

| Biomarkers | | Platform | Blood Fraction | Results | | | | Other Findings |
| --- | --- | --- | --- | --- | --- | --- | --- | --- |
|  |  |  |  | DS vs. Controls | DS-AD vs. DS-NAD | DS-pAD vs. DS-CS | DS-AD vs DS-pAD |  |
|  | p-tau 181 | SIMOA | Plasma | ↑^22^ | ↑^23,24^ | ↑^23^ | ↑^23^ | - ↑ (DS-AD vs. Controls)^24^ - --(DS-CS vs. Controls)^23^ - There was a significant correlation between the plasma levels of p-tau181 and age in the DS group.^22^ - APOE ε4 allele carriers showed lower levels of the CSF Aβ1-42 to Aβ1-40 ratio until age 40 years, earlier increases in amyloid PET and plasma pTau181, and earlier loss of cortical metabolism and hippocampal volume, compared with APOE ε4 allele noncarriers.^25^ - There were no significant differences in plasma or CSF p-tau181 concentrations between males and females. However, between the ages of 40 and 50 years, female showed a trend towards higher p-tau181 concentrations in CSF and plasma.^26^ - Basal forebrain volumes significantly correlated with plasma p-tau181.^27^ - The differences in diagnostic accuracy between plasma p-tau181 and plasma NfL were not statistically significant.^23^ - In subjects with DS, plasma p-tau181 concentration significantly correlated with plasma NfL, CSF ratio Aβ1-42/1-40, CSF t-tau, CSF p-tau 181, and CSF NfL concentration.^23^ - Plasma p-tau181 concentration correlated with atrophy measured by MRI in characteristic AD regions including the temporal regions angular and supramarginal gyri and praecuneus of both hemispheres.^23^ - The mean plasma p-tau181 concentration was higher in participants with DS that had a positive amyloid PET scan compared with those with a negative amyloid status.^23^ - Plasma p-tau 181 levels increased with age in DS.^28^ - Elevated plasma p-tau181levels were associated with grey matter volume reduction in the hippocampus and cingulate gyri.^29^ |
|  |  | ECL | Plasma |  |  |  |  | - The P-tau 181 has a progressively upwards trajectory that becomes apparent in the early to mid-40s, while the neurodegenerative biomarkers of GFAP and NfL are also both informative. The increase in p-tau181 by age 40 is evidence for hyperphosphorylated tau protein 10–15 years before the average age of symptom onset.^14^ |
|  |  | ELISA | Neuronal exomes | ↑^12^ |  |  |  |  |

(Continues)

| Biomarkers | | Platform | Blood Fraction | Results | | | | Other Findings |
| --- | --- | --- | --- | --- | --- | --- | --- | --- |
|  |  |  |  | DS vs. Controls | DS-AD vs. DS-NAD | DS-pAD vs. DS-CS | DS-AD vs DS-pAD |  |
| Neurodegeneration (N) | NfL | SIMOA | Plasma | ↑^1,4,16,23,30^ | ↑^1,17,31-34^ | ↑^1,31^ | --^1,31^ | - ↑(DS-AD vs. Controls) ^1,33^ - ↑ (DS-pAD vs. Controls) ^1^ - ↑ (DS-CS vs. Controls) ^1^ - NfL showed a strong plasma–CSF correlation in DS.^1^ - The diagnostic performance of plasma NfL was good for the differentiation of the DS_CS group versus the DS_pAD group and the DS_CS group versus the DS_AD group.^1^ - In the group with DS, only NfL concentration showed a moderate positive association with age.^2^ - No differences were found in plasma NfL when compared APOE ε4 allele carriers with APOE ε4 allele noncarriers.^25^ - Males and females showed overall similar increases in CSF and plasma NfL with age.^26^ - Basal forebrain volumes significantly correlated with plasma NfL.^27^ - Plasma NFL was significantly different at age 30 years (20 years before pAD diagnosis).^3^ - Plasma NfL was increased in Aβ-PET-positive DS and in Aβ-PET-negative DS, compared with Aβ-PET-negative non-DS siblings.^18^ - Significant associations of age, sex, and intellectual disability with baseline plasma NfL concentrations. Male participants showed 14·8% lower concentrations of plasma NfL than female participants.^31^ - Low NfL were observed in infants.^4^ - The negative association between the number of correct words and levels of NfL, with fewer words in the verbal fluency task being correlated with elevated levels of NfL, and driven by age (predominantly by older adults).^35^ - NF-L concentrations increased with age, with a steep increase after age 40, and they were predictive of dementia status, but they showed no relationship with long-standing epilepsy or premorbid ability. Baseline NF-L concentrations were associated with longitudinal dementia status.^32^ - Significant correlations between NF-L plasma concentrations and amyloid pathology and significant inverse correlations with regional glucose metabolism in 5 of 6 regions examined, which were Anterior cingulate, Posterior cingulate, Lateral Temporal, Frontal cortex, Parietal cortex, Praecuneus, and with hippocampal volume; and an inverse correlation with direct measures of cognition and positive correlation with CANTAB Paired Associates Learning (PAL) error rate.^36^ - The age-dependent elevation was steeper in the DS compared with the control group.^30^ - Nf-L alone produced a high accuracy in distinguishing DS-AD participants from cognitively stable (CS) individuals, and when combined with total tau, age, and gender, the accuracy increased to 93%. Higher levels of Nf-L, total tau, and age were associated with greater risk for DS-AD. For MCI-DS participants, Nf-L alone had a lower accuracy. However, a combined model including Nf-L, total-tau, age, and gender improved accuracy to 87%.^17^ - Hippocampus volume, left anterolateral EC (alEC) thickness, and plasma NfL were correlated with each other and were associated with memory. Plasma NfL mediated the relationship between left alEC thickness and memory as well as hippocampus volume and memory.^37^ - Higher NfL was related to lower visuospatial ability and lower episodic memory.^13^ - The strong correlations seen between NfL and p-tau217 or p-tau181, respectively.^14^ - Due to the small sample size, statistical analysis was not conducted, but elevated NfL and total tau levels suggest that DS individuals with acute regression may be at higher risk for early-onset AD.^19^ - Plasma NfL concentration was higher in the overall sample of DS participants with cortical microinfarcts (CMI) than those without.^38^ - Elevated plasma NfL levels were associated with grey matter volume reduction in the hippocampus and cingulate gyri.^29^ - White matter hyperintensity volume correlated with plasma NfL.^21^ |
|  |  |  |  | --^33^ |  |  | ↑^17^ |  |
|  |  | ECL | Plasma |  |  |  |  | - Neurodegeneration protein concentrations were associated with markers of cerebrovascular disease, particularly among individuals with symptoms of AD.^39^ |

(Continues)

| Biomarkers | | | | Platform | Blood Fraction | Results | | | | Other Findings |
| --- | --- | --- | --- | --- | --- | --- | --- | --- | --- | --- |
|  |  |  |  |  |  | DS vs. Controls | DS-AD vs. DS-NAD | DS-pAD vs. DS-CS | DS-AD vs DS-pAD |  |
| Non-AT(N) | Inflammation | Astrocytes | GFAP | SIMOA | Plasma |  | ↑^40^ | ↑^40^ |  | - Plasma GFAP was significantly increased in Aβ-PET-positive tau-PET-positive (A+T+) DS compared with A–T– DS. Plasma GFAP was increased in Aβ-PET-positive DS and in Aβ-PET-negative DS, compared with Aβ-PET-negative non-DS siblings.^18^ - The negative association between the number of correct words and levels of GFAP, with fewer words in the verbal fluency task being correlated with elevated levels of GFAP, and driven by age (predominantly by older adults).^35^ - The early elevations in plasma GFAP levels from mid-late 20s (more than 20 years before symptom onset) in parallel with the reported decreases in CSF Aβ levels. Plasma GFAP presented the highest diagnostic performance to discriminate symptomatic from asymptomatic groups and its concentrations were significantly higher in progressors vs non-progressors, showing an increase of 19.8% per year in participants with dementia. Finally, plasma GFAP levels were highly correlated with cortical thinning and brain amyloid pathology.^40^ - Plasma GFAP correlated with NfL.^21^ |
|  |  |  | | ECL | Plasma |  |  |  |  | - Inflammatory protein concentrations were associated with markers of cerebrovascular disease, particularly among individuals with symptoms of AD.^39^ |
|  |  |  |  |  | Plasma and Serum |  |  |  |  | - Proteomic profiles showed excellent detection accuracy for MCI-DS and DS-AD.^41-43^ |

(Continues)

| Biomarkers | | | | Platform | Blood Fraction | Results | | | | Other Findings |
| --- | --- | --- | --- | --- | --- | --- | --- | --- | --- | --- |
|  |  |  |  |  |  | DS vs. Controls | DS-AD vs. DS-NAD | DS-pAD vs. DS-CS | DS-AD vs DS-pAD |  |
| Non-AT(N) | Inflammation | Microglial | sTREM2 | ELISA | Plasma |  |  |  |  | - Plasma TREM2 levels decreased with age in DS.^28,44^ |
|  |  |  |  | Western blotting | Serum | ↓^44,45^ |  |  |  |  |
|  |  |  |  | Luminex System | Plasma | ↑^46^ |  |  |  | - In young adults with DS, sTREM2 correlated positively with 24 of the measured cytokines, whereas there were no significant correlations in the control group.^46^ - Plasma sTREM2 levels had a positive correlation with connectivity in some anterior default mode network regions.^20^ |
|  |  | Mediator | HMFBI | ELISA | Plasma | ↑^47-49^ |  |  |  |  |
|  |  |  | MMP-3 | Western blotting |  | ↑^50^ |  |  |  |  |
|  |  |  | tPA |  |  | ↓^50^ |  |  |  |  |
|  |  | Cytokines | IL6,  IL10 | SIMOA | Plasma | --^2^ |  |  |  | - IL1β ↑ (DS vs. sAD)^2^ - Both the groups with DS and with sAD showed a moderate positive association between IL10 and TNFα concentrations.^2^ - The control group showed a moderate negative association between the Aβ42/Aβ40 ratio and IL10 concentration.^2^ - In DS group, there was a strong positive association between IL1β and IL10, and a moderate positive association between IL6 and TNFα.^2^ - In DS group, IL1β concentration showed a moderate positive association with t-tau concentration and a moderate negative association with the Aβ42/t-tau ratio.^2^ - A negative correlation between low TGF-β1 concentrations and high TNF-α plasma concentrations.^51^ |
|  |  |  |  | ELISA |  | ↑^47^ |  |  |  |  |
|  |  |  |  | ELISA | Serum | --^52^ |  |  |  |  |
|  |  |  | IL6 | ELISA | Serum | ↑^45,53^ |  |  |  |  |
|  |  |  | TNFα | SIMOA | Plasma | --^2^ |  |  |  |  |
|  |  |  |  | ELISA | Serum | --^52^ |  |  |  |  |
|  |  |  | TGF-β1 | ELISA | Plasma | ↓^51^ |  |  |  |  |
|  |  |  |  |  | Serum | --^52^ |  |  |  |  |
|  |  |  | IL-2 | ELISA | Plasma | ↑^47^ |  |  |  |  |
|  |  |  |  |  | Serum | --^52^ |  |  |  |  |
|  |  |  | IL-1α,  IL-12,  MCP-1 | ELISA | Serum | --^52^ |  |  |  |  |
|  |  |  | IL-1ra,  GM-CSF,  Epo | ELISA | Plasma | ↑^47-49^ |  |  |  |  |
|  |  |  | IL1β | SIMOA | Plasma | ↑^2^ |  |  |  |  |

(Continues)

| Biomarkers | | | | Platform | Blood Fraction | Results | | | | Other Findings |
| --- | --- | --- | --- | --- | --- | --- | --- | --- | --- | --- |
|  |  |  |  |  |  | DS vs. Controls | DS-AD vs. DS-NAD | DS-pAD vs. DS-CS | DS-AD vs DS-pAD |  |
| Non-AT(N) | Complement | Activation products | TCC, iC3b | ELISA | Plasma | ↑^47-49^ |  |  |  | - Neither APOE genotype nor CLU SNPs impacted complement levels, while rs6656401 in CR1 significantly impacted plasma sCR1 levels.^48^ |
|  |  | Proteins | C1q,  C9 |  |  | ↑^47-49^ |  |  |  |  |
|  |  |  | C3 |  |  | ↑^48^ | ↓^48^ |  |  |  |
|  |  | Regulators | C1 inhibitor, factor H, FHR4, clusterin |  |  | ↑^47-49^ |  |  |  |  |
|  |  |  | Factor I |  |  | ↓^48^ | ↓^48^ |  |  |  |
|  |  |  | sCR1 |  |  | ↓^48^ |  |  |  |  |
|  | DYRK1A | full-length form | | Solid phase immobilized epitope immunoassay | Plasma |  | ↓^54^ | ↓^54^ | --^54^ | - ↑ (DS-CS vs. Controls)^54^ |
|  |  | truncated form | |  |  |  |  |  |  | - ↑ (DS-CS vs. Controls)^54^ |
|  |  |  | | ECL |  | ↑^55^ | ↓^55^ |  |  |  |
|  | ADNP |  | | Sandwich ELISA | Plasma |  | ↓^54^ | ↓^54^ | ↑^54^ | - ↑ (DS-CS vs. Controls)^54^ |
|  | NGF | proNGF | | Western blotting | Plasma | ↑^50^ |  |  |  |  |
|  | Metabolomics |  | |  | Plasma |  |  |  |  | - DS produce elevated levels of kynurenine and quinolinic acid, two tryptophan catabolites with potent immunosuppressive and neurotoxic properties, respectively.^56^ |
|  | Synuclein | Beta-Synuclein | | IP-MS | Serum | ↑^24^ | ↑^24^ |  |  |  |
|  | Neurotrophic factor | BDNF | | ELISA | Serum | ↑^52^ |  |  |  |  |
|  | Acute phase protein | C-RP | | LEN | Serum | ↑^49^ |  |  |  |  |
|  |  | SAA | | ELISA | Plasma | ↑^47-49^ |  |  |  |  |
|  | GF | VEGF | | ELISA | Plasma | ↑^47-49^ |  |  |  |  |
|  | APP processing | APL1β25, APL1β27, APL1β28 | | ELISA | Serum | ↓^57^ |  |  |  | - A significant positive correlation between IQCODE short score and APL1β25 serum level in DS people. |
|  | Neurotransmitter metabolism | MHPG | | ELISA | Serum | ↓^57^ |  |  |  |  |
|  |  |  |  | RP-HPLC | Plasma |  | ↓^58^ |  |  |  |

Abbreviations: AD = Alzheimer's disease; DS = Down syndrome; NC = normal controls; DS_NAD = Down syndrome without Alzheimer’s disease; DS_CS = Down syndrome with cognitively stable; DS_pAD = Down syndrome with prodromal Alzheimer’s disease; DS_AD = Down syndrome with Alzheimer’s disease; SD = standard deviation; Aβ = Amyloid β; total tau = Total Tau protein; p-tau 181 = Phosphorylated tau 181; p-tau217 = Phosphorylated tau 217, NT1-tau = N-terminal tau fragment; p-S396-tau = Phosphorylated tau S396; NfL = Neurofilament light; GFAP = Glial fibrillary acidic protein; SIMOA = Single Molecule Array; IMR = Immunomagnetic Reduction; ECL = Electrochemiluminescence; ELISA = Enzyme-Linked Immunosorbent Assay; IP-MS = Immunoprecipitation-Mass Spectrometry; LEN = Latex-Enhanced Nephelometry; TREM2 = Triggering receptor expressed on myeloid cells 2; HMGB1 = High mobility group box 1; MMP-3 = matrix metalloproteinase-3; tPA = Tissue plasminogen activator; IL-6 = Interleukin-6; sTREM2 = soluble TREM; IL-10 = interleukin-10; TNFα = tumour necrosis factor-alpha; TGF-β1 = transforming growth factor-beta 1; IL-2 = interleukin-2; IL-1α = interleukin-1 alpha; IL-12 = interleukin-12; MCP-1 = monocyte chemoattractant protein-1; IL-1ra = interleukin-1 receptor antagonist; GM-CS = granulocyte-macrophage colony-stimulating; IL-1β = interleukin-1 beta; Epo = erythropoietin; TCC = terminal complement; iC3b = inactivated complement component 3b; C1q = complement component 1q; C9 = complement component 9; C3 = complement component 3 (C3); FHR4 = factor H-related protein 4; sCR1 = soluble complement receptor 1; DYRK1A = dual-specificity tyrosine-phosphorylation-regulated kinase 1A; ADNP = activity-dependent neuroprotective protein; NGF = Neurotrophic factors like nerve growth factor; BDNF = brain-derived neurotrophic factor; C-RP = C-reactive protein; SAA = serum amyloid A; VEGF = vascular endothelial growth factor; APL1β25 = Amyloid Precursor-Like Protein 1 Beta 25; APL1β27 = Amyloid Precursor-Like Protein 1 Beta 27; APL1β28 = Amyloid Precursor-Like Protein 1 Beta 28; MHPG = 3-Methoxy-4-Hydroxyphenylglycol; APP = Amyloid Precursor Protein; RP-HPLC = reversed phase high-performance liquid chromatography.

**References**

1. Fortea J, Carmona-Iragui M, Benejam B, et al. Plasma and CSF biomarkers for the diagnosis of Alzheimer's disease in adults with Down syndrome: a cross-sectional study. *Lancet Neurol*. Oct 2018;17(10):860-869. doi:10.1016/s1474-4422(18)30285-0

2. Startin CM, Ashton NJ, Hamburg S, et al. Plasma biomarkers for amyloid, tau, and cytokines in Down syndrome and sporadic Alzheimer's disease. *Alzheimers Res Ther*. Mar 21 2019;11(1):26. doi:10.1186/s13195-019-0477-0

3. Fortea J, Vilaplana E, Carmona-Iragui M, et al. Clinical and biomarker changes of Alzheimer's disease in adults with Down syndrome: a cross-sectional study. *The Lancet*. 2020;395(10242):1988-1997. doi:10.1016/S0140-6736(20)30689-9

4. Mengel D, Liu W, Glynn RJ, et al. Dynamics of plasma biomarkers in Down syndrome: the relative levels of Aβ42 decrease with age, whereas NT1 tau and NfL increase. *Alzheimers Res Ther*. Mar 19 2020;12(1):27. doi:10.1186/s13195-020-00593-7

5. Morsiani C, Bacalini MG, Collura S, et al. Blood circulating miR-28-5p and let-7d-5p associate with premature ageing in Down syndrome. *Mechanisms of Ageing and Development*. 2022/09/01/ 2022;206:111691. doi:<https://doi.org/10.1016/j.mad.2022.111691>

6. Yang J, Hu L, Zhang Y, Shi Y, Jiang W, Song C. Gesell Developmental Schedules scores and the relevant factors in children with Down syndrome. *J Pediatr Endocrinol Metab*. Apr 28 2020;33(4):539-546. doi:10.1515/jpem-2019-0236

7. Conti E, Gregori M, Radice I, et al. Multifunctional liposomes interact with Abeta in human biological fluids: Therapeutic implications for Alzheimer's disease. *Neurochemistry International*. 2017/09/01/ 2017;108:60-65. doi:<https://doi.org/10.1016/j.neuint.2017.02.012>

8. Lee N-C, Yang S-Y, Chieh J-J, et al. Blood Beta-Amyloid and Tau in Down Syndrome: A Comparison with Alzheimer’s Disease. *Frontiers in Aging Neuroscience*. 2017;8doi:10.3389/fnagi.2016.00316

9. Fang WQ, Hwu WL, Chien YH, et al. Composite Scores of Plasma Tau and β-Amyloids Correlate with Dementia in Down Syndrome. *ACS Chem Neurosci*. Jan 15 2020;11(2):191-196. doi:10.1021/acschemneuro.9b00585

10. Mehta PD, Patrick BA, Miller DL, Coyle PK, Wisniewski T. A Sensitive and Cost-Effective Chemiluminescence ELISA for Measurement of Amyloid-β 1-42 Peptide in Human Plasma. *Journal of Alzheimer's Disease*. 2020;78(3)doi:10.3233/JAD-200861

11. Stern AM, Van Pelt KL, Liu L, et al. Plasma NT1-tau and Aβ(42) correlate with age and cognitive function in two large Down syndrome cohorts. *Alzheimers Dement*. Dec 2023;19(12):5755-5764. doi:10.1002/alz.13382

12. Hamlett ED, Goetzl EJ, Ledreux A, et al. Neuronal exosomes reveal Alzheimer's disease biomarkers in Down syndrome. *Alzheimers Dement*. May 2017;13(5):541-549. doi:10.1016/j.jalz.2016.08.012

13. Schworer EK, Handen BL, Petersen M, et al. Cognitive and functional performance and plasma biomarkers of early Alzheimer's disease in Down syndrome. *Alzheimer's & Dementia: Diagnosis, Assessment & Disease Monitoring*. 2024;16(2)doi:10.1002/dad2.12582

14. Hendrix JA, Airey DC, Britton A, et al. Cross-Sectional Exploration of Plasma Biomarkers of Alzheimer's Disease in Down Syndrome: Early Data from the Longitudinal Investigation for Enhancing Down Syndrome Research (LIFE-DSR) Study. *J Clin Med*. Apr 28 2021;10(9)doi:10.3390/jcm10091907

15. Kasai T, Tatebe H, Kondo M, et al. Increased levels of plasma total tau in adult Down syndrome. *PLOS ONE*. 2017;12(11)doi:10.1371/journal.pone.0188802

16. Araya P, Kinning KT, Coughlan C, et al. IGF1 deficiency integrates stunted growth and neurodegeneration in Down syndrome. *Cell Rep*. Dec 27 2022;41(13):111883. doi:10.1016/j.celrep.2022.111883

17. Petersen ME, Rafii MS, Zhang F, et al. Plasma Total-Tau and Neurofilament Light Chain as Diagnostic Biomarkers of Alzheimer’s Disease Dementia and Mild Cognitive Impairment in Adults with Down Syndrome. *Journal of Alzheimer's Disease*. 2021;79(2)doi:10.3233/JAD-201167

18. Janelidze S, Christian BT, Price J, et al. Detection of Brain Tau Pathology in Down Syndrome Using Plasma Biomarkers. *JAMA Neurol*. Aug 1 2022;79(8):797-807. doi:10.1001/jamaneurol.2022.1740

19. Handen B, Clare I, Laymon C, et al. Acute Regression in Down Syndrome. *Brain Sci*. Aug 23 2021;11(8)doi:10.3390/brainsci11081109

20. Koenig KA, Bekris LM, Ruedrich S, et al. High-resolution functional connectivity of the default mode network in young adults with down syndrome. *Brain Imaging Behav*. Aug 2021;15(4):2051-2060. doi:10.1007/s11682-020-00399-z

21. Edwards NC, Lao PJ, Alshikho MJ, et al. Cerebrovascular disease is associated with Alzheimer’s plasma biomarker concentrations in adults with Down syndrome. *Brain Communications*. 2024;6(5):fcae331. doi:10.1093/braincomms/fcae331

22. Tatebe H, Kasai T, Ohmichi T, et al. Quantification of plasma phosphorylated tau to use as a biomarker for brain Alzheimer pathology: pilot case-control studies including patients with Alzheimer’s disease and down syndrome. *Molecular Neurodegeneration 2017 12:1*. 2017;12(1)doi:10.1186/s13024-017-0206-8

23. Lleó A, Zetterberg H, Pegueroles J, et al. Phosphorylated tau181 in plasma as a potential biomarker for Alzheimer’s disease in adults with Down syndrome. *Nature Communications*. 2021/07/14 2021;12(1):4304. doi:10.1038/s41467-021-24319-x

24. Oeckl P, Wagemann O, Halbgebauer S, et al. Serum Beta-Synuclein Is Higher in Down Syndrome and Precedes Rise of pTau181. *Ann Neurol*. Jul 2022;92(1):6-10. doi:10.1002/ana.26360

25. Bejanin A, Iulita MF, Vilaplana E, et al. Association of Apolipoprotein E ɛ4 Allele With Clinical and Multimodal Biomarker Changes of Alzheimer Disease in Adults With Down Syndrome. *JAMA Neurology*. 2021;78(8)doi:10.1001/jamaneurol.2021.1893

26. Iulita MF, Bejanin A, Vilaplana E, et al. Association of biological sex with clinical outcomes and biomarkers of Alzheimer's disease in adults with Down syndrome. *Brain Commun*. 2023;5(2):fcad074. doi:10.1093/braincomms/fcad074

27. Aranha MR, Iulita MF, Montal V, et al. Basal forebrain atrophy along the Alzheimer's disease continuum in adults with Down syndrome. *Alzheimer's & Dementia*. 2023;19(11)doi:10.1002/alz.12999

28. Raha-Chowdhury R, Henderson JW, Raha AA, et al. Choroid Plexus Acts as Gatekeeper for TREM2, Abnormal Accumulation of ApoE, and Fibrillary Tau in Alzheimer’s Disease and in Down Syndrome Dementia. *Journal of Alzheimer's Disease*. 2019;69(1)doi:10.3233/JAD-181179

29. Sánchez-Moreno B, Zhang L, Mateo G, et al. Voxel-based dysconnectomic brain morphometry with computed tomography in Down syndrome. *Ann Clin Transl Neurol*. Jan 2024;11(1):143-155. doi:10.1002/acn3.51940

30. Shinomoto M, Kasai T, Tatebe H, et al. Plasma neurofilament light chain: A potential prognostic biomarker of dementia in adult Down syndrome patients. *PLoS One*. 2019;14(4):e0211575. doi:10.1371/journal.pone.0211575

31. Carmona-Iragui M, Alcolea D, Barroeta I, et al. Diagnostic and prognostic performance and longitudinal changes in plasma neurofilament light chain concentrations in adults with Down syndrome: a cohort study. *Lancet Neurol*. Aug 2021;20(8):605-614. doi:10.1016/s1474-4422(21)00129-0

32. Strydom A, Heslegrave A, Startin CM, et al. Neurofilament light as a blood biomarker for neurodegeneration in Down syndrome. *Alzheimer's Research & Therapy*. 2018/04/10 2018;10(1):39. doi:10.1186/s13195-018-0367-x

33. Ashton NJ, Janelidze S, Al Khleifat A, et al. A multicentre validation study of the diagnostic value of plasma neurofilament light. *Nature Communications*. 2021/06/07 2021;12(1):3400. doi:10.1038/s41467-021-23620-z

34. Pape SE, al Janabi T, Ashton NJ, et al. The reliability and validity of DSM 5 diagnostic criteria for neurocognitive disorder and relationship with plasma neurofilament light in a down syndrome population. *Scientific Reports*. 2021/06/29 2021;11(1):13438. doi:10.1038/s41598-021-92887-5

35. Mgaieth F, Baksh RA, Startin CM, et al. Exploring semantic verbal fluency patterns and their relationship to age and Alzheimer's disease in adults with Down syndrome. *Alzheimers Dement*. Nov 2023;19(11):5129-5137. doi:10.1002/alz.13097

36. Rafii MS, Donohue MC, Matthews DC, et al. Plasma Neurofilament Light and Alzheimer’s Disease Biomarkers in Down Syndrome: Results from the Down Syndrome Biomarker Initiative (DSBI). *Journal of Alzheimer's Disease*. 2019;70(1)doi:10.3233/JAD-190322

37. DiProspero N, Sathishkumar M, Janecek J, et al. Neurofilament light chain concentration mediates the association between regional medial temporal lobe structure and memory in adults with Down syndrome. *Alzheimer's & dementia (Amsterdam, Netherlands)*. 2024;16(1)doi:10.1002/dad2.12542

38. Aranha MR, Montal V, van den Brink H, et al. Cortical microinfarcts in adults with Down syndrome assessed with 3T-MRI. *Alzheimers Dement*. Jun 2024;20(6):3906-3917. doi:10.1002/alz.13797

39. Moni F, Petersen ME, Zhang F, et al. Probing the proteome to explore potential correlates of increased Alzheimer's‐related cerebrovascular disease in adults with Down syndrome. *Alzheimer's & Dementia*. 2022;18(10)doi:10.1002/alz.12627

40. Montoliu-Gaya L, Alcolea D, Ashton NJ, et al. Plasma and cerebrospinal fluid glial fibrillary acidic protein levels in adults with Down syndrome: a longitudinal cohort study. *EBioMedicine*. Apr 2023;90:104547. doi:10.1016/j.ebiom.2023.104547

41. Petersen ME, Zhang F, Schupf N, et al. Proteomic profiles for Alzheimer's disease and mild cognitive impairment among adults with Down syndrome spanning serum and plasma: An Alzheimer's Biomarker Consortium–Down Syndrome (ABC–DS) study. *Alzheimer's & Dementia: Diagnosis, Assessment & Disease Monitoring*. 2020;12(1)doi:10.1002/dad2.12039

42. Petersen M, Zhang F, Krinsky-McHale SJ, et al. Proteomic profiles of prevalent mild cognitive impairment and Alzheimer's disease among adults with Down syndrome. *Alzheimer's & Dementia: Diagnosis, Assessment & Disease Monitoring*. 2020;12(1)doi:10.1002/dad2.12023

43. O'Bryant SE, Zhang F, Silverman W, et al. Proteomic profiles of incident mild cognitive impairment and Alzheimer's disease among adults with Down syndrome. *Alzheimer's & Dementia: Diagnosis, Assessment & Disease Monitoring*. 2020;12(1):e12033. doi:<https://doi.org/10.1002/dad2.12033>

44. Raha-Chowdhury R, Henderson JW, Raha AA, et al. Erythromyeloid-Derived TREM2: A Major Determinant of Alzheimer's Disease Pathology in Down Syndrome. *J Alzheimers Dis*. 2018;61(3):1143-1162. doi:10.3233/jad-170814

45. Raha-Chowdhury R, Raha AA, Henderson J, et al. Impaired Iron Homeostasis and Haematopoiesis Impacts Inflammation in the Ageing Process in Down Syndrome Dementia. *J Clin Med*. Jun 29 2021;10(13)doi:10.3390/jcm10132909

46. Weber GE, Koenig KA, Khrestian M, et al. An Altered Relationship between Soluble TREM2 and Inflammatory Markers in Young Adults with Down Syndrome: A Preliminary Report. *J Immunol*. Mar 1 2020;204(5):1111-1118. doi:10.4049/jimmunol.1901166

47. Huggard D, Kelly L, Ryan E, et al. Increased systemic inflammation in children with Down syndrome. *Cytokine*. 2020/03/01/ 2020;127:154938. doi:<https://doi.org/10.1016/j.cyto.2019.154938>

48. Veteleanu A, Pape S, Davies K, et al. Complement dysregulation and Alzheimer's disease in Down syndrome. *Alzheimers Dement*. Apr 2023;19(4):1383-1392. doi:10.1002/alz.12799

49. Manti S, Cutrupi MC, Cuppari C, et al. Inflammatory biomarkers and intellectual disability in patients with Down syndrome. *Journal of Intellectual Disability Research*. 2018;62(5):382-390. doi:<https://doi.org/10.1111/jir.12470>

50. Pentz R, Iulita MF, Ducatenzeiler A, et al. Nerve growth factor (NGF) pathway biomarkers in Down syndrome prior to and after the onset of clinical Alzheimer's disease: A paired CSF and plasma study. *Alzheimers Dement*. Apr 2021;17(4):605-617. doi:10.1002/alz.12229

51. Grasso M, Fidilio A, L’Episcopo F, et al. Low TGF-β1 plasma levels are associated with cognitive decline in Down syndrome. *Frontiers in Pharmacology*. 2024;15doi:10.3389/fphar.2024.1379965

52. Tarani L, Carito V, Ferraguti G, et al. Neuroinflammatory Markers in the Serum of Prepubertal Children with Down Syndrome. *Journal of Immunology Research*. 2020;2020(1):6937154. doi:<https://doi.org/10.1155/2020/6937154>

53. Raha AA, Ghaffari SD, Henderson J, et al. Hepcidin Increases Cytokines in Alzheimer's Disease and Down's Syndrome Dementia: Implication of Impaired Iron Homeostasis in Neuroinflammation. *Front Aging Neurosci*. 2021;13:653591. doi:10.3389/fnagi.2021.653591

54. Moreau M, Carmona-Iragui M, Altuna M, et al. DYRK1A and Activity-Dependent Neuroprotective Protein Comparative Diagnosis Interest in Cerebrospinal Fluid and Plasma in the Context of Alzheimer-Related Cognitive Impairment in Down Syndrome Patients. *Biomedicines 2022, Vol 10, Page 1380*. 2022;10(6)doi:10.3390/biomedicines10061380

55. Delabar JM, Lagarde J, Fructuoso M, et al. Increased plasma DYRK1A with aging may protect against neurodegenerative diseases. *Transl Psychiatry*. Apr 4 2023;13(1):111. doi:10.1038/s41398-023-02419-0

56. Powers RK, Culp-Hill R, Ludwig MP, et al. Trisomy 21 activates the kynurenine pathway via increased dosage of interferon receptors. *Nature Communications*. 2019/10/18 2019;10(1):4766. doi:10.1038/s41467-019-12739-9

57. Meguid NA, Hemimi M, Elpatrik G, Fouad-Elhady EA, Dardir AA, Ahmed HH. Analysis of Specific Serum Markers for Early Prediction of Alzheimer's Disease in Adolescents with Down Syndrome. *Indian Journal of Clinical Biochemistry*. 2024/04/02 2024;doi:10.1007/s12291-024-01206-y

58. Dekker AD, Vermeiren Y, Carmona-Iragui M, et al. Monoaminergic impairment in Down syndrome with Alzheimer's disease compared to early-onset Alzheimer's disease. *Alzheimers Dement (Amst)*. 2018;10:99-111. doi:10.1016/j.dadm.2017.11.001
