# Supplementary material for: Blood‐based biomarkers for Alzheimer's disease in Down syndrome: A systematic review and meta‐analysis
Source: Alzheimers Dement. 2025 Apr 12;21(4):e70135. doi: 10.1002/alz.70135 (PMC11992652; doi:10.1002/alz.70135)
Supplement: Supplementary file 2 — Supporting Information [file ALZ-21-e70135-s001.docx]

**Table S2. Overview of demographic data, plasma biomarkers levels (pg/ml) and methodology of studies included in Meta-analysis**

(Continues)

(Continues)

(Continues)

(Continues)

(Continues)

Abbreviations: AD = Alzheimer's disease; DS = Down syndrome; NC = normal controls; DS_NAD = Down syndrome without Alzheimer’s disease; DS_CS = Down syndrome with cognitively stable; DS_pAD = Down syndrome with prodromal Alzheimer’s disease; DS_AD = Down syndrome with Alzheimer’s disease; SD = standard deviation; Aβ = Amyloid β; total tau = Total Tau protein; p-tau 181 = Phosphorylated tau 181; NfL = Neurofilament light; GFAP = Glial fibrillary acidic protein.

*In the column of Aβ42/40 ratio indicates calculating using the Delta statistical method.

"NA" refers to cases where data was not available or not applicable.
