# Supplementary material for: Blood‐based biomarkers for Alzheimer's disease in Down syndrome: A systematic review and meta‐analysis
Source: Alzheimers Dement. 2025 Apr 12;21(4):e70135. doi: 10.1002/alz.70135 (PMC11992652; doi:10.1002/alz.70135)
Supplement: Supplementary file 3 — Supporting Information [file ALZ-21-e70135-s003.docx]

# Supplementary materials

Table of Contents

[Supplementary materials 1](#_Toc189501870)

[List of Search Terms for Identifying Relevant Studies 2](#_Toc189501871)

[Inclusion and Exclusion Criteria 3](#_Toc189501881)

[Summary of Included Studies in the Systematic Review 4](#_Toc189501882)

[Quality Assessment of Studies Included in the Meta-Analysis 12](#_Toc189501883)

[Forest and Funnel Plot in the Meta-Analysis 13](#_Toc189501884)

[Sensitivity Analysis 41](#_Toc189501885)

[References 46](#_Toc189501886)

## List of Search Terms for Identifying Relevant Studies

Table S3 List of Search Terms for Identifying Relevant Studies

| Search Term | Description |
| --- | --- |
| Down syndrome | Terms related to Down syndrome (e.g., “Down’s syndrome”, “trisomy 21”) |
| Alzheimer’s OR Alzheimer OR dementia | Terms related to Alzheimer's disease or dementia |
| plasma OR serum OR blood | Terms related to biological sample types (e.g., plasma, serum, blood) |
| amyloid OR tau OR neurofilament light OR glial fibrillary acidic protein OR inflammation OR biomarker OR complement OR cytokines | Terms related to biomarkers, including amyloid, tau, neurofilament light, and cytokines |

## Inclusion and Exclusion Criteria

Table S4 Inclusion and Exclusion Criteria for Systematic Review and Meta-analysis

| Criteria | Systematic review | Meta-analysis |
| --- | --- | --- |
| Population | - Individuals of all age groups with a diagnosis of DS | - Adults (≥18 years old) with Down syndrome  - Comparison groups include:  1) DS with AD vs. DS without AD  2) or DS individuals among clinical subgroups (cognitively stable, prodromal AD, AD)  3) or DS vs. non-DS controls  - AD diagnosis confirmed by expert clinicians using DSM-IV, DSM-V, or NIA-AA (2018) criteria |
| Exposure | - Blood biomarkers associated with AD measured in plasma or serum | - Blood biomarkers measured in plasma by Single Molecule Array (SIMOA) technology |
| Control | - Studies may or may not include a control group (e.g., individuals without DS) | - Studies that provide comparisons of plasma biomarker levels among DS subgroups (e.g., cognitively stable, prodromal AD, AD), between DS individuals with and without AD, or between DS individuals and non-DS controls |
| Study Design | - Original research  - Observational studies (cross-sectional and longitudinal) | - Observational studies only (cross-sectional and longitudinal) |
| Outcomes | - Any study reporting on blood biomarker levels in DS individuals, regardless of control group presence or technology used | - Standardized mean differences in plasma biomarker levels, focusing on:  1) Amyloid beta (Aβ42, Aβ40, Aβ42/40 ratio)  2) Tau protein (total tau, p-tau 181)  3) Neurofilament light chain (NfL)  4) Glial fibrillary acidic protein (GFAP)  - Relationship of biomarkers with dementia status in DS and differences among DS clinical subgroups as well as DS vs. non-DS controls |
| Exclusion | - Studies on individuals with other intellectual disabilities  - Studies not published in English  - Systematic reviews, meta-analyses, reports, and case studies  - Studies published before 2017 | - Studies using non-SIMOA assays for plasma biomarker measurement  - Studies that do not compare DS subgroups, do not include control comparisons, or do not provide necessary data (e.g., mean and SD of each biomarker for each group)  - Studies published before 2017 |

## Summary of Included Studies in the Systematic Review

Table S5 Summary of Included Studies in the Systematic Review

| Study | Sample numbers | Blood fraction | Platform | Biomarkers | Study design | Cohort original | Meta-analysis |
| --- | --- | --- | --- | --- | --- | --- | --- |
| Ashton et al. (2021)^1^ | DS = 29  DS-AD= 12  NC = 130 | Plasma | SIMOA | NfL | Cross-sectional | DS: LonDownS | Included |
| Mehta et al. (2020)^2^ | DS = 25  NC = 50 | Plasma | CL | A$\beta_{42}$ | Cross-sectional | Not specified | Excluded (non-SIMOA platform) |
| Handen et al. (2021)^3^ | DS = 20 | Plasma | SIMOA | A$\beta_{42}$, A$\beta_{40}$, A$\beta_{42/40}$, t-tau, NfL | Cross-sectional | ABC-DS | Excluded (lacks appropriate comparison) |
| Weber et al. (2020)^4^ | DS = 15  NC = 16 | Plasma | Luminex System | sTREM2, cytokines | Cross-sectional | Not specified | Excluded (non-SIMOA platform) |
| Meguid et al. (2024)^5^ | DS = 30  NC = 30 | Serum | ELISA | APL1β25, APL1β27, APL1β28, MHPG | Cross-sectional | Not specified | Excluded (non-SIMOA platform, not plasma) |
| Bejanin et al. (2021)^6^ | DS = 464 | Plasma | SIMOA | p-tau 181, NfL | Cross-sectional | DABNI, Cambridge | Excluded (participants overlapping with Iulita et al. (2023)^7^) |
| Iulita et al. (2023)^7^ | DS = 628 | Plasma | SIMOA | p-tau 181, NfL | Cross-sectional | DABNI, Cambridge | Included |
| Aranha et al. (2023)^8^ | DS = 234  NC = 147 | Plasma | SIMOA | p-tau 181, NfL | Cross-sectional | DABNI, SPIN | Included |
| Lee et al. (2017)^9^ | DS = 35  NC = 78 | Plasma | IMR | A$\beta_{42}$, A$\beta_{40}$, A$\beta_{42/40}$, t-tau | Cross-sectional | National Taiwan University | Excluded (non-SIMOA platform) |
| Morsiani et al. (2022)^10^ | DS = 43  NC = 30 | Plasma | ELISA | A$\beta_{42}$, A$\beta_{40}$, A$\beta_{42/40}$ | Cross-sectional | S. Orsola-Malpighi Hospital | Excluded (non-SIMOA platform) |

(Continues)

| Study | Sample numbers | Blood fraction | Platform | Biomarkers | Study design | Cohort original | Meta-analysis |
| --- | --- | --- | --- | --- | --- | --- | --- |
| Raha-Chowdhury et al. (2019)^11^ | DS = 47  NC = 50 | Plasma | ELISA, SIMOA | A$\beta_{42}$, A$\beta_{40}$, t-tau, p-tau 181, sTREM2 | Longitudinal | Cambridge Developmental Disabilities Research Group | Excluded (non-SIMOA platform, not reported required data) |
| Fortea et al. (2020)^12^ | DS = 388 NC = 242 | Plasma | SIMOA | A$\beta_{42}$, t-tau, NfL | Cross-sectional | DABNI, Cambridge | Included |
| Schworer et al. (2024)^13^ | DS = 260 | Plasma | SIMOA | A$\beta_{42/40}$, t-tau, NfL | Cross-sectional | ABC-DS | Included |
| Veteleanu et al. (2023)^14^ | DS = 71 NC = 46 | Plasma | ELISA | C1q, C3, C4, C9, C1 inhibitor, FI, sCR1, factor H, FHR4, FHR125, clusterin, iC3b, C5a, TCC | Cross-sectional | LonDownS | Excluded (non-SIMOA platform) |
| Fang et al. (2019)^15^ | DS = 73  NC = 77 | Plasma | IMR | A$\beta_{42}$, A$\beta_{40}$, t-tau | Cross-sectional | National Taiwan University | Excluded (non-SIMOA platform) |
| Aranha et al. (2024)^16^ | DS = 195  NC = 106 | Plasma | SIMOA | p-tau 181, NfL | Cross-sectional | DABNI, SPIN | Excluded (not reported required data) |
| Hendrix et al. (2021)^17^ | DS = 90 | Plasma | SIMOA, ECL | A$\beta_{42}$, A$\beta_{40}$, p-tau 181, p-tau 217, NfL, GFAP | Cross-sectional | LIFE-DSR | Excluded (no comparisons) |
| Janelidze et al. (2022)^18^ | DS = 300  NC = 37 | Plasma | SIMOA, ECL | A$\beta_{42}$, A$\beta_{40}$, t-tau, p-tau 217, NfL, GFAP | Cross-sectional | ABC-DS | Included |
| Carmona-Iragui et al. (2021)^19^ | DS = 236 | Plasma | SIMOA | NfL | Cross-sectional, Longitudinal | Multicentre from France, Germany, Spain, UK, USA | Included |

(Continues)

| Study | Sample numbers | Blood fraction | Platform | Biomarkers | Study design | Cohort original | Meta-analysis |
| --- | --- | --- | --- | --- | --- | --- | --- |
| Mengel et al. (2020)^20^ | DS = 100  NC = 100 | Plasma | SIMOA | A$\beta_{42}$, NT1 tau, NfL | Longitudinal | MGH, LonDownS | Included |
| Moreau et al. (2022)^21^ | DS = 156  NC = 19 | Plasma | solid phase immobilized epitope immunoassay, sandwich ELISA | DYRK1A, ADNP | Cross-sectional | Hospital de la Santa Creu I Sant Pau, Barcelona; Institute Jérôme Lejeune, Paris | Excluded (non-SIMOA platform) |
| Raha-Chowdhury et al. (2018)^22^ | DS = 50  NC = 50 | Whole blood, serum | Western blotting | sTREM2 | Cross-sectional | Cambridge Brain Bank | Excluded (non-SIMOA platform) |
| Mgaieth et al. (2023)^23^ | DS = 302 | Plasma | SIMOA | NfL, GFAP | Longitudinal | LonDownS | Included |
| Yang et al. (2020)^24^ | DS = 46 | Plasma | ELISA | A$\beta_{42}$, A$\beta_{40}$ | Cross-sectional | Chongqing Medical University | Excluded (non-SIMOA platform) |
| Raha et al. (2021)^25^ | DS = 47  NC = 50 | Serum | ELISA | IL-6 | Cross-sectional | Cambridge | Excluded (non-SIMOA platform) |
| Koenig et al. (2021)^26^ | DS = 11  NC = 11 | Plasma | Luminex | A$\beta_{42}$, A$\beta_{40}$, t-tau, sTREM2, cytokines | Cross-sectional | Cleveland Clinic | Excluded (non-SIMOA platform) |
| Araya et al. (2022)^27^ | DS = 316  NC = 103 | Plasma | SIMOA, SOMAscan | TNF-α, NfL, t-tau, GFAP | Cross-sectional | Crnic Institute Human Trisome Project | Excluded (not reported required data) |
| Raha-Chowdhury et al. (2021)^28^ | DS = 47  NC = 50 | Serum | ELISA, Western blotting | IL-6, TREM2 | Cross-sectional | Cambridge | Excluded (non-SIMOA platform, not plasma) |

(Continues)

| Study | Sample numbers | Blood fraction | Platform | Biomarkers | Study design | Cohort original | Meta-analysis |
| --- | --- | --- | --- | --- | --- | --- | --- |
| Kasai et al. (2017)^29^ | DS = 21  NC = 22 | Plasma | SIMOA | t-tau | Cross-sectional | Kyoto Prefectural University of Medicine, Hananoki Medical Welfare Center | Included |
| Delabar et al. (2023)^30^ | DS = 38  DS-AD = 32 | Plasma | Immunometric assay (Meso Scale Diagnostics) | DYRK1A | Cross-sectional | Kentucky cohort | Excluded (non-SIMOA platform) |
| Manti et al. (2018)^31^ | DS = 24  NC = 12 | Serum | ELISA, Nephelometry | CRP, SAA, HMGB1 | Cross-sectional | University Hospital of Messina, Italy | Excluded (non-SIMOA platform, not plasma) |
| Grasso et al. (2024)^32^ | DS = 49  NC = 44 | Plasma | ELISA | TGF-β1, TNF-α | Prospective observational | Oasi Research Institute-IRCCS, Troina, Italy | Excluded (non-SIMOA platform) |
| Dekker et al. (2018)^33^ | DS = 241 | Plasma | RP-HPLC | Noradrenaline, MHPG, Dopamine, Serotonin | Cross-sectional | DABNI | Excluded (non-SIMOA platform) |
| Conti et al. (2017)^34^ | DS = 5 | Plasma | ELISA | A$\beta_{42}$ | Cross-sectional | S. Gerardo Hospital, Monza, Italy | Excluded (non-SIMOA platform) |
| Pentz et al. (2021)^35^ | DS = 36  NC = 16 | Plasma | Western Blot, ELISA | proNGF (27 kDa, 50 kDa), MMP-3, tPA | Cross-sectional | Sant Pau Memory Unit and Barcelona Down Medical Center | Excluded (non-SIMOA platform) |

(Continues)

| Study | Sample numbers | Blood fraction | Platform | Biomarkers | Study design | Cohort original | Meta-analysis |
| --- | --- | --- | --- | --- | --- | --- | --- |
| Strydom et al. (2018)^36^ | DS = 100 | Plasma | SIMOA | NfL | Cross-sectional, Longitudinal | LonDownS | Included |
| DiProspero et al. (2024)^37^ | DS = 101 | Plasma | SIMOA | NfL | Cross-sectional | ADDS | Excluded (not reported required data) |
| Lleó et al. (2021)^38^ | DS = 366  NC = 44 | Plasma | SIMOA | p-tau 181, NfL | Cross-sectional | DABNI, SPIN | Included |
| Montoliu-Gaya et al. (2023)^39^ | DS = 585  NC = 351 | Plasma | SIMOA | p-tau 181, NfL, GFAP | Longitudinal | DABNI, SPIN, Alzheimer-21 | Included |
| Fortea et al. (2018)^40^ | DS = 282  NC = 67 | Plasma | SIMOA | A$\beta_{42}$, A$\beta_{40}$, t-tau, NfL | Cross-sectional | DABNI, SPIN | Included |
| Startin et al. (2019)^41^ | DS = 31  NC = 27 | Plasma | SIMOA | A$\beta_{42}$, A$\beta_{40}$, t-tau, NfL, IL1β, IL10, IL6, TNFα | Cross-sectional | LonDownS, EMIF | Included |
| Rafii et al. (2019)^42^ | DS = 12 | Plasma | SIMOA | NfL | Cross-sectional | DSBI | Excluded (no comparisons) |
| Shinomoto et al. (2019)^43^ | DS = 24  NC = 24 | Plasma | SIMOA | NfL | Cross-sectional | Kyoto Prefectural University of Medicine | Included |
| Stern et al. (2023)^44^ | DS = 239 | Plasma | SIMOA | NT1-tau, A$\beta_{42}$, A$\beta_{40}$, A$\beta_{37}$ | Cross-sectional, Longitudinal | ABC-DS, UKY | Excluded (not reported required data) |
| Petersen et al. (2021)^45^ | DS = 305 | Plasma | SIMOA | t-tau, NfL | Cross-sectional | ABC-DS | Included |

(Continues)

| Study | Sample numbers | Blood fraction | Platform | Biomarkers | Study design | Cohort original | Meta-analysis |
| --- | --- | --- | --- | --- | --- | --- | --- |
| Moni et al. (2022)^46^ | DS = 115 | Plasma | ECL | A$\beta_{42}$, A$\beta_{40}$, t-tau, NfL, IL-10, IL-6, TNF-α, CRP | Cross-sectional | ADDS | Excluded (non-SIMOA platform) |
| Petersen et al. (2020)^47^ | DS = 305 | Plasma | Meso Scale Discovery | IL-10, IL-18, CRP, SAA, FABP3, TNF-α | Cross-sectional | ABC-DS | Excluded (non-SIMOA platform) |
| O'Bryant et al. (2020)^48^ | DS = 398 | Plasma | ECL | IL-6, CRP, sICAM1, I309, PPY, SAA, TPO, IL-10, TARC, IL-5, FABP3 | Cross-sectional | New York State Institute for Basic Research | Excluded (non-SIMOA platform) |
| Petersen et al. (2020)^49^ | DS = 398 | Plasma | ECL | FABP3, B2M, PPY, CRP, ICAM-1, TPO, IL-5, IL-6, IL-7, IL-10, IL-18, TNF-α, TARC | Cross-sectional | New York State Institute for Basic Research | Excluded (non-SIMOA platform) |
| Tatebe et al. (2017)^50^ | DS = 20  NC = 15 | Plasma | SIMOA | p-tau 181 | exploratory pilot study | Kyoto Prefectural University of Medicine, Japan | Included |
| Oeckl et al. (2022)^51^ | DS = 61  NC = 23 | Plasma, Serum | SIMOA, IPM | Beta-synuclein, p-tau181 | Case-control | University of Munich and Ulm University Hospital | Excluded (not plasma, not reported required data) |
| Pape et al. (2021)^52^ | DS = 54 | Plasma | SIMOA | NfL | Cross-sectional | LonDownS | Excluded (participants overlapping with Mgaieth et al. (2023)^23^) |

(Continues)

| Study | Sample numbers | Blood fraction | Platform | Biomarkers | Study design | Cohort original | Meta-analysis |
| --- | --- | --- | --- | --- | --- | --- | --- |
| Sánchez-Moreno et al. (2024)^53^ | DS = 120 | Plasma | SIMOA | p-tau 181, NfL | Cross-sectional | Hospital Universitario de La Princesa, Madrid | Excluded (not reported required data) |
| Huggard et al. (2020)^54^ | DS = 114  NC = 60 | Plasma | ELISA | IL-2, IL-6, IL-8, IL-10, IL-18, IL-1β, TNF-α, IFN-γ, EPO, VEGF, GM-CSF, IL-1ra | Cross-sectional | Children’s Health Ireland, Dublin | Excluded (non-SIMOA platform) |
| Tarani et al. (2020)^55^ | DS = 9  NC = 21 | Serum | ELISA | TNF-α, TGF-β, MCP-1, IL-1α, IL-2, IL-6, IL-10, IL-12, NGF, BDNF | Cross-sectional | Sapienza University Hospital, Rome | Excluded (not plasma, non-SIMOA platform) |
| Powers et al. (2019)^56^ | DS = 75 | Plasma | UHPLC-HRMS | Kynurenine, Quinolinic acid | Cross-sectional | Crnic Institute’s Human Trisome Project, Translational Nexus Clinical Data Registry | Excluded (non-SIMOA platform) |
| Edwards et al. (2024)^57^ | DS = 185 | Plasma | SIMOA | A$\beta_{42}$, A$\beta_{40}$, p-tau 217, NfL, GFAP | Cross-sectional | ABC-DS | Excluded (not reported required data) |
| Hamlett et al. (2017)^58^ | DS = 47  NC = 37 | Neuronal exosomes | ELISA | A$\beta_{42}$, p-tau 181, p-tau 396 | Cross-sectional | Various clinics in the US and Spain | Excluded (non-SIMOA platform) |

Abbreviations: AD = Alzheimer's disease; DS = Down syndrome; NC = normal controls; DS_NAD = Down syndrome without Alzheimer’s disease; DS_CS = Down syndrome with cognitively stable; DS_pAD = Down syndrome with prodromal Alzheimer’s disease; DS_AD = Down syndrome with Alzheimer’s disease; SD = standard deviation; Aβ = Amyloid β; total tau = Total Tau protein; p-tau 181 = Phosphorylated tau 181; p-tau217 = Phosphorylated tau 217, NT1-tau = N-terminal tau fragment; p-S396-tau = Phosphorylated tau S396; NfL = Neurofilament light; GFAP = Glial fibrillary acidic protein; SIMOA = Single Molecule Array; IMR = Immunomagnetic Reduction; ECL = Electrochemiluminescence; ELISA = Enzyme-Linked Immunosorbent Assay; IP-MS = Immunoprecipitation-Mass Spectrometry; LEN = Latex-Enhanced Nephelometry; TREM2 = Triggering receptor expressed on myeloid cells 2; HMGB1 = High mobility group box 1; MMP-3 = matrix metalloproteinase-3; tPA = Tissue plasminogen activator; IL-6 = Interleukin-6; sTREM2 = soluble TREM; IL-10 = interleukin-10; TNFα = tumor necrosis factor-alpha; TGF-β1 = transforming growth factor-beta 1; IL-2 = interleukin-2; IL-1α = interleukin-1 alpha; IL-12 = interleukin-12; MCP-1 = monocyte chemoattractant protein-1; IL-1ra = interleukin-1 receptor antagonist; GM-CS = granulocyte-macrophage colony-stimulating; IL-1β = interleukin-1 beta; Epo = erythropoietin; TCC = terminal complement; iC3b = inactivated complement component 3b; C1q = complement component 1q; C9 = complement component 9; C3 = complement component 3 (C3); FHR4 = factor H-related protein 4; sCR1 = soluble complement receptor 1; DYRK1A = dual-specificity tyrosine-phosphorylation-regulated kinase 1A; ADNP = activity-dependent neuroprotective protein; NGF = Neurotrophic factors like nerve growth factor; BDNF = brain-derived neurotrophic factor; C-RP = C-reactive protein; SAA = serum amyloid A; VEGF = vascular endothelial growth factor; APL1β25 = Amyloid Precursor-Like Protein 1 Beta 25; APL1β27 = Amyloid Precursor-Like Protein 1 Beta 27; APL1β28 = Amyloid Precursor-Like Protein 1 Beta 28; MHPG = 3-Methoxy-4-Hydroxyphenylglycol; APP = Amyloid Precursor Protein; RP-HPLC = reversed phase high-performance liquid chromatography.

## Quality Assessment of Studies Included in the Meta-Analysis

Table S6 Quality Assessment of Studies Included in the Meta-Analysis

## Forest and Funnel Plot in the Meta-Analysis

Figure S1 Meta-analysis of studies comparing plasma **Aβ42** levels of individuals with **DS** and **normal controls**. Abbreviations: DS = Down syndrome, AD = Alzheimer's disease, SD = standard deviation, CI = Confidence Interval, Std. = Standardized


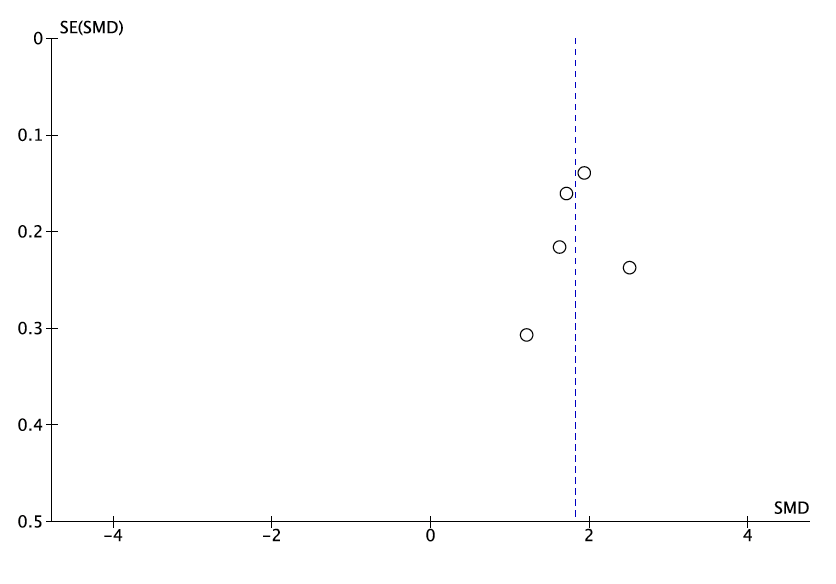

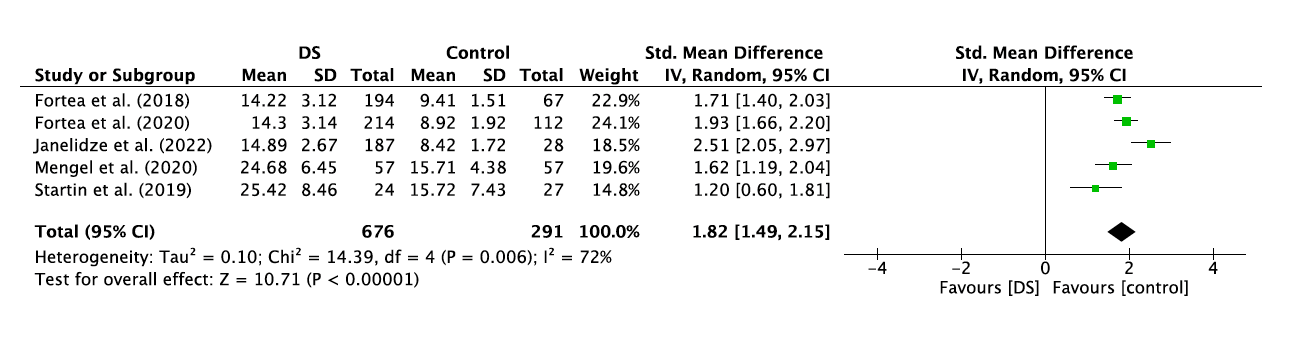


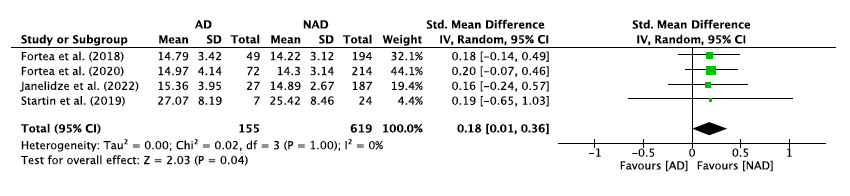
Figure S2 Meta-analysis of studies comparing plasma **Aβ42** levels of individuals with **DS with AD** and **without AD**. Abbreviations: DS = Down syndrome, AD = Alzheimer's disease, SD = standard deviation, CI = Confidence Interval, Std. = Standardized


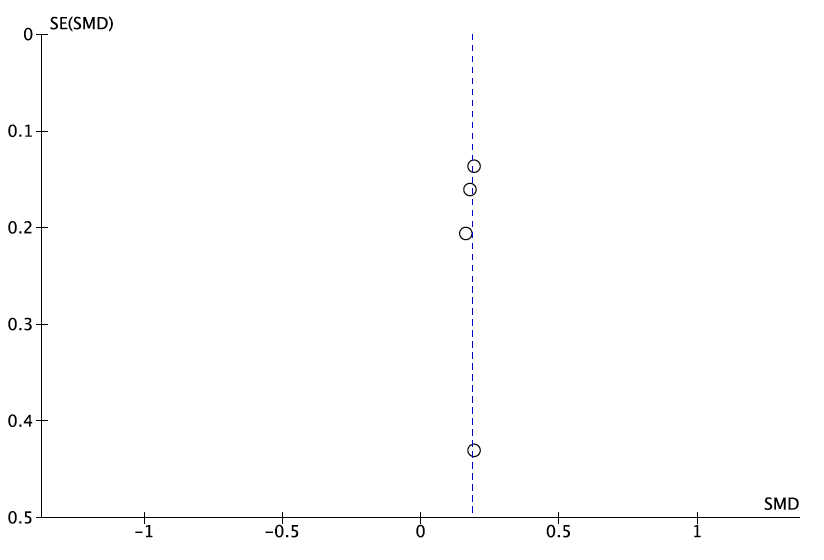


Figure S3 Meta-analysis of studies comparing plasma **Aβ42** levels of individuals with **DS with prodromal AD** and **cognitively stable**. Abbreviations: DS = Down syndrome, AD = Alzheimer's disease, SD = standard deviation, CI = Confidence Interval, Std. = Standardized


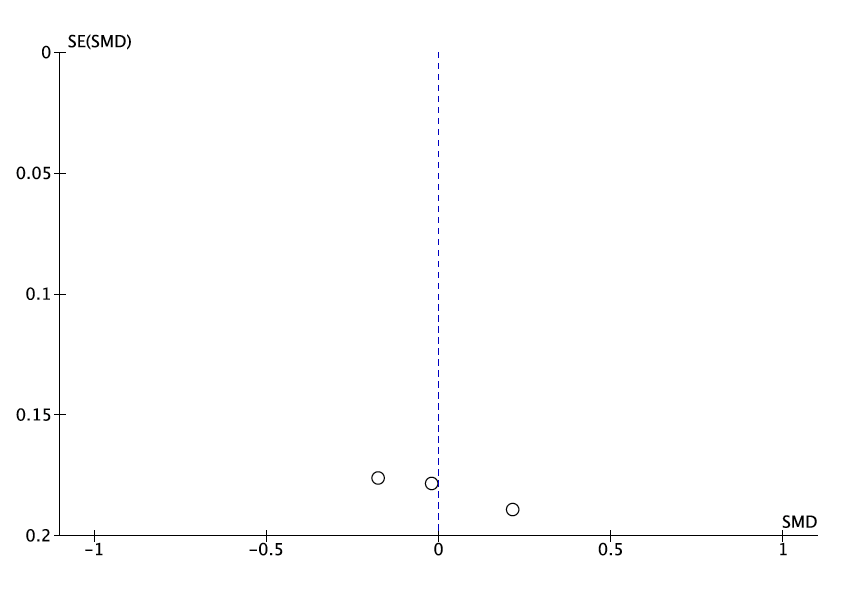

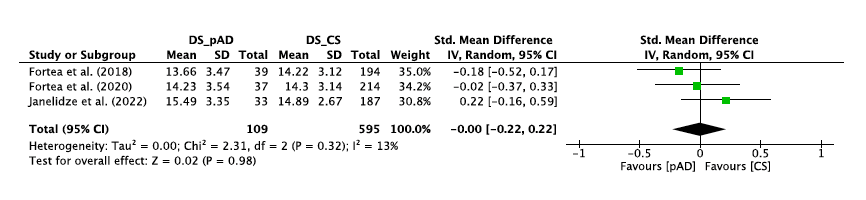


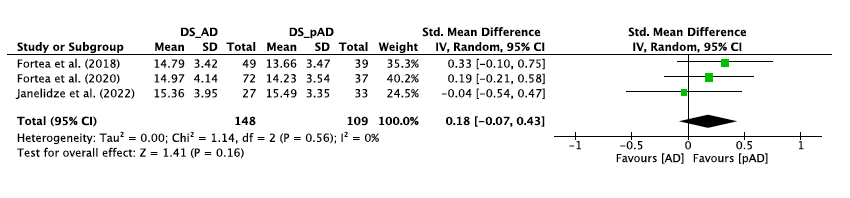
Figure S4 Meta-analysis of studies comparing plasma **Aβ42** levels of individuals with **DS with AD** and **prodromal AD**. Abbreviations: DS = Down syndrome, AD = Alzheimer's disease, SD = standard deviation, CI = Confidence Interval, Std. = Standardized


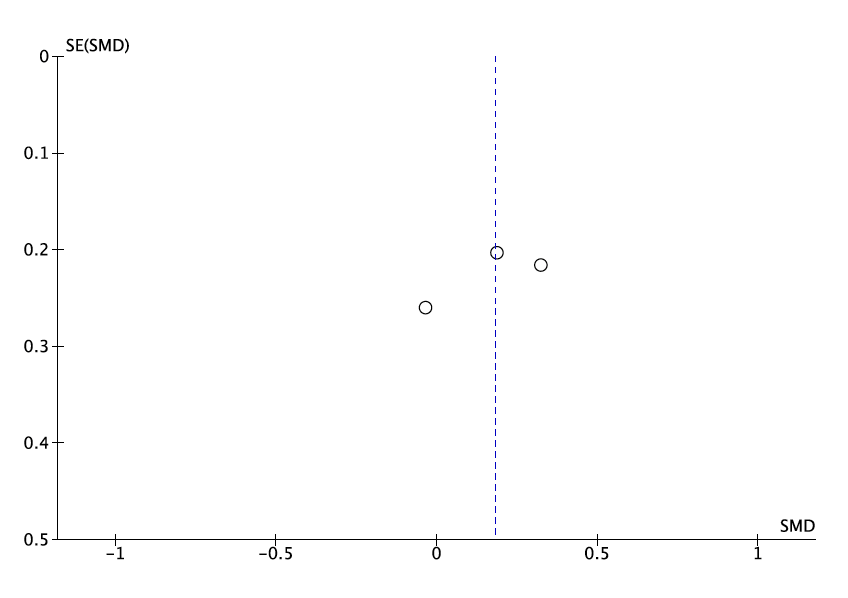


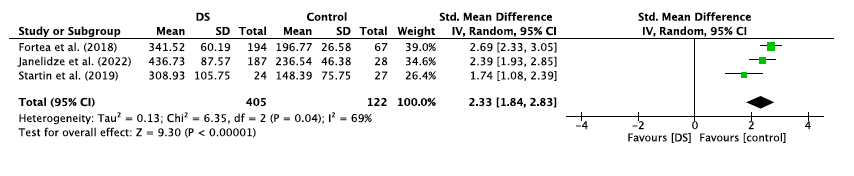
Figure S5 Meta-analysis of studies comparing plasma **Aβ40** levels of individuals with **DS** and **normal controls**. Abbreviations: DS = Down syndrome, AD = Alzheimer's disease, SD = standard deviation, CI = Confidence Interval, Std. = Standardized


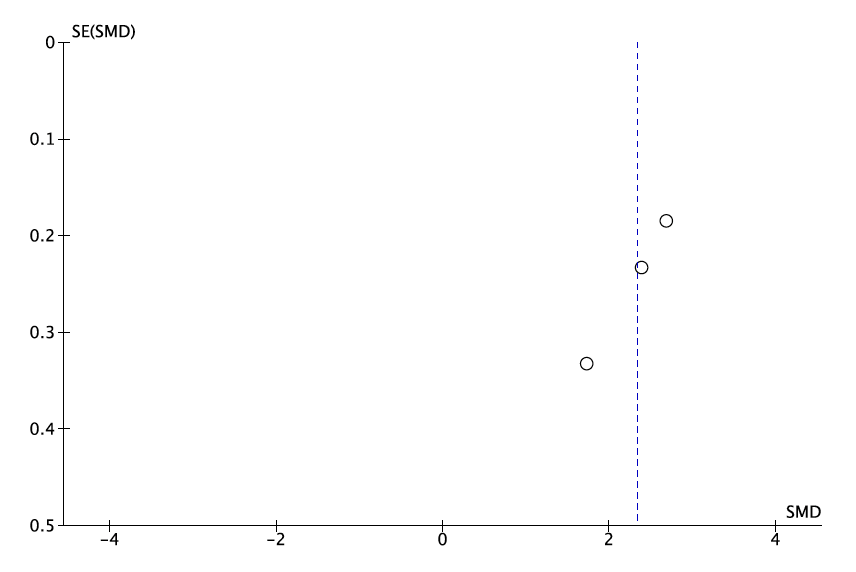


Figure S6 Meta-analysis of studies comparing plasma **Aβ40** levels of individuals with **DS with AD** and **without AD**. Abbreviations: DS = Down syndrome, AD = Alzheimer's disease, SD = standard deviation, CI = Confidence Interval, Std. = Standardized


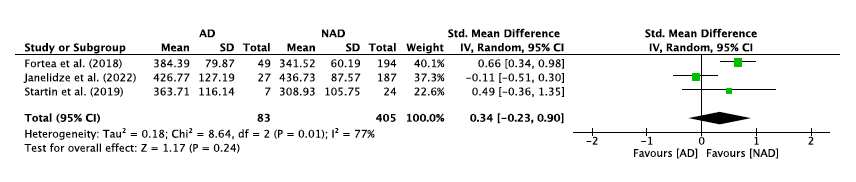


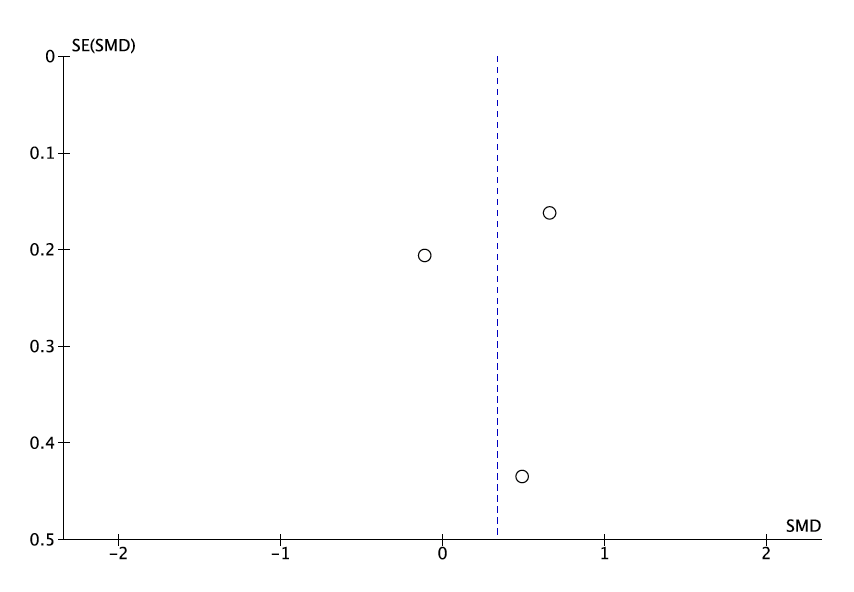


Figure S7 Meta-analysis of studies comparing plasma **Aβ40** levels of individuals with **DS with prodromal AD** and **cognitively stable**. Abbreviations: DS = Down syndrome, AD = Alzheimer's disease, SD = standard deviation, CI = Confidence Interval, Std. = Standardized


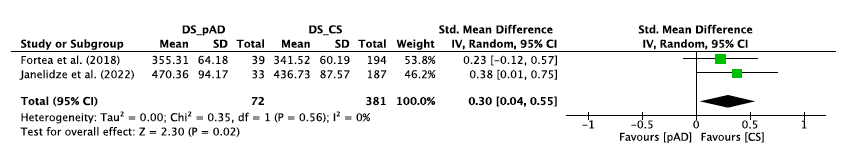


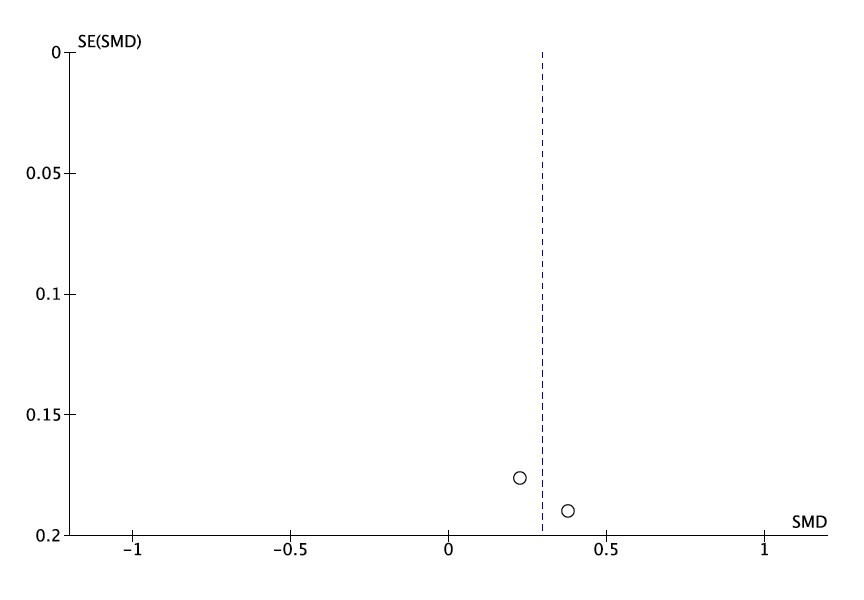


Figure S8 Meta-analysis of studies comparing plasma **Aβ40** levels of individuals with **DS with AD** and **prodromal AD**. Abbreviations: DS = Down syndrome, AD = Alzheimer's disease, SD = standard deviation, CI = Confidence Interval, Std. = Standardized


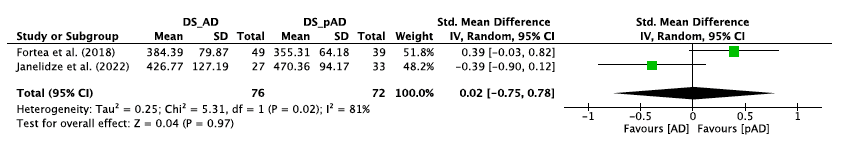


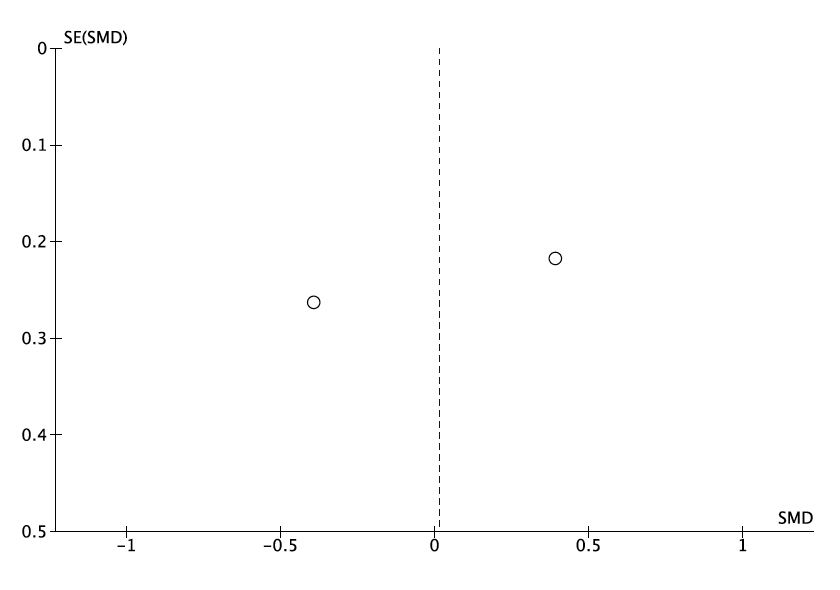


Figure S9 Meta-analysis of studies comparing plasma **Aβ42/40** ratio of individuals with **DS** and **normal controls**. Abbreviations: DS = Down syndrome, AD = Alzheimer's disease, SD = standard deviation, CI = Confidence Interval, Std. = Standardized


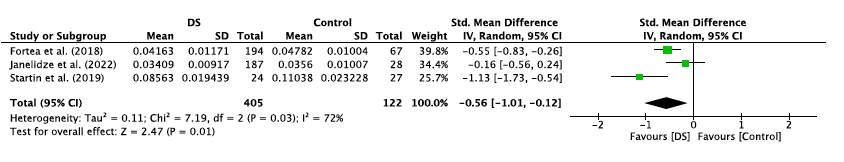


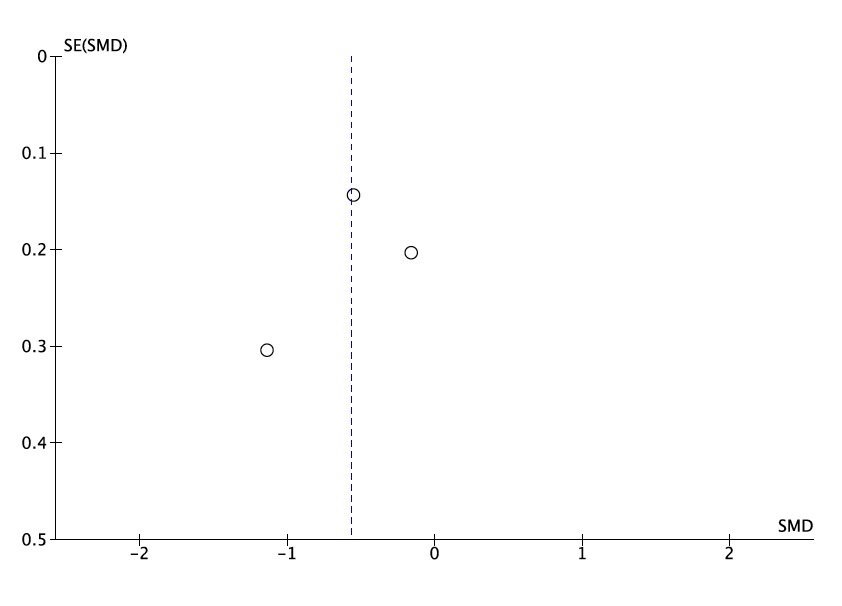


Figure S10 Meta-analysis of studies comparing plasma **Aβ42/40** ratio of individuals with **DS with AD** and **without AD**. Abbreviations: DS = Down syndrome, AD = Alzheimer's disease, SD = standard deviation, CI = Confidence Interval, Std. = Standardized


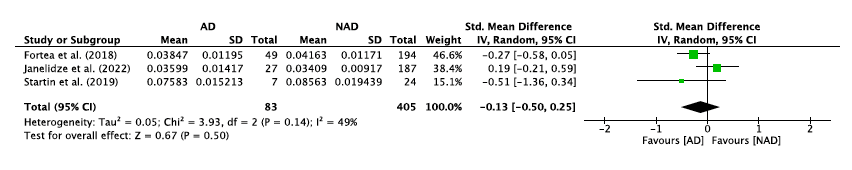


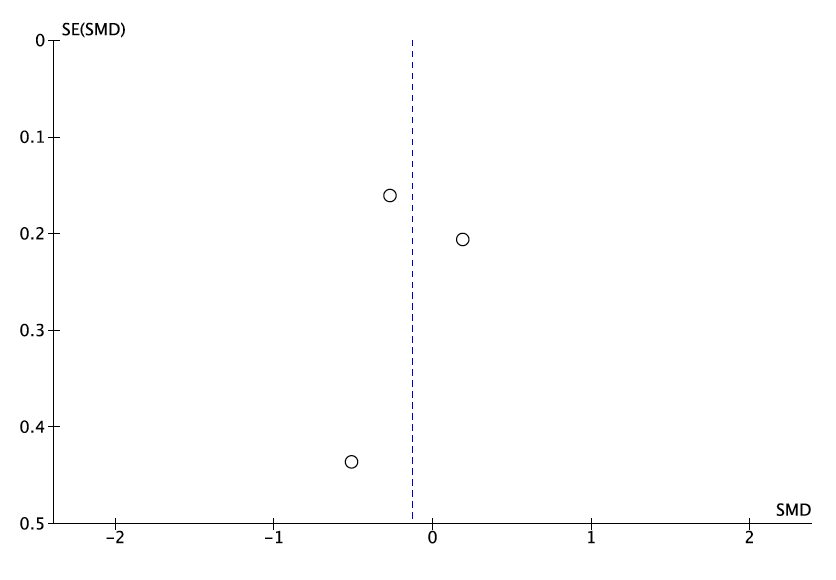


Figure S11 Meta-analysis of studies comparing plasma **Aβ42/40** ratio of individuals with **DS with prodromal AD** and **cognitively stable**. Abbreviations: DS = Down syndrome, AD = Alzheimer's disease, SD = standard deviation, CI = Confidence Interval, Std. = Standardized


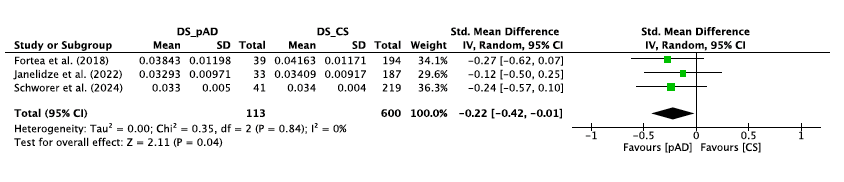


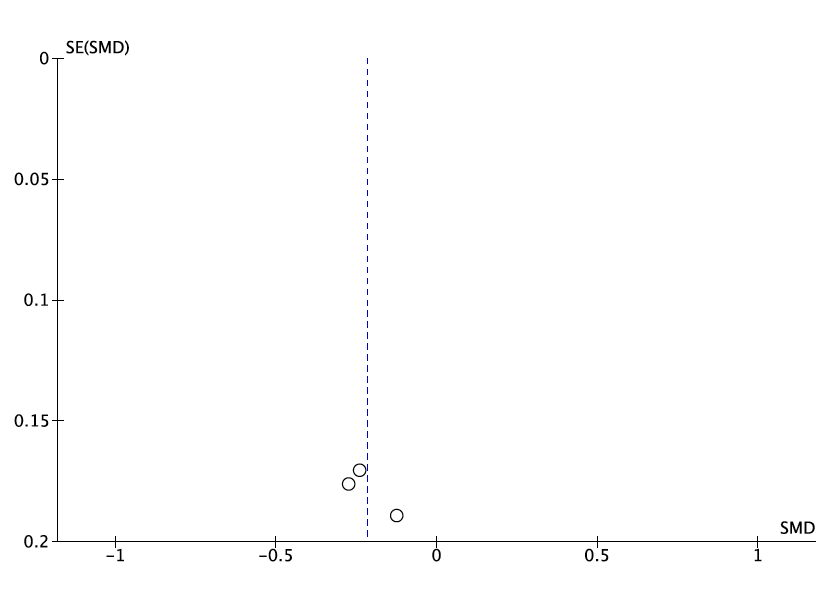


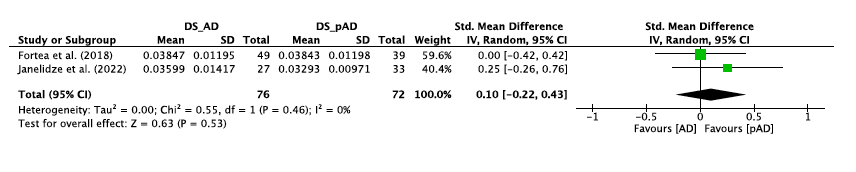
Figure S12 Meta-analysis of studies comparing plasma **Aβ42/40** ratio of individuals with **DS with AD** and **prodromal AD**. Abbreviations: DS = Down syndrome, AD = Alzheimer's disease, SD = standard deviation, CI = Confidence Interval, Std. = Standardized


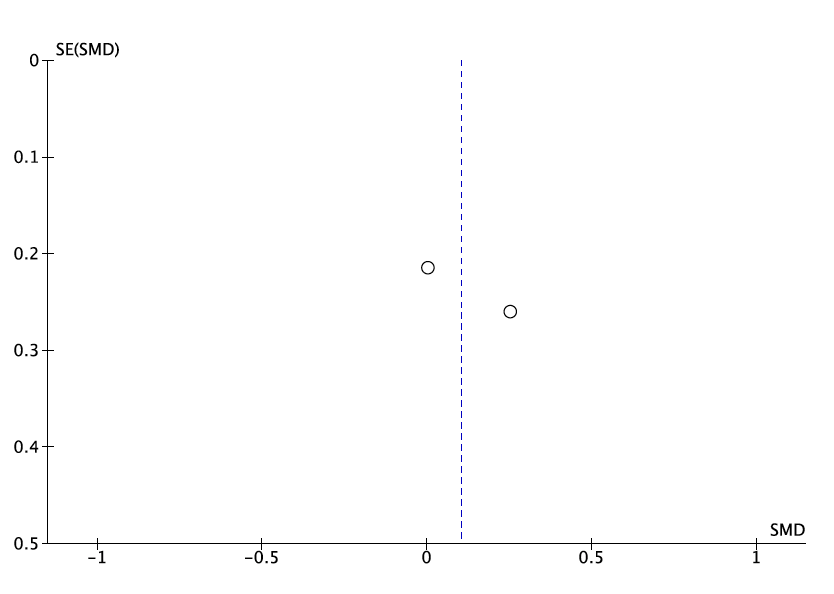


Figure S13 Meta-analysis of studies comparing plasma **p-tau 181** levels of individuals with **DS** and **normal controls**. Abbreviations: DS = Down syndrome, AD = Alzheimer's disease, SD = standard deviation, CI = Confidence Interval, Std. = Standardized


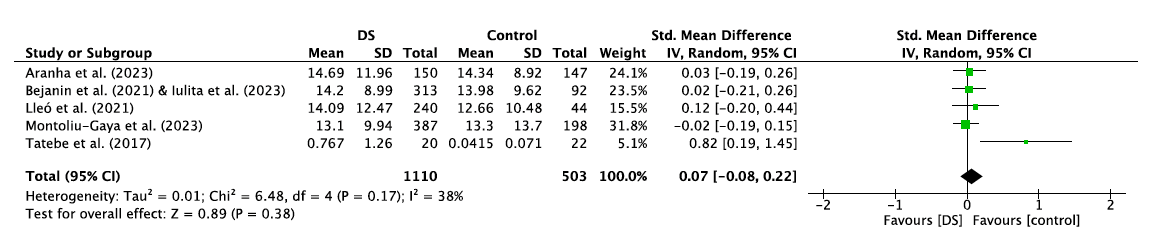


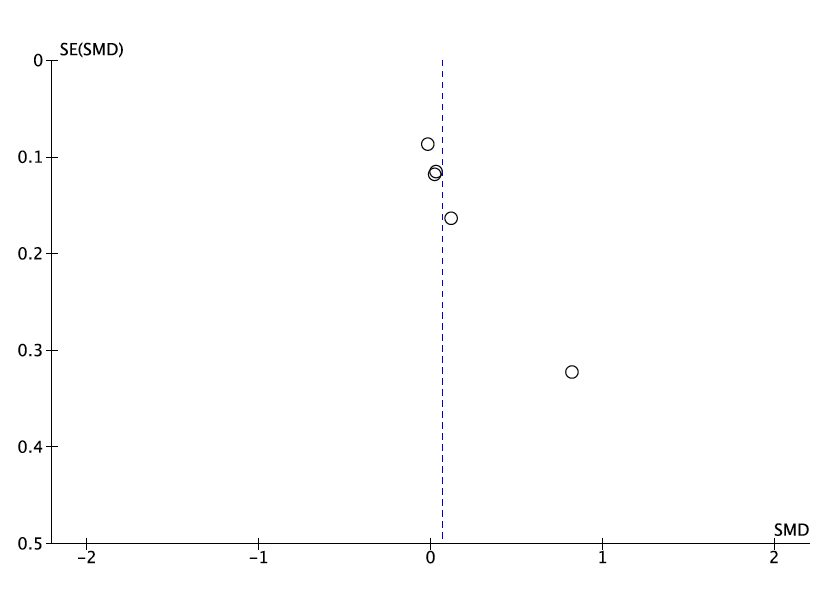


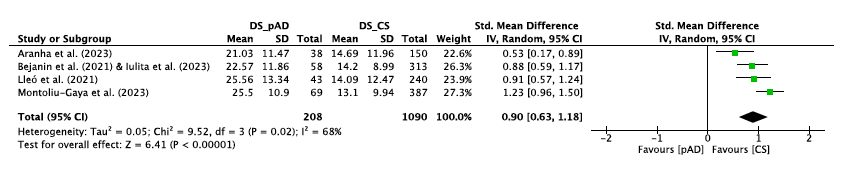
Figure S14 Meta-analysis of studies comparing plasma **p-tau 181** levels of individuals with **DS with AD** and **without AD**. Abbreviations: DS = Down syndrome, AD = Alzheimer's disease, SD = standard deviation, CI = Confidence Interval, Std. = Standardized


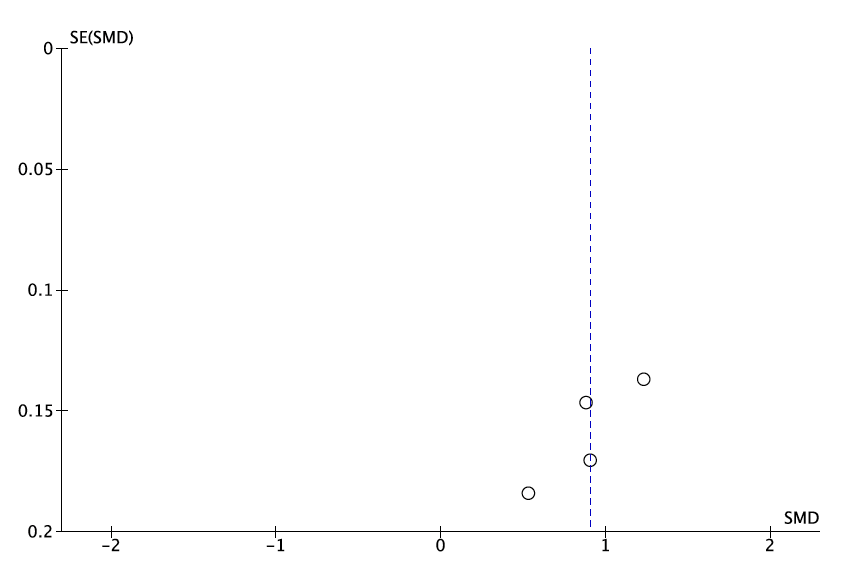


Figure S15 Meta-analysis of studies comparing plasma **p-tau 181** levels of individuals with **DS with prodromal AD** and **cognitively stable**. Abbreviations: DS = Down syndrome, AD = Alzheimer's disease, SD = standard deviation, CI = Confidence Interval, Std. = Standardized


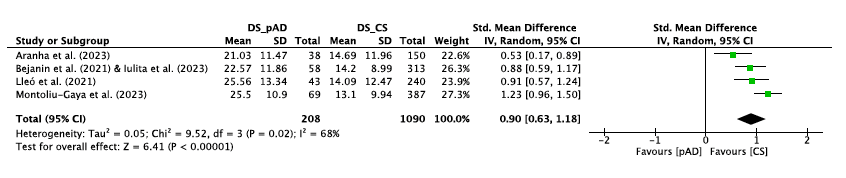


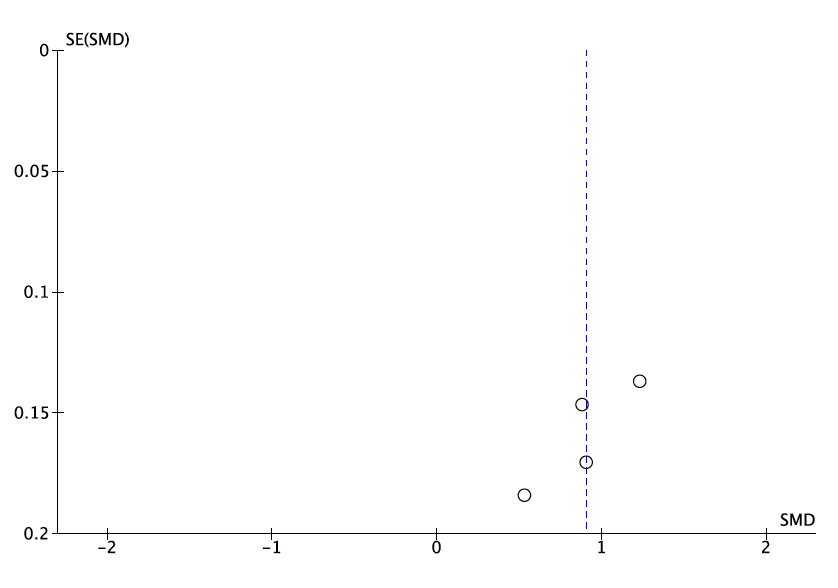


Figure S16 Meta-analysis of studies comparing plasma **p-tau 181** levels of individuals with **DS with AD and prodromal AD**. Abbreviations: DS = Down syndrome, AD = Alzheimer's disease, SD = standard deviation, CI = Confidence Interval, Std. = Standardized


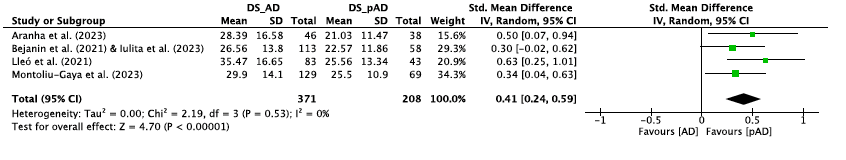


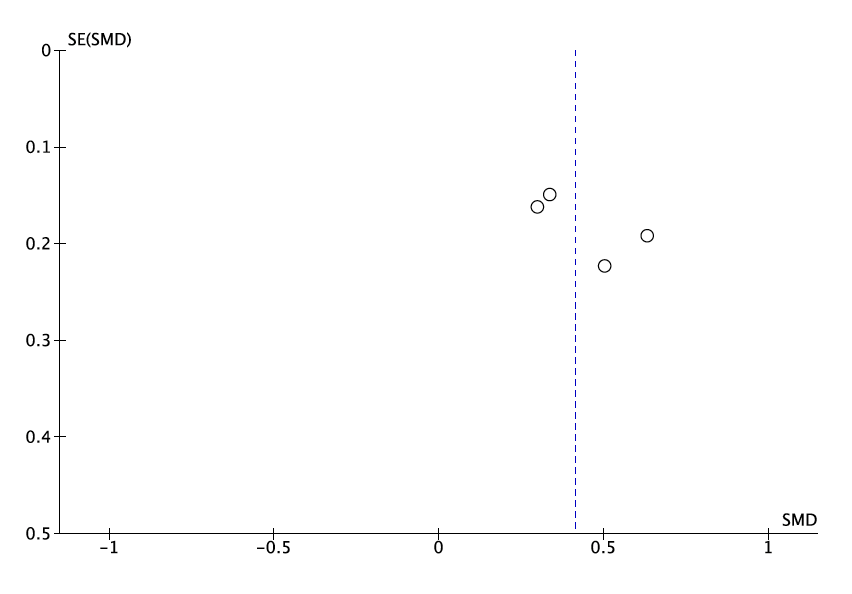


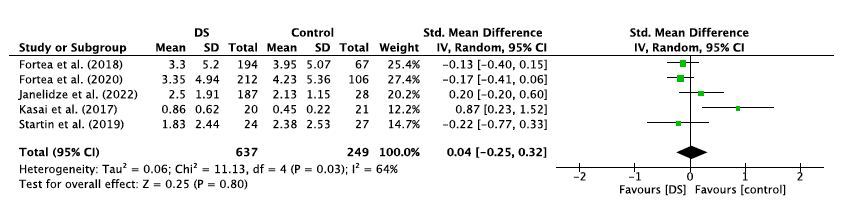
Figure S17 Meta-analysis of studies comparing plasma **t-tau** levels of individuals with **DS** and **normal controls**. Abbreviations: DS = Down syndrome, AD = Alzheimer's disease, SD = standard deviation, CI = Confidence Interval, Std. = Standardized


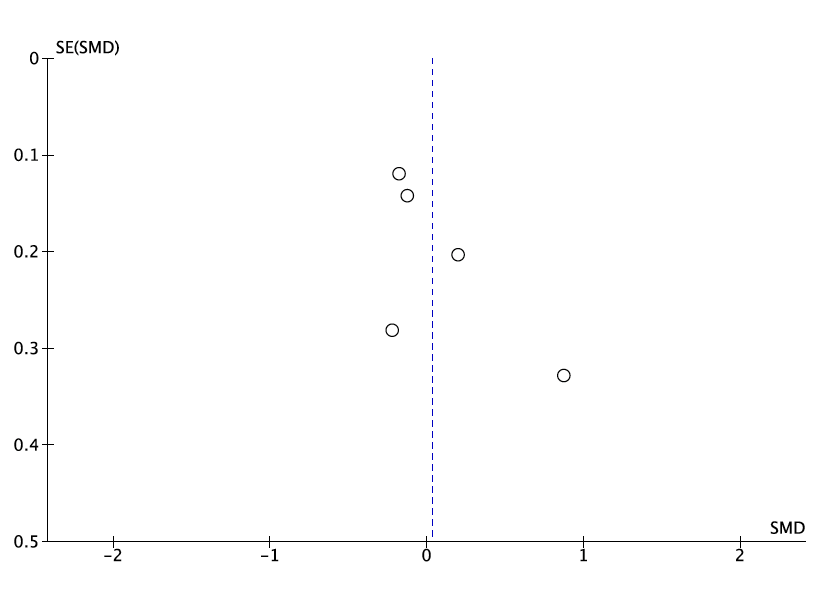


Figure S18 Meta-analysis of studies comparing plasma **t-tau** levels of individuals with **DS** **with AD and without AD**. Abbreviations: DS = Down syndrome, AD = Alzheimer's disease, SD = standard deviation, CI = Confidence Interval, Std. = Standardized


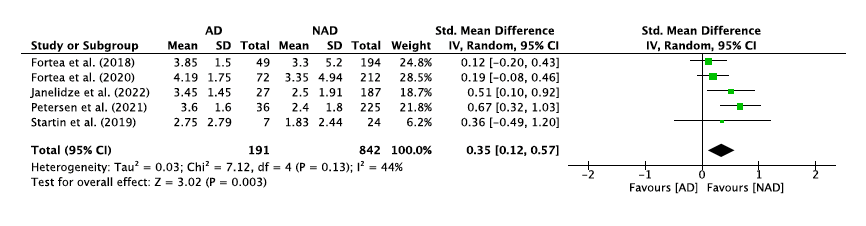


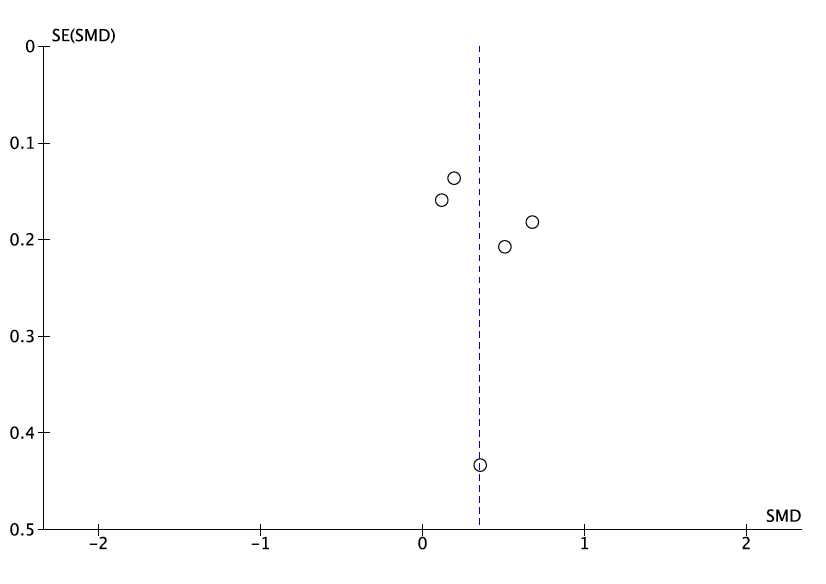


Figure S19 Meta-analysis of studies comparing plasma **t-tau** levels of individuals with **DS** **with prodromal AD and cognitively stable**. Abbreviations: DS = Down syndrome, AD = Alzheimer's disease, SD = standard deviation, CI = Confidence Interval, Std. = Standardized


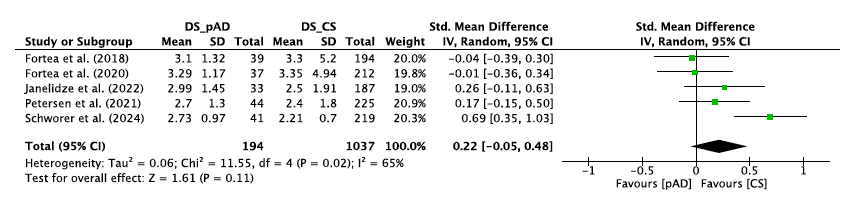


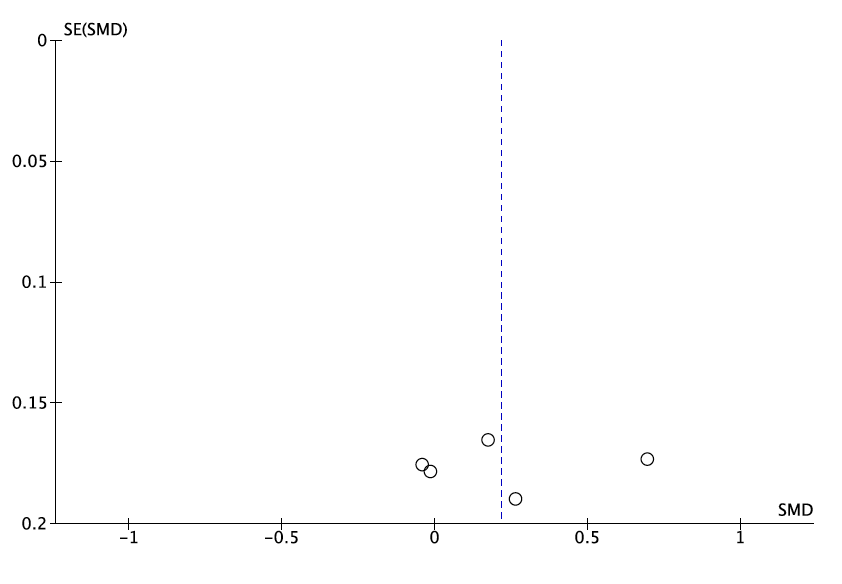


Figure S20 Meta-analysis of studies comparing plasma **t-tau** levels of individuals with **DS** **with AD and prodromal AD**. Abbreviations: DS = Down syndrome, AD = Alzheimer's disease, SD = standard deviation, CI = Confidence Interval, Std. = Standardized


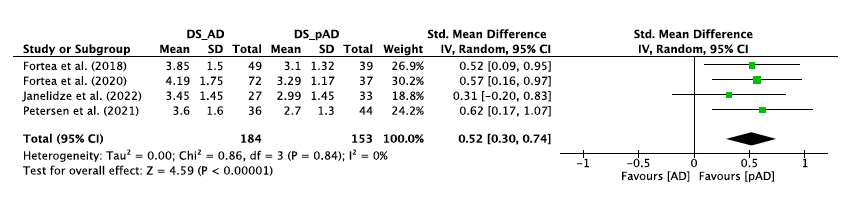


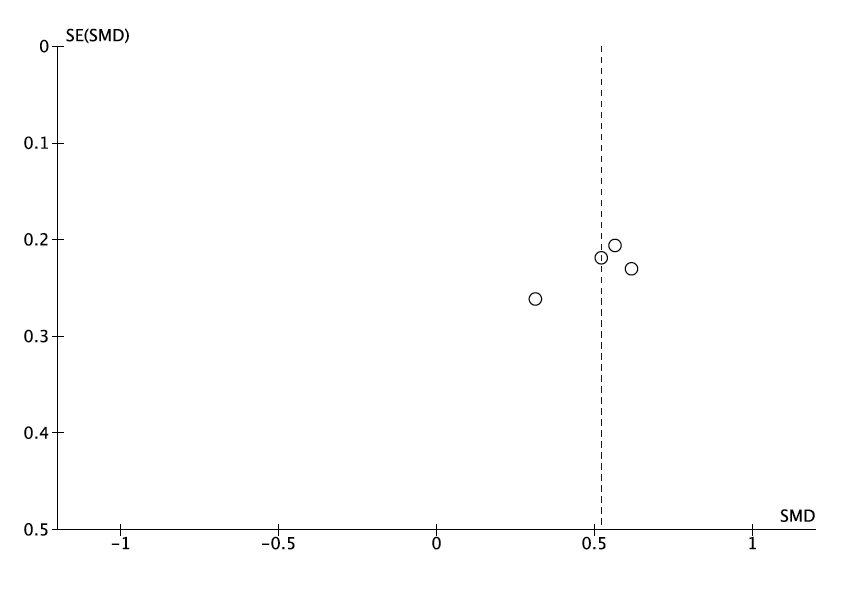


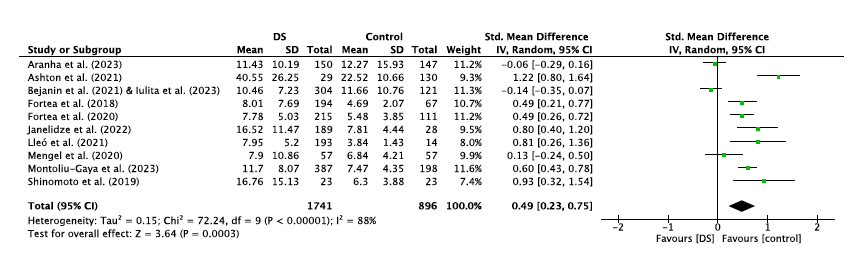
Figure S21 Meta-analysis of studies comparing plasma **NfL** levels of individuals with **DS** and **normal controls**. Abbreviations: DS = Down syndrome, AD = Alzheimer's disease, SD = standard deviation, CI = Confidence Interval, Std. = Standardized


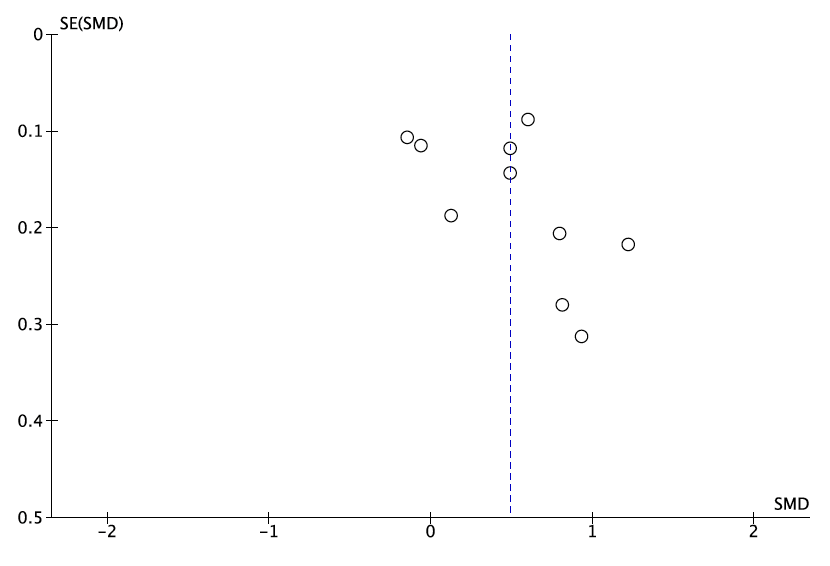


Figure S22 Meta-analysis of studies comparing plasma **NfL** levels of individuals with **DS** **with AD and without AD**. Abbreviations: DS = Down syndrome, AD = Alzheimer's disease, SD = standard deviation, CI = Confidence Interval, Std. = Standardized


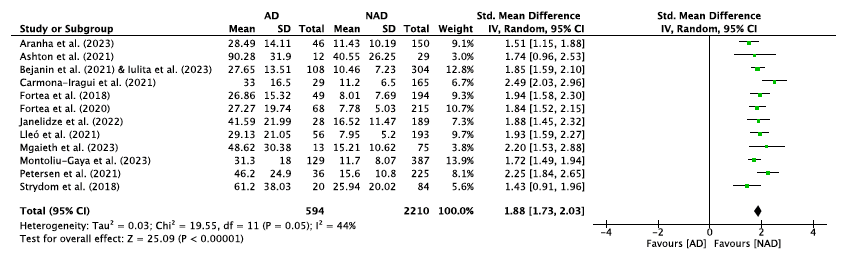


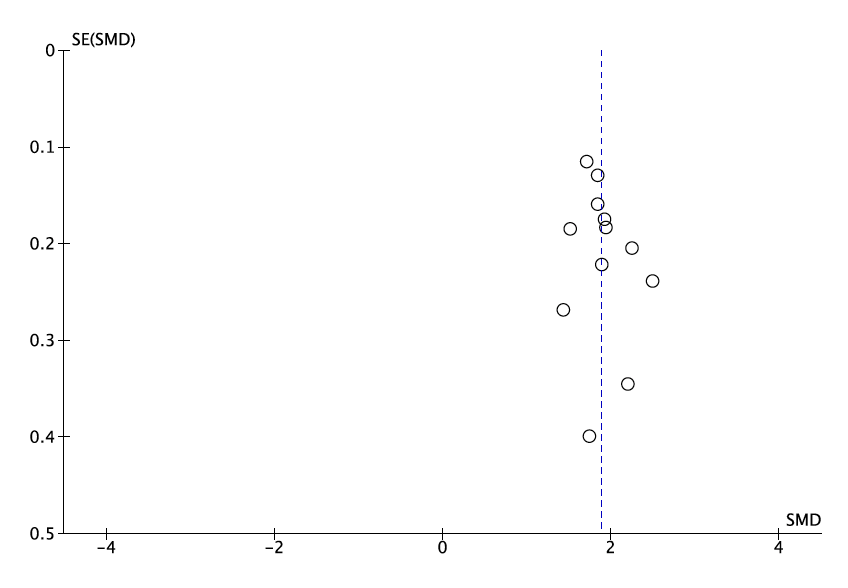


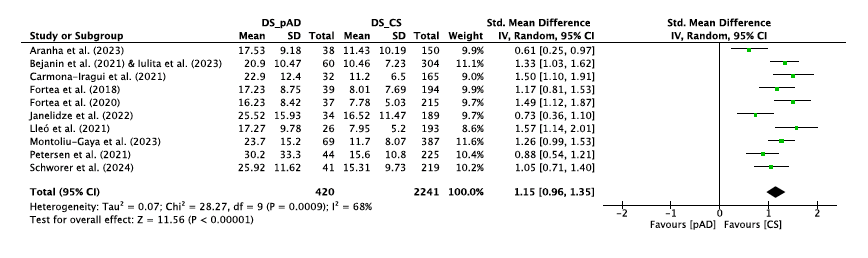
Figure S23 Meta-analysis of studies comparing plasma **NfL** levels of individuals with **DS** **with prodromal AD and cognitively stable**. Abbreviations: DS = Down syndrome, AD = Alzheimer's disease, SD = standard deviation, CI = Confidence Interval, Std. = Standardized


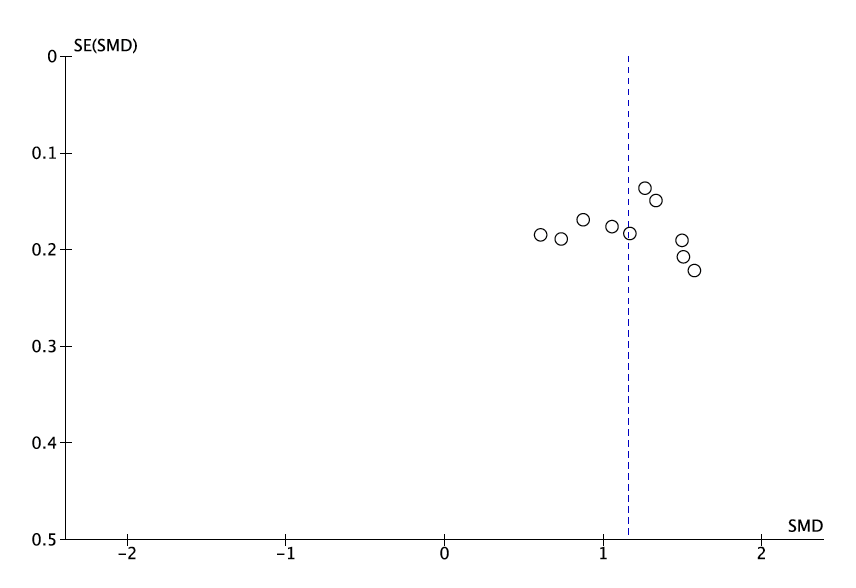


Figure S24 Meta-analysis of studies comparing plasma **NfL** levels of individuals with **DS** **with AD and prodromal AD**. Abbreviations: DS = Down syndrome, AD = Alzheimer's disease, SD = standard deviation, CI = Confidence Interval, Std. = Standardized


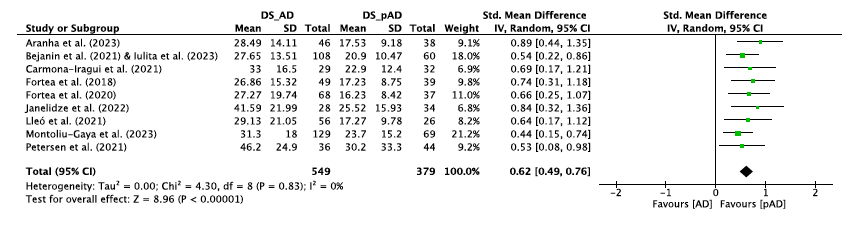


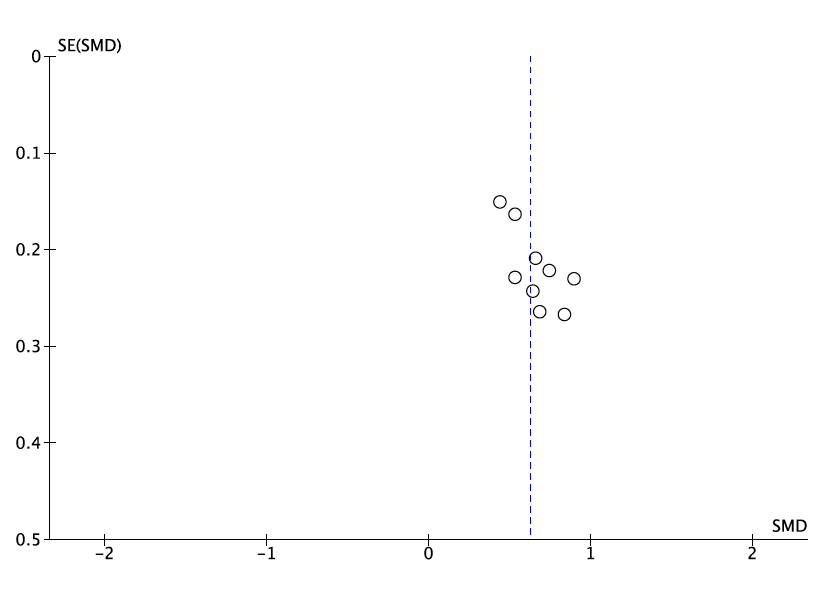


Figure S25 Meta-analysis of studies comparing plasma **GFAP** levels of individuals with **DS** and **normal controls**. Abbreviations: DS = Down syndrome, AD = Alzheimer's disease, SD = standard deviation, CI = Confidence Interval, Std. = Standardized


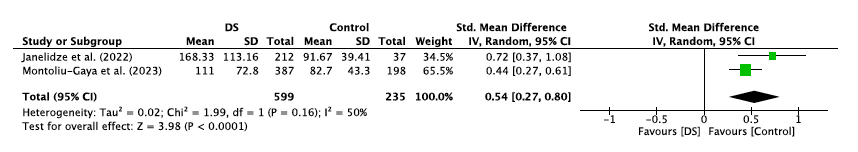


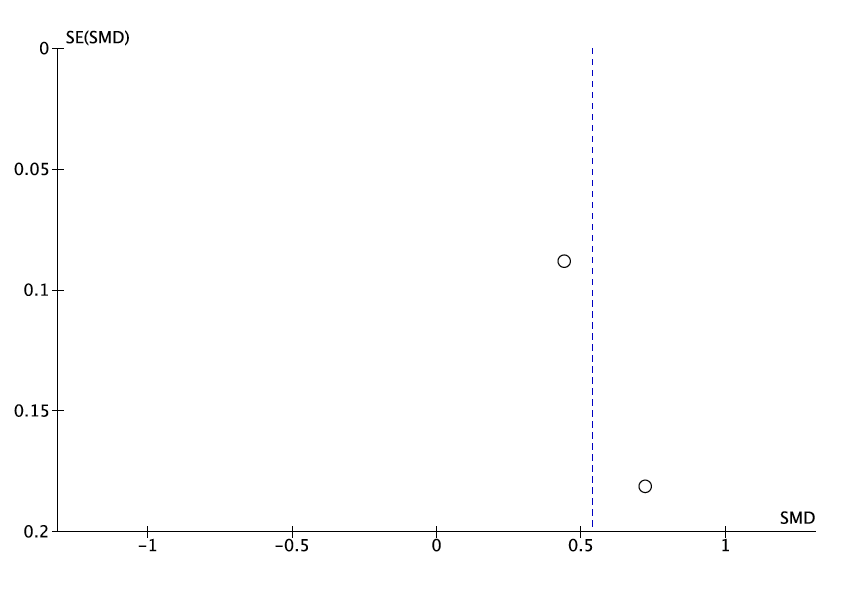


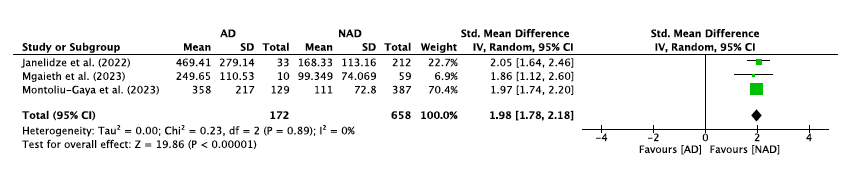
Figure S26 Meta-analysis of studies comparing plasma **GFAP** levels of individuals with **DS** **with AD and without AD**. Abbreviations: DS = Down syndrome, AD = Alzheimer's disease, SD = standard deviation, CI = Confidence Interval, Std. = Standardized


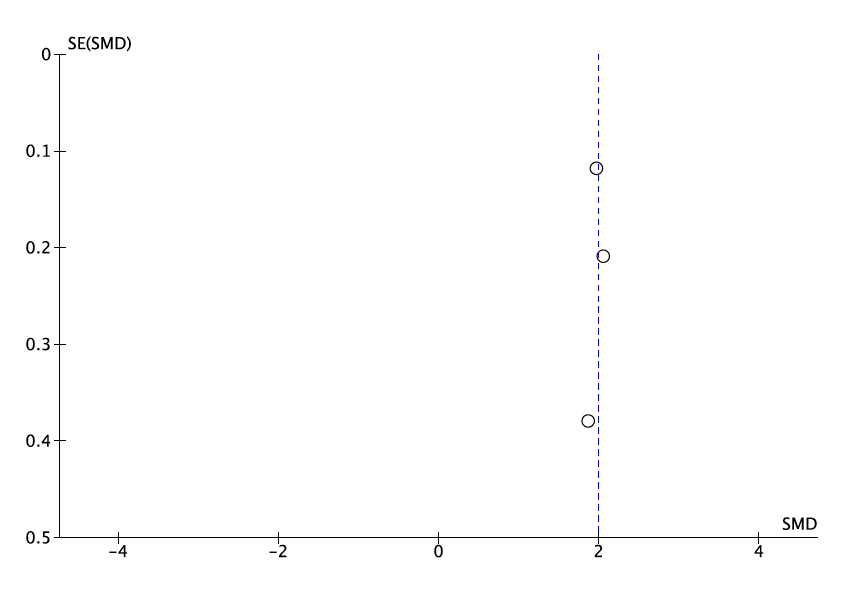


Figure S27 Meta-analysis of studies comparing plasma **GFAP** levels of individuals with **DS** **with prodromal AD and cognitively stable**. Abbreviations: DS = Down syndrome, AD = Alzheimer's disease, SD = standard deviation, CI = Confidence Interval, Std. = Standardized


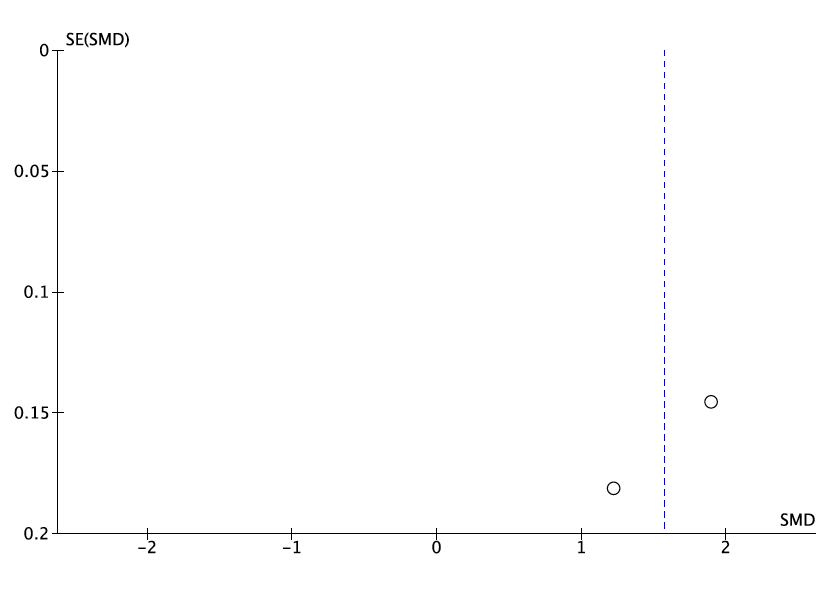

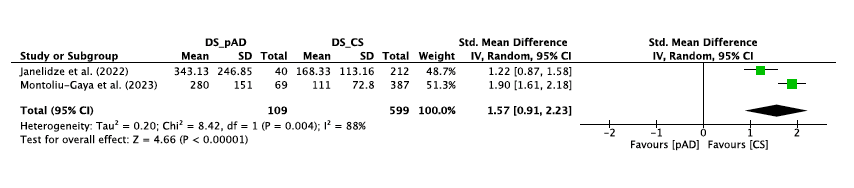


Figure S28 Meta-analysis of studies comparing plasma **GFAP** levels of individuals with **DS** **with AD and prodromal AD**. Abbreviations: DS = Down syndrome, AD = Alzheimer's disease, SD = standard deviation, CI = Confidence Interval, Std. = Standardized


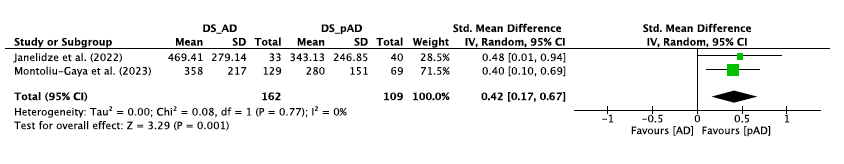


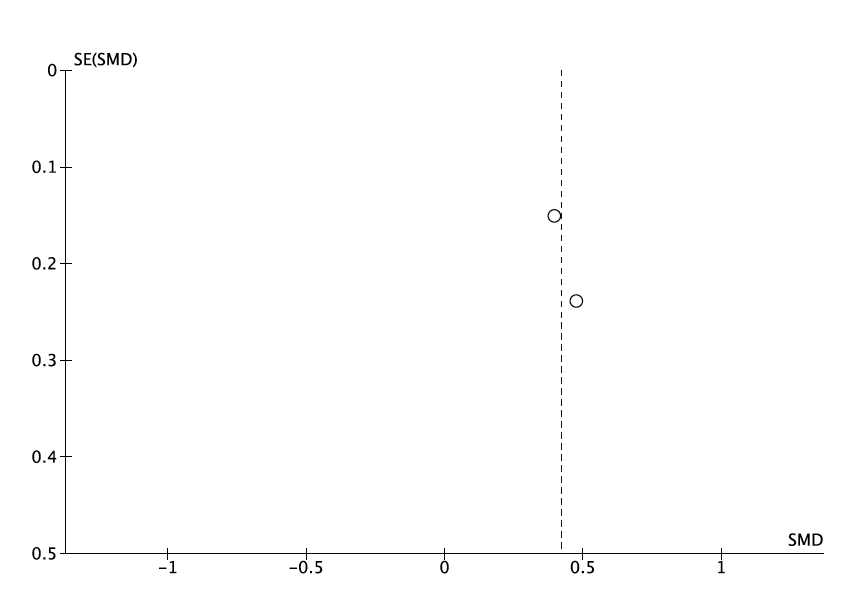


## Sensitivity Analysis


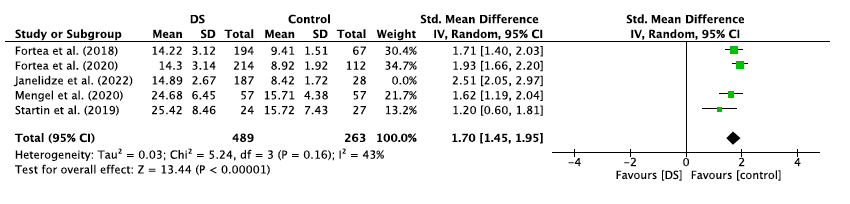
Figure S29 Sensitivity analysis of Meta-analysis of studies comparing plasma **Aβ42** levels of individuals with **DS** and **normal controls**. Abbreviations: DS = Down syndrome, AD = Alzheimer's disease, SD = standard deviation, CI = Confidence Interval, Std. = Standardized


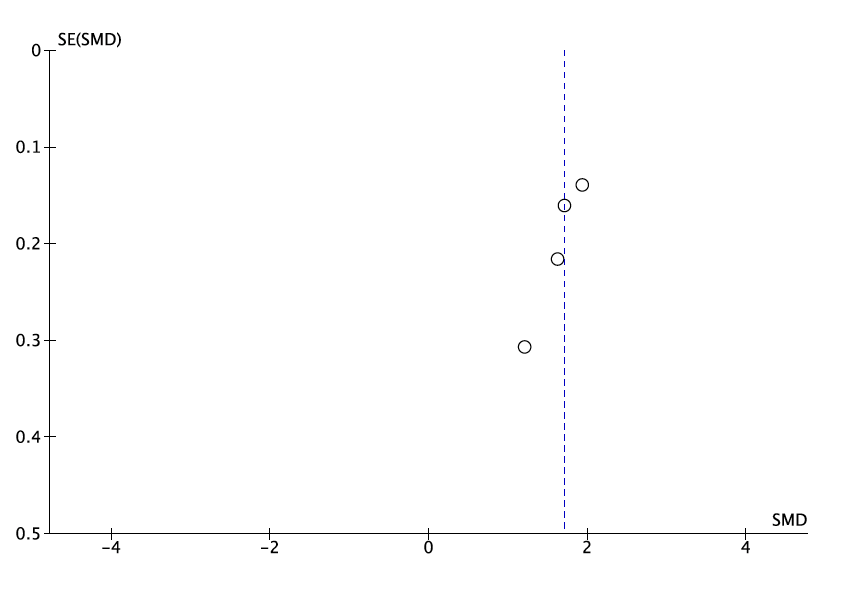


Figure S30 Sensitivity analysis of Meta-analysis of studies comparing plasma **p-tau 181** levels of individuals with **DS with prodromal AD and cognitively stable**. Abbreviations: DS = Down syndrome, AD = Alzheimer's disease, SD = standard deviation, CI = Confidence Interval, Std. = Standardized


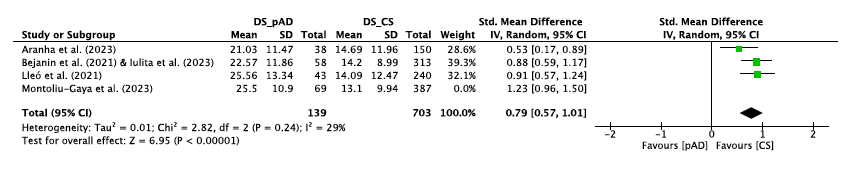


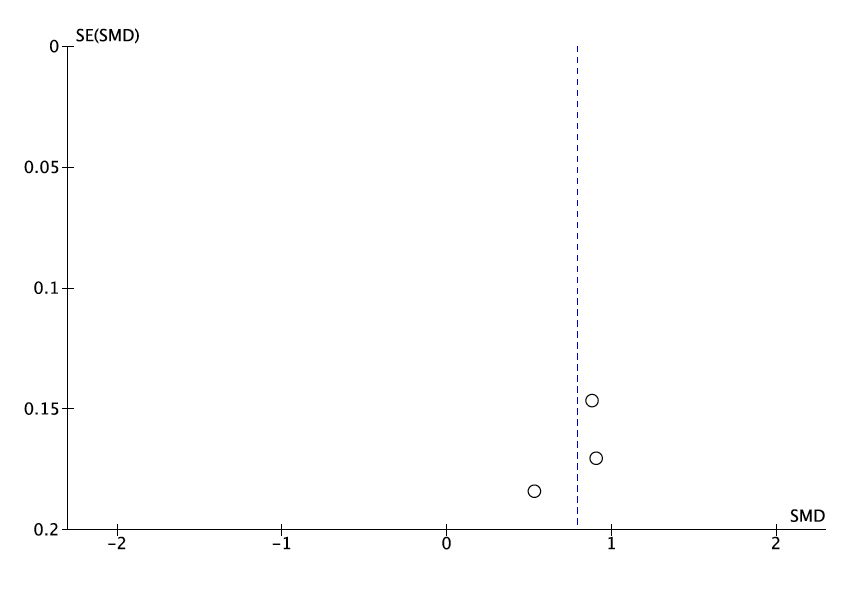


Figure S31 Sensitivity analysis of Meta-analysis of studies comparing plasma **t-tau** levels of individuals with **DS and normal controls**. Abbreviations: DS = Down syndrome, AD = Alzheimer's disease, SD = standard deviation, CI = Confidence Interval, Std. = Standardized


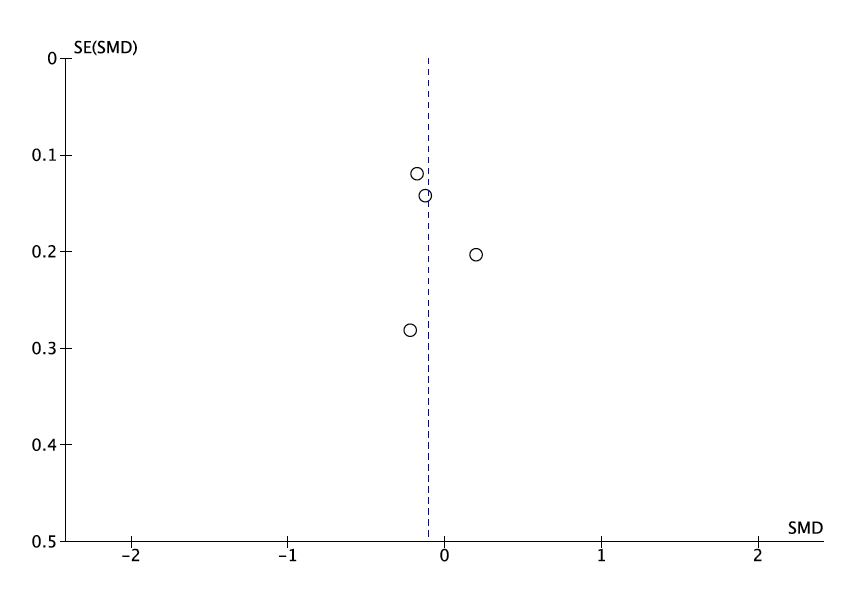

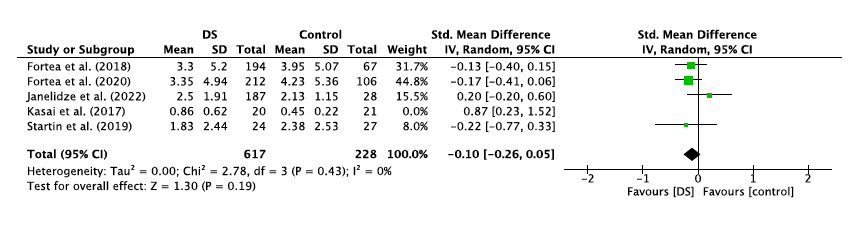


Figure S32 Sensitivity analysis of Meta-analysis of studies comparing plasma **NfL** levels of individuals with **DS and normal controls**. Abbreviations: DS = Down syndrome, AD = Alzheimer's disease, SD = standard deviation, CI = Confidence Interval, Std. = Standardized


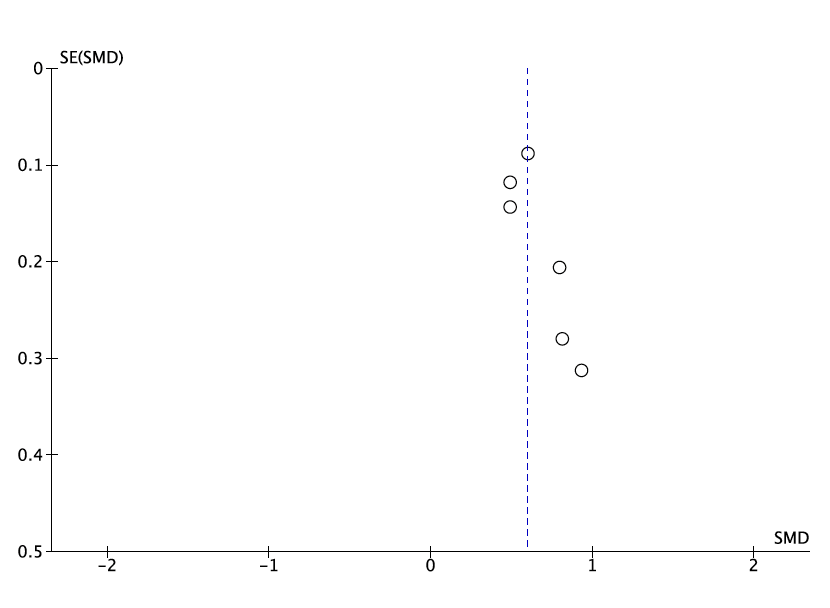

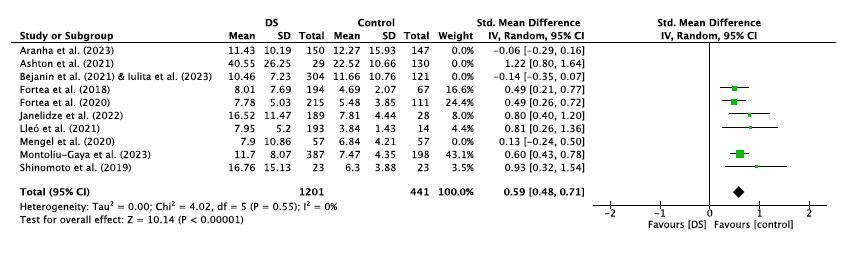


Figure S33 Sensitivity analysis of Meta-analysis of studies comparing plasma **NfL** levels of individuals with **DS with prodromal AD and cognitively stable**. Abbreviations: DS = Down syndrome, AD = Alzheimer's disease, SD = standard deviation, CI = Confidence Interval, Std. = Standardized


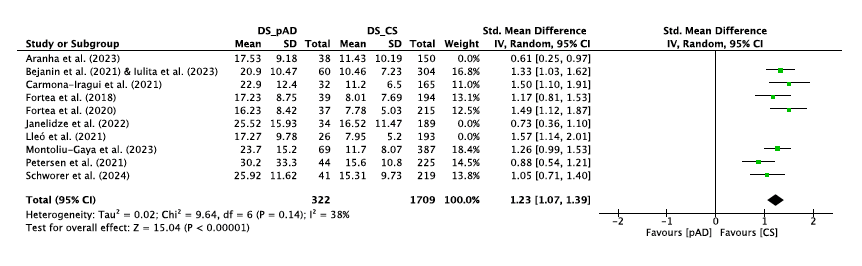


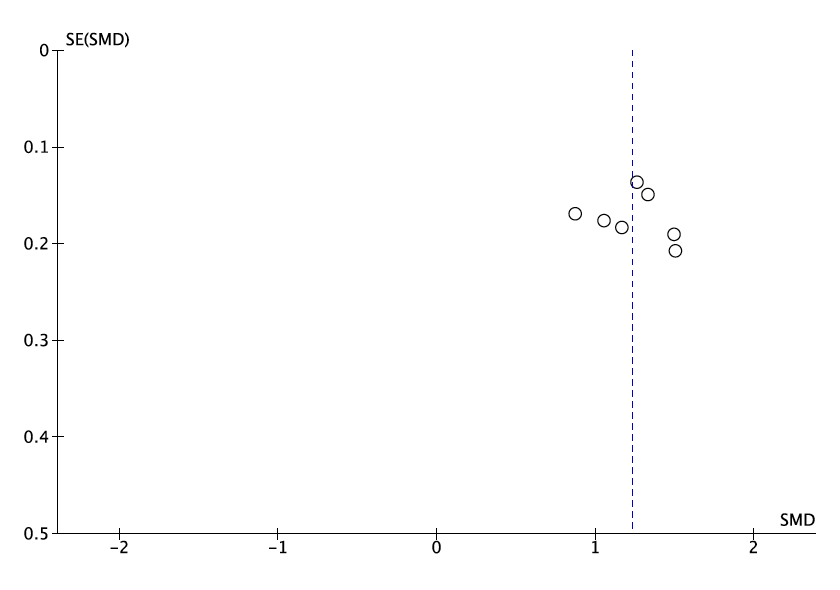


## References

1. Ashton NJ, Janelidze S, Al Khleifat A, et al. A multicentre validation study of the diagnostic value of plasma neurofilament light. *Nature Communications*. 2021/06/07 2021;12(1):3400. doi:10.1038/s41467-021-23620-z

2. Mehta PD, Patrick BA, Miller DL, Coyle PK, Wisniewski T. A Sensitive and Cost-Effective Chemiluminescence ELISA for Measurement of Amyloid-β 1-42 Peptide in Human Plasma. *Journal of Alzheimer's Disease*. 2020;78(3)doi:10.3233/JAD-200861

3. Handen B, Clare I, Laymon C, et al. Acute Regression in Down Syndrome. *Brain Sci*. Aug 23 2021;11(8)doi:10.3390/brainsci11081109

4. Weber GE, Koenig KA, Khrestian M, et al. An Altered Relationship between Soluble TREM2 and Inflammatory Markers in Young Adults with Down Syndrome: A Preliminary Report. *J Immunol*. Mar 1 2020;204(5):1111-1118. doi:10.4049/jimmunol.1901166

5. Meguid NA, Hemimi M, Elpatrik G, Fouad-Elhady EA, Dardir AA, Ahmed HH. Analysis of Specific Serum Markers for Early Prediction of Alzheimer's Disease in Adolescents with Down Syndrome. *Indian Journal of Clinical Biochemistry*. 2024/04/02 2024;doi:10.1007/s12291-024-01206-y

6. Bejanin A, Iulita MF, Vilaplana E, et al. Association of Apolipoprotein E ɛ4 Allele With Clinical and Multimodal Biomarker Changes of Alzheimer Disease in Adults With Down Syndrome. *JAMA Neurology*. 2021;78(8)doi:10.1001/jamaneurol.2021.1893

7. Iulita MF, Bejanin A, Vilaplana E, et al. Association of biological sex with clinical outcomes and biomarkers of Alzheimer's disease in adults with Down syndrome. *Brain Commun*. 2023;5(2):fcad074. doi:10.1093/braincomms/fcad074

8. Aranha MR, Iulita MF, Montal V, et al. Basal forebrain atrophy along the Alzheimer's disease continuum in adults with Down syndrome. *Alzheimer's & Dementia*. 2023;19(11)doi:10.1002/alz.12999

9. Lee N-C, Yang S-Y, Chieh J-J, et al. Blood Beta-Amyloid and Tau in Down Syndrome: A Comparison with Alzheimer’s Disease. *Frontiers in Aging Neuroscience*. 2017;8doi:10.3389/fnagi.2016.00316

10. Morsiani C, Bacalini MG, Collura S, et al. Blood circulating miR-28-5p and let-7d-5p associate with premature ageing in Down syndrome. *Mechanisms of Ageing and Development*. 2022/09/01/ 2022;206:111691. doi:<https://doi.org/10.1016/j.mad.2022.111691>

11. Raha-Chowdhury R, Henderson JW, Raha AA, et al. Choroid Plexus Acts as Gatekeeper for TREM2, Abnormal Accumulation of ApoE, and Fibrillary Tau in Alzheimer’s Disease and in Down Syndrome Dementia. *Journal of Alzheimer's Disease*. 2019;69(1)doi:10.3233/JAD-181179

12. Fortea J, Vilaplana E, Carmona-Iragui M, et al. Clinical and biomarker changes of Alzheimer's disease in adults with Down syndrome: a cross-sectional study. *The Lancet*. 2020;395(10242):1988-1997. doi:10.1016/S0140-6736(20)30689-9

13. Schworer EK, Handen BL, Petersen M, et al. Cognitive and functional performance and plasma biomarkers of early Alzheimer's disease in Down syndrome. *Alzheimer's & Dementia: Diagnosis, Assessment & Disease Monitoring*. 2024;16(2)doi:10.1002/dad2.12582

14. Veteleanu A, Pape S, Davies K, et al. Complement dysregulation and Alzheimer's disease in Down syndrome. *Alzheimers Dement*. Apr 2023;19(4):1383-1392. doi:10.1002/alz.12799

15. Fang WQ, Hwu WL, Chien YH, et al. Composite Scores of Plasma Tau and β-Amyloids Correlate with Dementia in Down Syndrome. *ACS Chem Neurosci*. Jan 15 2020;11(2):191-196. doi:10.1021/acschemneuro.9b00585

16. Aranha MR, Montal V, van den Brink H, et al. Cortical microinfarcts in adults with Down syndrome assessed with 3T-MRI. *Alzheimers Dement*. Jun 2024;20(6):3906-3917. doi:10.1002/alz.13797

17. Hendrix JA, Airey DC, Britton A, et al. Cross-Sectional Exploration of Plasma Biomarkers of Alzheimer's Disease in Down Syndrome: Early Data from the Longitudinal Investigation for Enhancing Down Syndrome Research (LIFE-DSR) Study. *J Clin Med*. Apr 28 2021;10(9)doi:10.3390/jcm10091907

18. Janelidze S, Christian BT, Price J, et al. Detection of Brain Tau Pathology in Down Syndrome Using Plasma Biomarkers. *JAMA Neurol*. Aug 1 2022;79(8):797-807. doi:10.1001/jamaneurol.2022.1740

19. Carmona-Iragui M, Alcolea D, Barroeta I, et al. Diagnostic and prognostic performance and longitudinal changes in plasma neurofilament light chain concentrations in adults with Down syndrome: a cohort study. *Lancet Neurol*. Aug 2021;20(8):605-614. doi:10.1016/s1474-4422(21)00129-0

20. Mengel D, Liu W, Glynn RJ, et al. Dynamics of plasma biomarkers in Down syndrome: the relative levels of Aβ42 decrease with age, whereas NT1 tau and NfL increase. *Alzheimers Res Ther*. Mar 19 2020;12(1):27. doi:10.1186/s13195-020-00593-7

21. Moreau M, Carmona-Iragui M, Altuna M, et al. DYRK1A and Activity-Dependent Neuroprotective Protein Comparative Diagnosis Interest in Cerebrospinal Fluid and Plasma in the Context of Alzheimer-Related Cognitive Impairment in Down Syndrome Patients. *Biomedicines 2022, Vol 10, Page 1380*. 2022;10(6)doi:10.3390/biomedicines10061380

22. Raha-Chowdhury R, Henderson JW, Raha AA, et al. Erythromyeloid-Derived TREM2: A Major Determinant of Alzheimer's Disease Pathology in Down Syndrome. *J Alzheimers Dis*. 2018;61(3):1143-1162. doi:10.3233/jad-170814

23. Mgaieth F, Baksh RA, Startin CM, et al. Exploring semantic verbal fluency patterns and their relationship to age and Alzheimer's disease in adults with Down syndrome. *Alzheimers Dement*. Nov 2023;19(11):5129-5137. doi:10.1002/alz.13097

24. Yang J, Hu L, Zhang Y, Shi Y, Jiang W, Song C. Gesell Developmental Schedules scores and the relevant factors in children with Down syndrome. *J Pediatr Endocrinol Metab*. Apr 28 2020;33(4):539-546. doi:10.1515/jpem-2019-0236

25. Raha AA, Ghaffari SD, Henderson J, et al. Hepcidin Increases Cytokines in Alzheimer's Disease and Down's Syndrome Dementia: Implication of Impaired Iron Homeostasis in Neuroinflammation. *Front Aging Neurosci*. 2021;13:653591. doi:10.3389/fnagi.2021.653591

26. Koenig KA, Bekris LM, Ruedrich S, et al. High-resolution functional connectivity of the default mode network in young adults with down syndrome. *Brain Imaging Behav*. Aug 2021;15(4):2051-2060. doi:10.1007/s11682-020-00399-z

27. Araya P, Kinning KT, Coughlan C, et al. IGF1 deficiency integrates stunted growth and neurodegeneration in Down syndrome. *Cell Rep*. Dec 27 2022;41(13):111883. doi:10.1016/j.celrep.2022.111883

28. Raha-Chowdhury R, Raha AA, Henderson J, et al. Impaired Iron Homeostasis and Haematopoiesis Impacts Inflammation in the Ageing Process in Down Syndrome Dementia. *J Clin Med*. Jun 29 2021;10(13)doi:10.3390/jcm10132909

29. Kasai T, Tatebe H, Kondo M, et al. Increased levels of plasma total tau in adult Down syndrome. *PLOS ONE*. 2017;12(11)doi:10.1371/journal.pone.0188802

30. Delabar JM, Lagarde J, Fructuoso M, et al. Increased plasma DYRK1A with aging may protect against neurodegenerative diseases. *Translational Psychiatry 2023 13:1*. 2023;13(1)doi:10.1038/s41398-023-02419-0

31. Manti S, Cutrupi MC, Cuppari C, et al. Inflammatory biomarkers and intellectual disability in patients with Down syndrome. *Journal of Intellectual Disability Research*. 2018;62(5):382-390. doi:<https://doi.org/10.1111/jir.12470>

32. Grasso M, Fidilio A, L’Episcopo F, et al. Low TGF-β1 plasma levels are associated with cognitive decline in Down syndrome. *Frontiers in Pharmacology*. 2024;15doi:10.3389/fphar.2024.1379965

33. Dekker AD, Vermeiren Y, Carmona-Iragui M, et al. Monoaminergic impairment in Down syndrome with Alzheimer's disease compared to early-onset Alzheimer's disease. *Alzheimers Dement (Amst)*. 2018;10:99-111. doi:10.1016/j.dadm.2017.11.001

34. Conti E, Gregori M, Radice I, et al. Multifunctional liposomes interact with Abeta in human biological fluids: Therapeutic implications for Alzheimer's disease. *Neurochemistry International*. 2017/09/01/ 2017;108:60-65. doi:<https://doi.org/10.1016/j.neuint.2017.02.012>

35. Pentz R, Iulita MF, Ducatenzeiler A, et al. Nerve growth factor (NGF) pathway biomarkers in Down syndrome prior to and after the onset of clinical Alzheimer's disease: A paired CSF and plasma study. *Alzheimers Dement*. Apr 2021;17(4):605-617. doi:10.1002/alz.12229

36. Strydom A, Heslegrave A, Startin CM, et al. Neurofilament light as a blood biomarker for neurodegeneration in Down syndrome. *Alzheimer's Research & Therapy*. 2018/04/10 2018;10(1):39. doi:10.1186/s13195-018-0367-x

37. DiProspero N, Sathishkumar M, Janecek J, et al. Neurofilament light chain concentration mediates the association between regional medial temporal lobe structure and memory in adults with Down syndrome. *Alzheimer's & dementia (Amsterdam, Netherlands)*. 2024;16(1)doi:10.1002/dad2.12542

38. Lleó A, Zetterberg H, Pegueroles J, et al. Phosphorylated tau181 in plasma as a potential biomarker for Alzheimer’s disease in adults with Down syndrome. *Nature Communications*. 2021/07/14 2021;12(1):4304. doi:10.1038/s41467-021-24319-x

39. Montoliu-Gaya L, Alcolea D, Ashton NJ, et al. Plasma and cerebrospinal fluid glial fibrillary acidic protein levels in adults with Down syndrome: a longitudinal cohort study. *EBioMedicine*. Apr 2023;90:104547. doi:10.1016/j.ebiom.2023.104547

40. Fortea J, Carmona-Iragui M, Benejam B, et al. Plasma and CSF biomarkers for the diagnosis of Alzheimer's disease in adults with Down syndrome: a cross-sectional study. *Lancet Neurol*. Oct 2018;17(10):860-869. doi:10.1016/s1474-4422(18)30285-0

41. Startin CM, Ashton NJ, Hamburg S, et al. Plasma biomarkers for amyloid, tau, and cytokines in Down syndrome and sporadic Alzheimer's disease. *Alzheimers Res Ther*. Mar 21 2019;11(1):26. doi:10.1186/s13195-019-0477-0

42. Rafii MS, Donohue MC, Matthews DC, et al. Plasma Neurofilament Light and Alzheimer’s Disease Biomarkers in Down Syndrome: Results from the Down Syndrome Biomarker Initiative (DSBI). *Journal of Alzheimer's Disease*. 2019;70(1)doi:10.3233/JAD-190322

43. Shinomoto M, Kasai T, Tatebe H, et al. Plasma neurofilament light chain: A potential prognostic biomarker of dementia in adult Down syndrome patients. *PLoS One*. 2019;14(4):e0211575. doi:10.1371/journal.pone.0211575

44. Stern AM, Van Pelt KL, Liu L, et al. Plasma NT1-tau and Aβ(42) correlate with age and cognitive function in two large Down syndrome cohorts. *Alzheimers Dement*. Dec 2023;19(12):5755-5764. doi:10.1002/alz.13382

45. Petersen ME, Rafii MS, Zhang F, et al. Plasma Total-Tau and Neurofilament Light Chain as Diagnostic Biomarkers of Alzheimer’s Disease Dementia and Mild Cognitive Impairment in Adults with Down Syndrome. *Journal of Alzheimer's Disease*. 2021;79(2)doi:10.3233/JAD-201167

46. Moni F, Petersen ME, Zhang F, et al. Probing the proteome to explore potential correlates of increased Alzheimer's‐related cerebrovascular disease in adults with Down syndrome. *Alzheimer's & Dementia*. 2022;18(10)doi:10.1002/alz.12627

47. Petersen ME, Zhang F, Schupf N, et al. Proteomic profiles for Alzheimer's disease and mild cognitive impairment among adults with Down syndrome spanning serum and plasma: An Alzheimer's Biomarker Consortium–Down Syndrome (ABC–DS) study. *Alzheimer's & Dementia: Diagnosis, Assessment & Disease Monitoring*. 2020;12(1)doi:10.1002/dad2.12039

48. O'Bryant SE, Zhang F, Silverman W, et al. Proteomic profiles of incident mild cognitive impairment and Alzheimer's disease among adults with Down syndrome. *Alzheimer's & Dementia: Diagnosis, Assessment & Disease Monitoring*. 2020;12(1):e12033. doi:<https://doi.org/10.1002/dad2.12033>

49. Petersen M, Zhang F, Krinsky-McHale SJ, et al. Proteomic profiles of prevalent mild cognitive impairment and Alzheimer's disease among adults with Down syndrome. *Alzheimer's & Dementia: Diagnosis, Assessment & Disease Monitoring*. 2020;12(1)doi:10.1002/dad2.12023

50. Tatebe H, Kasai T, Ohmichi T, et al. Quantification of plasma phosphorylated tau to use as a biomarker for brain Alzheimer pathology: pilot case-control studies including patients with Alzheimer’s disease and down syndrome. *Molecular Neurodegeneration 2017 12:1*. 2017;12(1)doi:10.1186/s13024-017-0206-8

51. Oeckl P, Wagemann O, Halbgebauer S, et al. Serum Beta-Synuclein Is Higher in Down Syndrome and Precedes Rise of pTau181. *Ann Neurol*. Jul 2022;92(1):6-10. doi:10.1002/ana.26360

52. Pape SE, al Janabi T, Ashton NJ, et al. The reliability and validity of DSM 5 diagnostic criteria for neurocognitive disorder and relationship with plasma neurofilament light in a down syndrome population. *Scientific Reports*. 2021/06/29 2021;11(1):13438. doi:10.1038/s41598-021-92887-5

53. Sánchez-Moreno B, Zhang L, Mateo G, et al. Voxel-based dysconnectomic brain morphometry with computed tomography in Down syndrome. *Ann Clin Transl Neurol*. Jan 2024;11(1):143-155. doi:10.1002/acn3.51940

54. Huggard D, Kelly L, Ryan E, et al. Increased systemic inflammation in children with Down syndrome. *Cytokine*. 2020/03/01/ 2020;127:154938. doi:<https://doi.org/10.1016/j.cyto.2019.154938>

55. Tarani L, Carito V, Ferraguti G, et al. Neuroinflammatory Markers in the Serum of Prepubertal Children with Down Syndrome. *Journal of Immunology Research*. 2020;2020(1):6937154. doi:<https://doi.org/10.1155/2020/6937154>

56. Powers RK, Culp-Hill R, Ludwig MP, et al. Trisomy 21 activates the kynurenine pathway via increased dosage of interferon receptors. *Nature Communications*. 2019/10/18 2019;10(1):4766. doi:10.1038/s41467-019-12739-9

57. Edwards NC, Lao PJ, Alshikho MJ, et al. Cerebrovascular disease is associated with Alzheimer’s plasma biomarker concentrations in adults with Down syndrome. *Brain Communications*. 2024;6(5):fcae331. doi:10.1093/braincomms/fcae331

58. Hamlett ED, Goetzl EJ, Ledreux A, et al. Neuronal exosomes reveal Alzheimer's disease biomarkers in Down syndrome. *Alzheimers Dement*. May 2017;13(5):541-549. doi:10.1016/j.jalz.2016.08.012
